# Comparative genomics of *Mycobacterium mucogenicum* and *Mycobacterium neoaurum* clade members emphasizing tRNA and non-coding RNA

**DOI:** 10.1186/s12862-019-1447-7

**Published:** 2019-06-18

**Authors:** Phani Rama Krishna Behra, B. M. Fredrik Pettersson, Sarbashis Das, Santanu Dasgupta, Leif A. Kirsebom

**Affiliations:** Department of Cell and Molecular Biology, Biomedical Centre, Box 596, SE-751 24 Uppsala, Sweden

**Keywords:** Mycobacterial genomes, Comparative mycobacterial genomics, Non-coding RNA in mycobacteria, tRNA genes, Expression of tRNA and non-coding RNA

## Abstract

**Background:**

Mycobacteria occupy various ecological niches and can be isolated from soil, tap water and ground water. Several cause diseases in humans and animals. To get deeper insight into our understanding of mycobacterial evolution focusing on tRNA and non-coding (nc)RNA, we conducted a comparative genome analysis of *Mycobacterium mucogenicum* (*Mmuc*) and *Mycobacterium neoaurum* (*Mneo*) clade members.

**Results:**

Genome sizes for *Mmuc-* and *Mneo*-clade members vary between 5.4 and 6.5 Mbps with the complete *Mmuc*^T^ (type strain) genome encompassing 6.1 Mbp. The number of tRNA genes range between 46 and 79 (including one pseudo tRNA gene) with 39 tRNA genes common among the members of these clades, while additional tRNA genes were probably acquired through horizontal gene transfer. Selected tRNAs and ncRNAs (RNase P RNA, tmRNA, 4.5S RNA, Ms1 RNA and 6C RNA) are expressed, and the levels for several of these are higher in stationary phase compared to exponentially growing cells. The rare tRNA^Ile^TAT isoacceptor and two for mycobacteria novel ncRNAs: the *Lactobacillales*-derived GOLLD RNA and a homolog to the antisense *Salmonella typhimurium* phage Sar RNA, were shown to be present and expressed in certain *Mmuc*-clade members.

**Conclusions:**

Phages, IS elements, horizontally transferred tRNA gene clusters, and phage-derived ncRNAs appears to have influenced the evolution of the *Mmuc*- and *Mneo*-clades. While the number of predicted coding sequences correlates with genome size, the number of tRNA coding genes does not. The majority of the tRNA genes in mycobacteria are transcribed mainly from single genes and the levels of certain ncRNAs, including RNase P RNA (essential for the processing of tRNAs), are higher at stationary phase compared to exponentially growing cells. We provide supporting evidence that Ms1 RNA represents a mycobacterial 6S RNA variant. The evolutionary routes for the ncRNAs RNase P RNA, tmRNA and Ms1 RNA are different from that of the core genes.

**Electronic supplementary material:**

The online version of this article (10.1186/s12862-019-1447-7) contains supplementary material, which is available to authorized users.

## Background

Mycobacteria are divided into rapid and slow growing mycobacteria, RGM and SGM. Among SGM, *Mycobacterium tuberculosis* (*Mtb*) causes tuberculosis (TB) while the non-pathogenic RGM *Mycobacterium smegmatis* (*Msmeg*) is frequently used as a mycobacterial model system. Both SGM and RGM can inhabit various environmental reservoirs such as ground and tap water, soil, animals and humans. They can form aggregates and biofilms and appear to have a growth advantage in water that contains disinfecting agents [1–4].

The RGM *Mycobacterium mucogenicum* (*Mmuc*) was first reported as a *Mycobacterium chelonae*-like bacteria in connection with a peritonitis outbreak in 1982 [5–7]. On the basis of partial sequencing of the 16S rRNA gene *Mmuc* was later proposed to be a new taxon and the name reflects that it is highly mucoid when grown on solid media [3, 8–10]. *M. mucogenicum* together with *Mycobacterium phocaicum* (*Mpho*) and *Mycobacterium aubagnense* (*Maub*) constitute the *Mmuc*-clade [3, 9–11]. These RGM are water-borne and *Mmuc* is claimed to be one of the most abundant non-tuberculosis mycobacteria (NTM) in tap water and it is found in sewage and in hospital water systems. They are opportunistic pathogens and have been demonstrated to be associated with various infections and they show high tolerance against first-line anti-tuberculosis drugs such as isoniazid, rifampin and pyrazinamide [3, 5, 7, 9–20]. Moreover, strains of the phylogenetically close mycobacteria *Mycobacterium neoaurum* (*Mneo*) and *Mycobacterium cosmeticum* (*Mcos*), which both belong to the *Mneo*-clade, have also been isolated from patients suffering from various infections [21–24]. Together this emphasizes the importance of this group of NTMs and provided an incentive for a comparative genomic analysis of these closely related species.

The tRNA genes are implicated to be targets for integration of foreign DNA [25, 26] and in bacteria their number and gene synteny vary. For example, the 4.6 Mbp long *Escherichia coli* K12 MG1655 genome carries 86 while *Streptomyces coelicolor* (genome size, 8.7 Mbp) encodes 65 tRNA genes [27, 28]. On the basis of available data, it appears that the number of tRNA genes in several mycobacteria such as *Mtb*H37Rv (genome size, 4.4 Mbp) and *Msmeg* (genome size, 7 Mbp) do not exceed 50 [29–32]. Given the seemingly low number of tRNA genes among mycobacteria we were interested to understand whether this is also the case for other mycobacteria. Moreover, a preliminary survey of tRNA gene organization in mycobacteria such as *Mtb*H37Rv and *Msmeg* indicates that many tRNA are transcribed from single genes, which is in contrast to other bacteria, *e.g*., *E. coli* and *Bacillus subtilis* [27, 29, 30, 33]. We were therefore interested in finding whether this might also apply to other mycobacteria focusing on the NTM discussed above. In this context, our understanding of non-coding (nc) RNA in mycobacteria, apart from *Mtb*H37Rv and *Msmeg* [34], is scarce. Hence, we also decided to conduct a comparative genomic analysis focusing on ncRNA genes in *Mmuc*- and *Mneo*-clade members to understand absence and presence of ncRNA genes with respect to phylogeny and their evolution. We were particularly interested to study ncRNAs with implicated functions in RNA processing, gene regulation and gene expression such as RNase P RNA, the catalytic subunit of the tRNA processing endoribonuclease P, RNase P [35].

We report that genome sizes among *Mmuc*- and *Mneo*-clade members, including the complete genome of the *Mmuc* type strain, vary between approximately 5.4 to 6.5 Mbp (see also the NCBI database). Comparative genomic analysis (17 genomes in total) provide some insight into how some of the characteristics of these genomes such as genome size, common and unique genes, and horizontal gene transfer (HGT) might be manifested as phenotypic differences. Specifically, the number of tRNA genes vary between 46 and 79 (including one pseudo tRNA gene) with *Maub* having the highest number; 39 tRNA genes were predicted to be present in all studied mycobacteria. For those species having higher number of tRNA genes our data suggest that several have been acquired through HGT and we show that some of these are expressed. Moreover, predicted ncRNA genes range between 28 and 46, and while some are species specific others are also present in *Mtb*H37Rv and *Msmeg*, and for some ncRNAs, *e.g*. RNase P RNA, the evolutionary routes are different from that of the core genes. Finally, we provide supportive data suggesting that one of the ncRNAs, Ms1 RNA, represents a mycobacterial 6S RNA variant, which in other bacteria acts as a global regulator.

## Methods

### Strains and cultivation

The *M. mucogenicum* DSM44124 (*Mmuc*^T^), *M. phocaicum* DSM45104 (*Mpho*^T^), *M. aubagnense* DSM45150 (*Maub*^T^), *M. neoaurum* DSM44074 (*Mneo*^T^) and *M. cosmeticum* DSM44829 (*Mcos*^T^) type strains were obtained from the Deutsche Sammlung von Mikroorganismen und Zellkulturen in Germany and grown under conditions as recommended by the supplier. The DNA was isolated and purified as previously described [31].

### Genome sequencing, assembly and annotation

The *Mmuc*^T^ whole genome sequencing was performed at the NGI-Uppsala Genome Center using the PacBio technology and the average read length was 10936 bp and the median coverage ≈101x. The genomes of the other four *Mycobacterium* spp., *Mpho*^T^, *Maub*^T^, *Mneo*^T^ and *Mcos*^T^, were sequenced at the SNP&SEQ Technology Platform using the Illumina-Hiseq2000 platform at Uppsala University, Uppsala with an average read length of 100 bp and a median coverage of ≈176x. The PacBio-generated reads were assembled using the SMART-analysis HGAP3 assembly pipeline [36] and polished using Quiver (Pacific Biosciences, Menlo Park, CA, USA). The Illumina generated reads were assembled using the A5-assembly pipeline [37, 38] as previously described [31]. For the annotation of rRNA genes we used RNAmmer version 1.2 [39] and for annotation of tRNA genes the tRNAScan-SE program version 1.23 [40]. Small ncRNA genes were predicted using the Rfam version 12.1 database and INFERNAL (version 1.1.2) with a threshold cut off value of ≥34 [41–45].

To predict open reading frames (*i.e*., coding sequences, CDS), we used the PROKKA software (version 1.10; [46]). For functional classification all predicted CDS were subjected to BLASTp against the RAST subsystem database (http://rast.nmpdr.org/, last accessed May 5, 2015; [47] using the BLAST approach [48]).

### Plasmids, Phage DNA and IS-elements

To predict the presence of plasmid sequences, the scaffolds of the different mycobacterial draft genomes were sorted and aligned using the closest reference genome (*Mmuc*^T^; complete genome) and the Mauve software (version 2.3.1 [49]). The aligned scaffolds were subsequently concatenated and subjected to BLAST search against the NCBI plasmid database (ftp://ftp.ncbi.nlm.nih.gov/refseq/release/plasmid/, accessed March 2015; May 2019).

Phage sequences were predicted using the PHAGE database at the PHASTER server (PHAge Searching Tool server; [50, 51]).

The presence of IS elements in the complete *Mmuc*^T^ genome was predicted using the ISsaga server and manually inspected searching for inverted repeats and presence of transposase genes [52].

### Core gene analysis

Translated protein coding sequences (CDS) were extracted for the target genomes and combined with CDSs from available *Mycobacterium* spp. to generate a BLAST database. A reciprocal BLAST was performed using the BLASTp and the PanOCT (version 1.9) with the following parameter settings, identity of at least 45%, query coverage >70% and *e*-value cutoff 1e-05 [32]. The predicted orthologous genes were also subjected to gene synteny analysis using PanOCT [53].

### Phylogenetic analysis and average nucleotide identity

Single gene phylogenies were generated based on the 16S rRNA gene, *rnpB*, *ssrA*, *ffh* and the Ms1 RNA gene. The respective gene sequences were extracted from the five genomes (*Mmuc*^T^, *Mpho*^T^, *Maub*^T^, *Mneo*^T^ and *Mcos*^T^) and 104 publicly available mycobacterial genomes from the NCBI data base. The homologous genes were identified using the PanOCT ortholog. Single (16S rDNA, *rnpB*, *ssrA*, *ffh* and the Ms1 RNA gene) and core genes were aligned using the MAFFT (version 7.147b) software [54]. Phylogenetic trees based on 16S rDNA, core genes and multiple sequence alignments were computed using the FastTree version 2.1.7 [55] and figures were generated using the FigTree Software version 1.3.1 (http://tree.bio.ed.ac.uk/software/figtree/).

The Jspecies tool was used to calculate the average nucleotide identity (ANI) values. ANI values were clustered using an unsupervised hierarchical clustering algorithm and plotted using "R" environment [56–58].

### RNA extraction and northern analysis

*Mmuc*^T^ and *Maub*^T^ were grown in liquid 7H9 medium (including 10% OADC supplement and 0.25% Tween 80) at 37°C and 100 rpm rotation. At exponential (OD_600_ = 0.3-0.5) and stationary (OD_600_ = 5-6) growth phases, cells were pelleted and snap frozen in liquid nitrogen. RNA was extracted following the protocol described elsewhere [59]. Triplicate samples/independent cultures (10 μg RNA from *Maub*^T^ and 15 μg from *Mmuc*^T^ per sample) from each growth phase were then separated on an 8% polyacrylamide gel and electroblotted to a Hybond N+ membrane as previously described [60]. The membranes were then probed with ^32^P-5'-end-labelled DNA oligonucleotide, washed, and exposed to a phosphorimager screen. See Additional file 1: Table S1 for DNA oligonucleotide sequences and hybridization temperatures (for specifics see [60]).

## Results

### Genome assembly and annotation

**Fig. 1:**
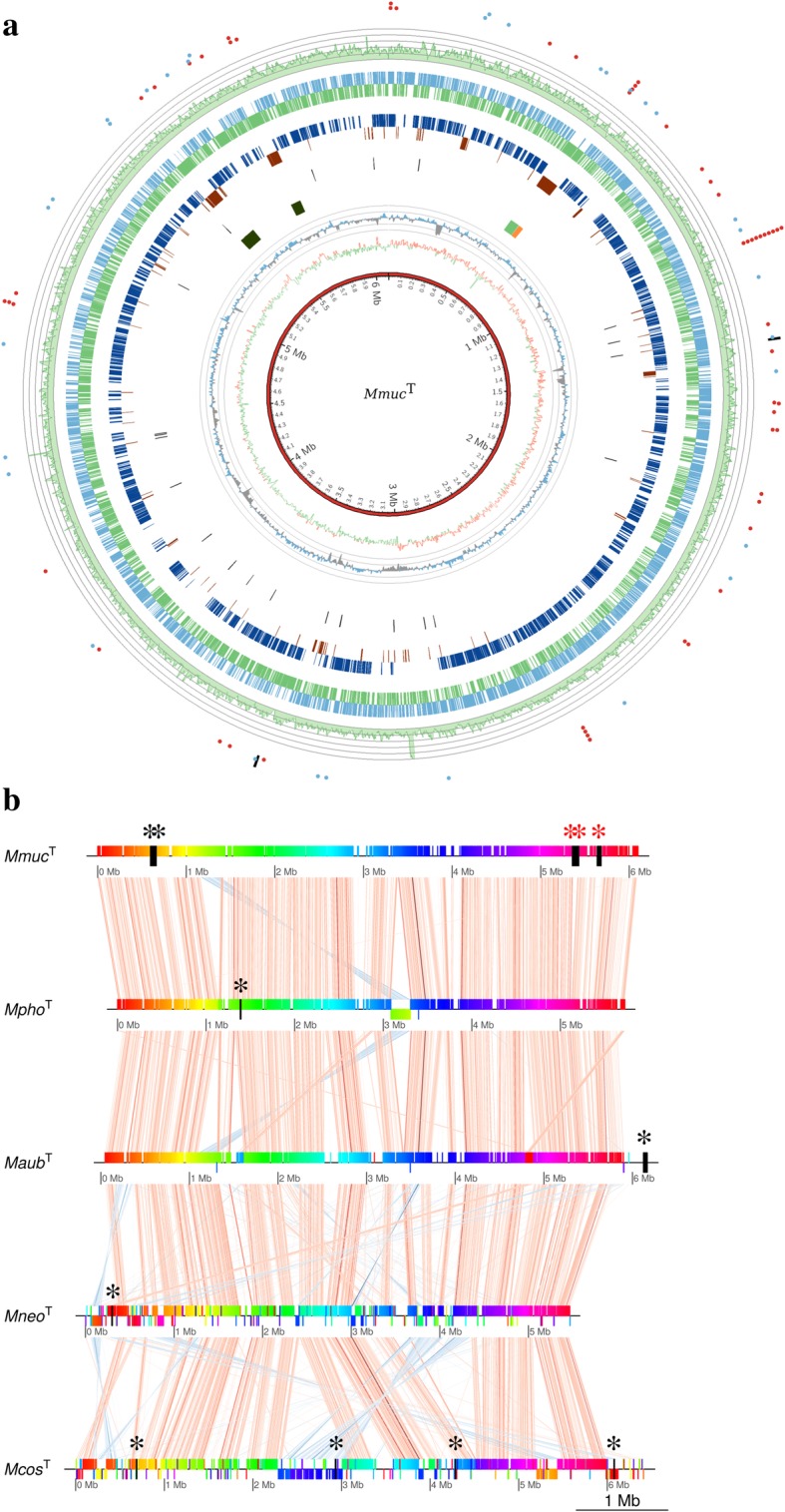
Complete genome of *Mmuc*^T^. **a** Circos plot showing the complete genome sequence of *Mmuc*^T^. The outer to inner circles represent: Red and blue dots represent genome wide distribution of tRNA and ncRNA genes, while the two black lines represent the rRNA operons. Green histogram represents the average sequencing read depth for *Mmuc*^T^, while the overlapping grey circle gives the read depth scale (the distance between the two circles is 50x). The blue and green blocks in the two subsequent circles represent genes that are predicted to be transcribed from the leading and lagging strands, respectively. Dark blue and brown blocks indicate core and unique genes comparing *Mmuc*- and *Mneo*-clade members. Next inner circle, black line marks the positioning of the IS-elements. The four blocks represent the phage sequences. The GC-content is represented by the blue and grey "spikes". It was calculated using a sliding window of 5000 bp and the grey circles give the scale, which oscillates between ±10% of the mean value 67.18%. Next track in red (positive) and green (negative) shows the GC skew using a sliding window of 5000 bp. The inner circle shows the size of the *Mmuc*^T^ genome. Generation of circos plots, see http://circos.ca. **b** Whole-genome alignment for the five type strains *Mmuc*^T^, *Mpho*^T^, *Maub*^T^, *Mneo*^T^ and *Mcos*^T^. Each horizontal block represents one genome and vertical lines between the genome correspond to homologous regions whereas diagonal lines correspond to genome rearrangements (blue lines genomic inversions and white gaps represent insertions/deletions). The black boxes/vertical lines represent phage sequences; single stars in red mark the presence of an intact phage and black stars represent incomplete/questionable pro-phage sequence (see text for details)

The size of the complete *Mmuc*^T^ genome is 6,099,273 while the sizes for the draft *Mpho*^T^, *Maub*^T^, *Mneo*^T^ and *Mcos*^T^ genomes are 5,771,543; 6,191,633; 5,477,923 and 6,446,208 base pairs, respectively (Fig. 1a; Table 1). The GC-content for the five species was calculated to be in the range 66.3% to 68.3%. The complete *Mmuc*^T^ genome includes 5881 protein-coding sequences (CDS), two ribosomal RNA operons, 57 tRNA genes and 33 non-coding (nc) RNA genes. For the other four species, the number of CDS range from 5199 to 6270 genes, which correlates with their respective genome size (Table 1). Moreover, for *Mneo*^T^ and *Mcos*^T^ 46 tRNA genes were identified, while *Mpho*^T^ and *Maub*^T^ carry 60 and 79 (among these one pseudo tRNA gene) tRNA genes, respectively. The Rfam annotated ncRNA genes in *Mpho*^T^, *Maub*^T^, *Mneo*^T^ and *Mcos*^T^ were 45, 41, 34 and 28, respectively (Table 1; in total 14 mycobacteria analyzed). In Table 1 we give a basic description of 14 of the genomes analyzed in this study. Genome assembly, gene annotation and Genbank accession numbers are summarized in Table 1 and Additional file 1: Table S2.

**Table 1:** Summary of genome annotation

Species	Genome size (bp)	(%) GC content	Total number of predicted						Bioproject ID	Accession no
			Scaffolds	CDS	rRNA (5S;23S;16S)	tRNA	ncRNA	Phage		
***M. mucogenicum*** **DSM 44124** ^**a**^ **(Complete)**	6099273	67.2	1	5881	(2;2;2)	57	33	5	PRJNA429429	POTL00000000
*M. sp.* 360MFTsu	6055808	67.2	24	5740	(2;2;2)	56	31	1	PRJNA187944	ARTK01000000
*M. sp.* UNC410	6051129	67.1	31	5731	(2;2;2)	57	34	0	PRJNA234913	JMLM00000000
***M. phocaicum*** **DSM 45104** ^**a**^	5771543	67	80	5515	(2;1;1)	60	45	0	PRJNA429429	POTM00000000
*M. sp.* UNC280	5694455	66.7	48	5455	(2;1;1)	46	33	0	PRJNA234892	JQKU00000000
*M. llatzerense* strain CLUC14	6091591	66.6	103	5880	(3;1;1)	47	35	1	PRJNA273763	JXST00000000
***M. aubagnense*** **DSM 45150** ^**a**^	6191633	66.3	85	5943	(2;1;1)	79	41	5	PRJNA429429	POTN00000000
*M. cosmeticum* DSM 44829	6462090	68.2	5	6281	(2;2;2)	46	29	1	PRJEB5748	CCBB000000000
***M. cosmeticum*** **DSM 44829** ^**a**^	6446208	68.3	40	6270	(2;1;1)	46	28	2	PRJNA429429	POTP00000000
*M. neoaurum* VKM Ac-1815D (Complete)	5438192	66.9	1	5018	(2;2;2)	46	34	2	PRJNA199859	NC_023036
*M. neoaurum* ATCC 25795	5468381	66.7	42	5178	(1;1;1)	46	34	1	PRJNA244824	JMDW00000000
*M. neoaurum* DSM 44074	5504703	66.6	45	5269	(3;1;1)	46	34	1	PRJEB1060	CCDR000000000
***M. neoaurum*** **DSM 44074** ^**a**^	5477923	66.7	27	5199	(2;1;1)	46	34	0	PRJNA429429	POTO00000000
*M. sp.* URHB0044	7483125	66.8	43	7230	(2;1;1)	61	29	2	PRJNA213774	JIAW00000000

All genomes were annotated using PROKKA, except *M. neoaurum* VKM strain *i.e,* NCBI annotation

Species highlighted in bold are representative genomes in this article

*CDS* Coding Sequences, *rRNA* ribosomal RNA, *tRNA* Transfer RNA, *ncRNA* non-coding RNA

^a^ indicates genomes reported in this article

### Genome alignment, presence of plasmid DNA, phages and IS-elements

Whole genome alignment revealed that the close neighboring species *Mmuc*^T^, *Mpho*^T^, *Maub*^T^, *Mneo*^T^ and *Mcos*^T^ share large segments of homology. It appears that *Mmuc*^T^, *Mpho*^T^ and *Maub*^T^ show higher homology compared to *Mneo*^T^ and *Mcos*^T^, while chromosomal rearrangements (insertions and translocations) seem to have happened comparing *Mneo*^T^ and *Mcos*^T^ (Fig. 1b). However, we cannot exclude that this is due to draft genome status. Alignment of the 16 genomes (except *M. sp*. URHB0044) is presented in Additional file 1: Figure S1 (note that we analyzed two versions of *Mcos*^DSM44829^ and *Mneo*^DSM44074^). Unless otherwise stated, below we focus on the five type strains, *Mmuc*^T^, *Mpho*^T^, *Maub*^T^, *Mneo*^T^ and *Mcos*^T^.

We did not detect any plasmids or plasmid fragments in any of the five type strains (*Mmuc*^T^, *Mpho*^T^, *Maub*^T^, *Mneo*^T^ and *Mcos*^T^) while phage sequence stretches were predicted to be present (Table 1; for details see Additional file 1: Table S3). In *Mmuc*^T^ three intact prophage regions were predicted using PHASTER (score, >90; see Methods). These regions were not detected in any of the other four species. Two additional phage segments, classified as "questionable" (score, 70 to 90) and "incomplete" (score, <70), were predicted in *Mmuc*^T^. All these phage sequences likely belong to the *Myocbacterium* phage group since the majority of the CDS show high homology with mycobacterial phage CDS. One of the intact phages positioned at the genomic location of 5.63 to 5.68 Mbps is 45.3 kbp long where 20 out 60 CDS show similarities to the *Mycobacterium* phage Bernal13 (Fig. 1b; [50, 51]).

For the complete *Mmuc*^T^ genome we predicted 23 IS elements (for two of these we were unable to identify inverted repeat sequences) representing eight different types (Additional file 1: Table S4a). IS-elements were also predicted in the *Mpho*^T^, *Maub*^T^, *Mneo*^T^ and *Mcos*^T^ draft genomes (Additional file 1: Table S4b), but their precise locations were not defined. For these four strains the number of IS elements vary and *Mpho*^T^ carries 65 predicted IS elements. The highest number was detected in *Mmuc*^LZSF01^ for which we predicted 85 IS elements (Additional file 1: Table S4b).

Taken together this suggests that phage and IS elements likely have had an impact on the evolution of these type strains.

### Identification of core genes and phylogenetic analysis

To construct phylogenetic trees, we first used 291 core genes present in *Mmuc*^T^, *Mpho*^T^, *Maub*^T^, *Mneo*^T^ and *Mcos*^T^ and 104 mycobacterial genomes available at the NCBI genome database (ftp://ftp.ncbi.nlm.nih.gov/genomes/). Second, to get deeper insights into the phylogenetic relationship within the *Mmuc*- and *Mneo*-clades we identified 2226 core genes predicted to be present in the members of these two clades, including the recently released draft *Mmuc* genomes (strains *Mmuc*^LZSF01^, *Mmuc*^LZLC01^ and *Mmuc*^CSURP2099^; Additional file 1: Figure S1), and in *M. sp*. URHB0044 (in total 17 species; Table 1; *M. sp*. URHB0044 was chosen as an outgroup). Functional classification of the 291 and 2226 core genes revealed that the majority (>60%) of the classified genes belongs to the subsystems "Amino Acids and Derivatives", "Cofactors, Vitamins…Pigments", "Carbohydrates", "Protein Metabolism" and "Fatty Acids, Lipids, and Isoprenoids" (Additional file 2: Figure S2a, b).

**Fig. 2:**
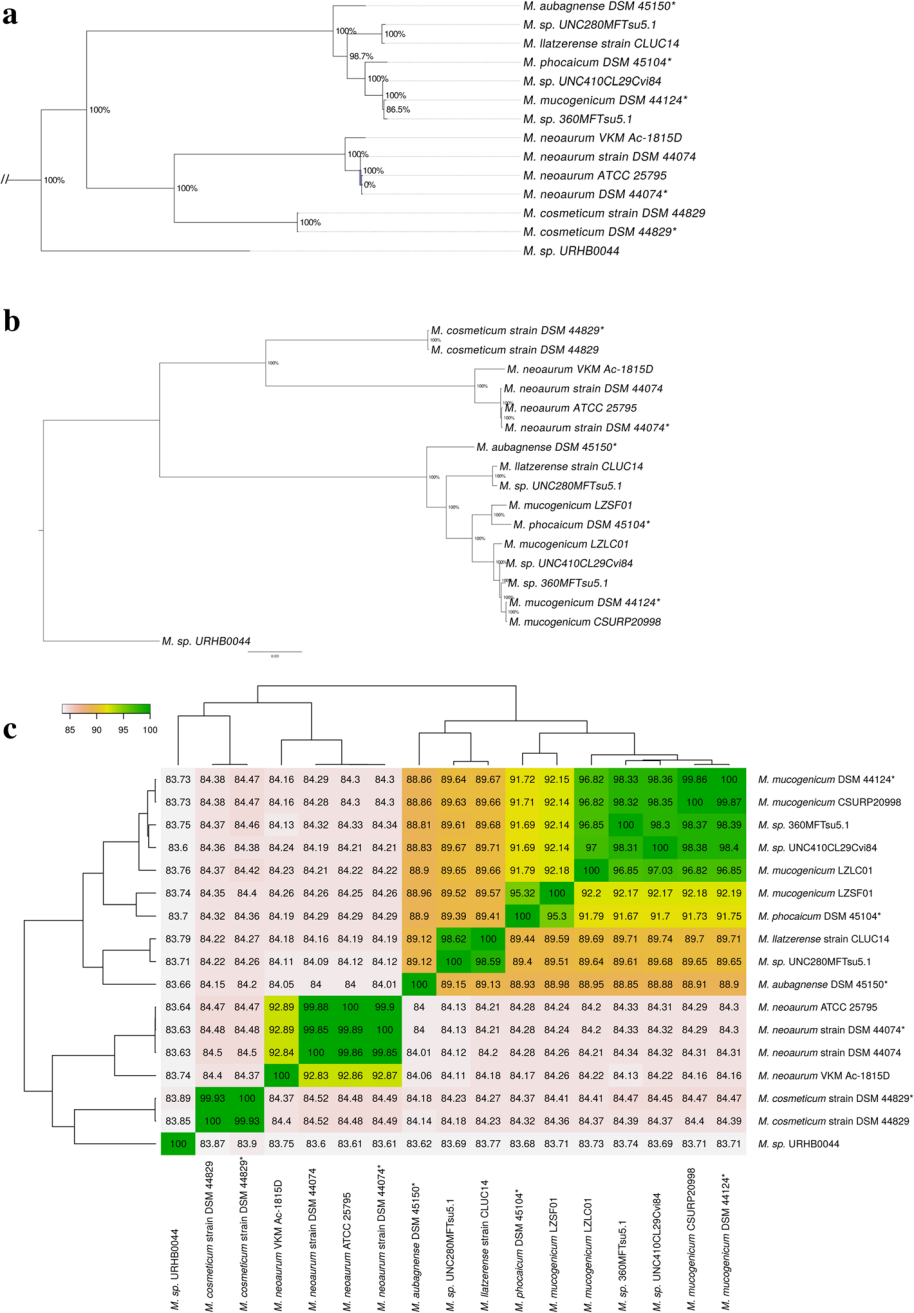
Phylogenetic analysis. **a** Part of the phylogenetic tree based on 291 core genes present in 109 mycobacteria (see also Additional file 4: Figure S2a). *Mark the mycobacterial type strains for which the genomes were sequenced in the present study. **b** Phylogenetic tree based on 2226 core genes predicted to be present in all 17 mycobacteria. In (**a**) and (**b**) the percentage values in the nodes represent boot strap values generated by 1000 cycles. **c** Classification of the 17 mycobacteria using Jspecies. The dendogram show ANI values after clustering using unsupervised hierarchical clustering

Both core phylogenetic trees are in agreement and suggested that *Mmuc*^T^, *Mpho*^T^ and *Maub*^T^ are close neighbors to four other *Mycobacterium* spp., *M. sp.* 360MFT*, M. sp.* UNC410*, M. llatzerense* strain CLUC14 and *M. sp.* UNC280 (referred to as *Msp*360, *Msp*410, *Mllat*, and *Msp*280, respectively; Table 1). Hence it appears that these mycobacteria belong to the *Mmuc*-clade [3]. The *Mneo*^T^ and *Mcos*^T^ cluster together with other *Mcos* and *Mneo* strains in the *Mneo-*clade (Fig. 2a, b; Additional file 2: Figure S3a; [3]). Moreover, *Mmuc*^LZLC01^ and *Mmuc*^CSURP2099^ are positioned close to *Mmuc*^T^ except *Mmuc*^LZSF01^, which is closer to *Mpho*^T^ (Fig. 2a, b). Also, inspection of the *Mneo*-clade suggested that the *Mneo*^VKMAc-1815D^ constitutes a separate branch of *Mneo* (Fig. 2a; see Discussion; for comparison with 16S rDNA phylogeny see Additional file 2: Figure S3a, b).

Analysis of the average nucleotide identity, ANI, also grouped the 17 mycobacteria in accordance with their position in the core (2226 genes) phylogenetic tree (Fig. 2b, c).

Taken together, the phylogenetic data revealed that the positioning of these mycobacteria was in accordance with our current understanding of the mycobacterial phylogeny [3, 61–63; and our unpublished data]. The data further suggested that *Mmuc*^LZSF01^ is a *Mpho* strain and that *Mneo*^VKMAc-1815D^ should be considered as a separate species or *Mneo* subspecies (see Discussion).

### Number of tRNA genes, their organization and expression of selected tRNA genes

**Fig. 3:**
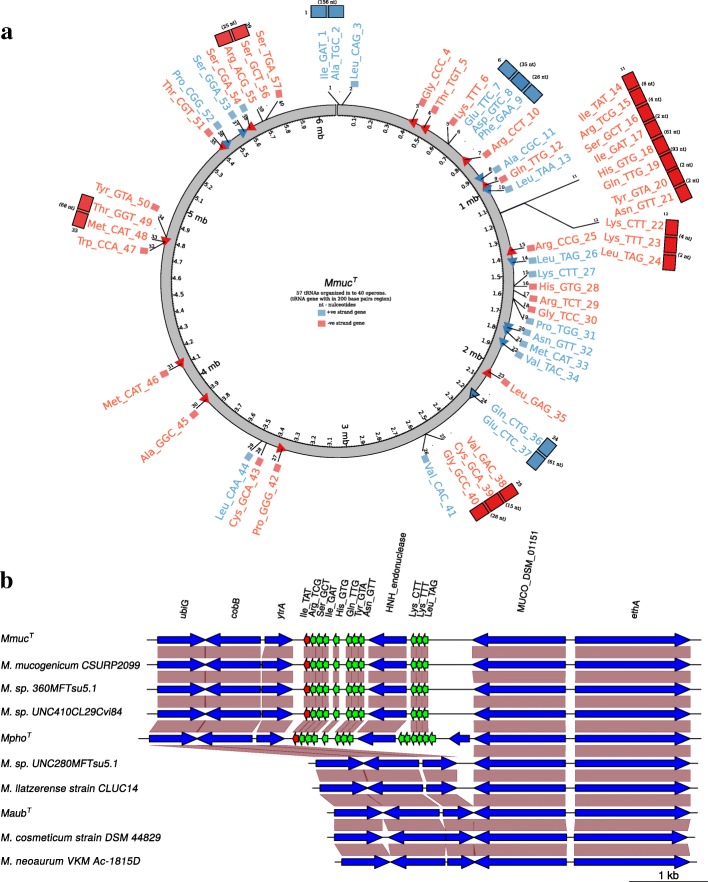
tRNA gene organization and gene synteny. **a** Mapping of the 57 tRNA genes in *Mmuc*^T^. Blue boxes and arrows mark tRNA genes that are transcribed from the leading (+) strand, while red marks those that are transcribed from the lagging (-) strand. Numbers given in parenthesis indicate the numbers of nucleotides separating two tRNA genes. For orientation, we also marked the 40 predicted "transcriptional units", 1 to 40. **b** Gene synteny for "transcription units" 11 and 12, see (**a**), for selected mycobacteria carrying these tRNA genes. The tRNA genes are marked in green except the tRNA^Ile^TAT gene, which is marked in red. The vertical boxes marked in brown highlight homologous genes. Note the presence of the HNH endonuclease gene located between the two tRNA gene clusters (see main text). Generation of gene synteny plots, see http://genoplotr.r-forge.r-project.org/

The number of predicted tRNA genes in *Mmuc*^T^, *Mpho*^T^, *Maub*^T^, *Mneo*^T^ and *Mcos*^T^ were 57, 60, 79 (including one pseudo tRNA gene), 46 and 46, respectively, with *Maub*^T^ having the highest number (Table 1; Fig. 3; Additional file 3: Figure S4a). However, this is not related to which clade these species belong to since *Msp*280 and *Mllat* have 46 and 47, respectively, which is the same as observed for members of the *Mneo*-clade (Fig. 2). The different *Mmuc* strains (*Mmuc*^T^, *Mmuc*^LZSF01^, *Mmuc*^LZLC01^ and *Mmuc*^CSURP2099^) all harbor the same number of predicted tRNA genes with the exception of *Mmuc*^LZSF01^, which is positioned close to *Mpho*^T^ (Table 1; Fig. 2). Of the 119 predicted tRNA genes, 39 are common to members of the *Mmuc*- and *Mneo*-clades and they cover all 20 amino acids (Additional file 3: Figure S4a).

Larger clusters of tRNA genes with more than four genes as in *Mmuc*^T^, *Mpho*^T^ and *Maub*^T^ are uncommon among mycobacteria (Fig. 3; Additional file 3: Figure S4b-e; Table S5; of note, clusters of tRNA genes with more than four genes were not found to be present in the *Mneo*-clade members; see Discussion). Among the 78 tRNA genes in *Maub*^T^ 33 are unique and 32 were predicted to cluster in one region (the "32 tRNA gene" cluster) and transcribed from the same DNA (lagging) strand (tRNA genes constituting this cluster were present on three scaffolds but see Discussion; Additional file 3: Figure S4c).

*Mmuc*^T^ and *Mpho*^T^ carry a structurally different tRNA gene cluster with 11 and 14 tRNA genes, respectively (cf. Fig. 3 and Additional file 3: Figure S4b). Apart from the presence of the three extra genes in *Mpho*^T^, which encode for tRNA^Trp^CCA, tRNA^Gly^TCC and tRNA^Leu^CAA, the gene synteny of this tRNA gene cluster is the same in these two mycobacteria (Fig. 3b). Moreover, the structures (based on their gene sequences) for the majority of the tRNA genes comprising this cluster are the same with one notable exception, the tRNA^His^ gene. In *Mpho*^T^ this tRNA gene carries one additional nucleobase and "mutations" in the region corresponding to the anticodon stem, which results in a distorted stem (Additional file 3: Figure S5; alignment of all predicted tRNA genes). Compared to their corresponding tRNA isoacceptor genes located elsewhere in the genome predicted that all the tRNA isoacceptors originating from the cluster were structurally different. For example, the cluster encoded tRNA^Leu^TAG isoacceptor has a short variable loop whereas the isoacceptor present in all *Mmuc*- and *Mneo*-clade members has an expected "normal" larger variable loop (Additional file 3: Figure S5).

Genes encoding the tRNA isoacceptors tRNA^Ile^TAT and tRNA^Arg^TCG were detected to be present (and part of a larger tRNA gene cluster) only in *Mmuc*^T^ and *Mpho*^T^ (see above), and in *Msp*360, *Msp*410, *Mmuc*^CSURP2099^, *Mmuc*^LZSF01^ and *Mmuc*^LZSC01^. Analysis of the gene synteny of this cluster in these mycobacteria (not shown for *Mmuc*^LZSF01^ and *Mmuc*^LZLC01^) predicted the presence of an HNH endonuclease gene within this cluster while this ortholog is missing in the other ten mycobacteria (Fig. 3b). The finding that *Maub*^T^ lacks this tRNA gene cluster, in combination with that it is positioned closer to the ancestor (Fig. 2a, b; Additional file 2: Figure S3a) than the other *Mmuc*-clade members argues for the possibility that the tRNA gene cluster might have been acquired after *Maub*^T^ diverged from the other *Mmuc*-clade members.

**Fig. 4:**
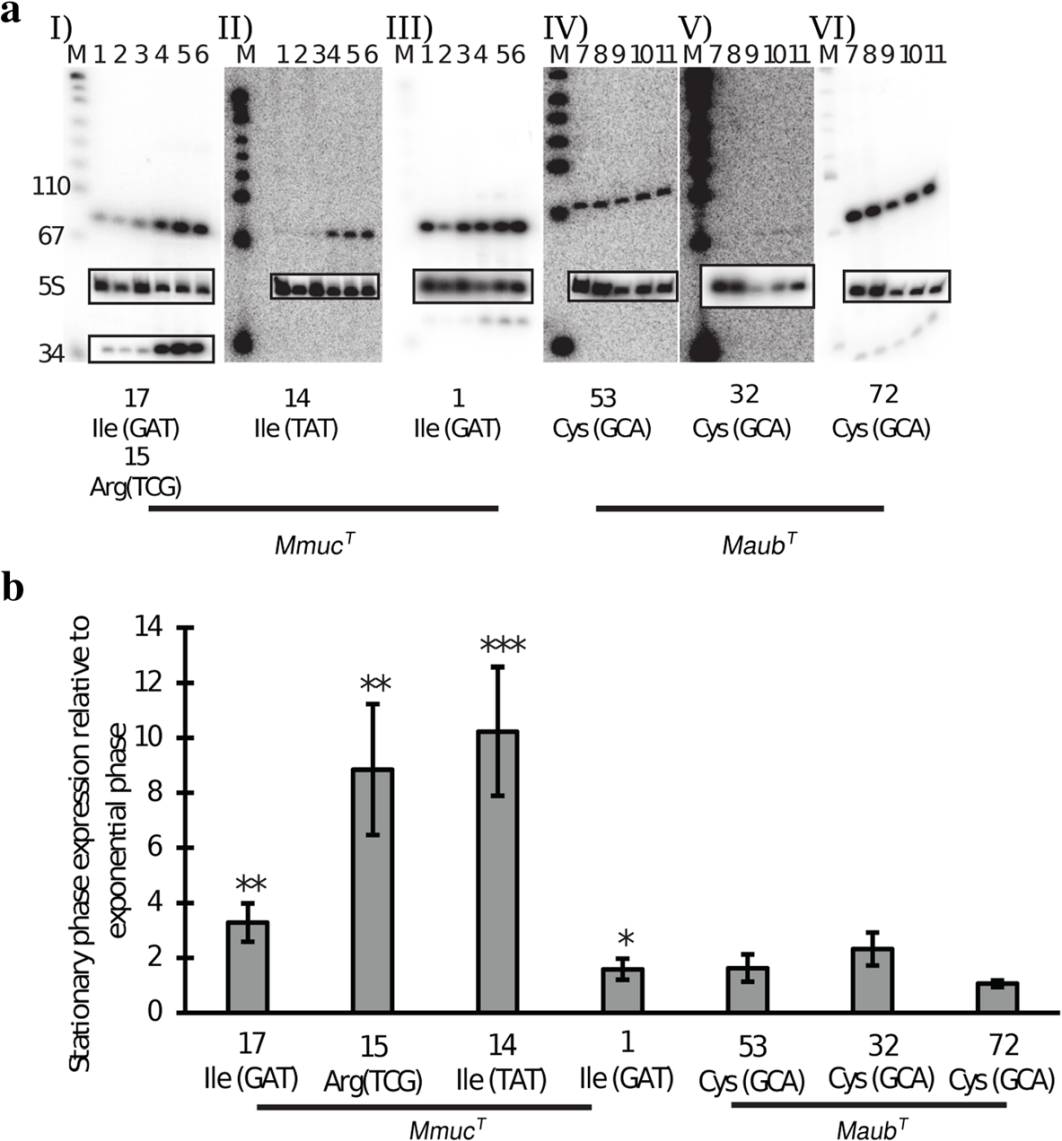
Levels of selected tRNAs at different growth stages. **a** Northern blots of selected tRNAs encoded from the *Mmuc*^T^ "transcriptional unit" 11 and tRNA^Ile^GAT ["transcriptional unit" 11 located near *oriC*, 17 corresponds to Ile_GAT_17 and 15 corresponds to Arg_TCG_15, see Fig. 3a; panels, I – III] and the three *Maub*^T^ tRNA^Cys^GCA [Cys_GCA_53, Cys_GCA_32 and Cys_GCA_72, see (**a**) and Additional file 3: Figure S4c; panels, IV-VI]. The RNA was extracted from stationary and exponential growing cells as described in Methods. M indicates a pUC19/MspI size marker with selected sizes indicated to the left of (I). Lanes 1-3 and 7-8 represent RNA from exponentially grown cells and lanes 4-6 and 9-11 represent RNA from stationary cells. The identities of the detected tRNAs are marked below the images. The boxed insets show the respective blots probed for 5S rRNA as indicated to the left of panel I. The lower panel I inset represents the same blot probed against tRNA^Arg^TCG. **b** Levels of tRNAs in *Mmuc*^T^ and *Maub*^T^ in stationary phase relative to exponentially growing cells. The bar plot shows a quantification of the Northern blot data shown in Fig. 5a. The amino acid three-letter code and the corresponding anticodon triplet mark the tRNA isoacceptor variants. The numbers on the x-axis corresponds to the numbering in (**a**), *e.g*. 17 correspond to Ile_GAT_17 and as indicated. The experiment was performed as outlined in Methods and values represent the mean and errors based on three independent experiments. Statistical significance using Student’s unpaired, two-tailed t-test; *: *p*< 0.05; **: *p* < 0.01; ***: *p*< 0.001

To understand whether the tRNA isoacceptors encoded from the tRNA gene cluster are expressed we focused on tRNA^Ile^TAT, tRNA^Arg^TCG and tRNA^Ile^GAT and included the tRNA^Ile^GAT isoacceptor encoded from another location in *Mmuc*^T^. On the basis of the data presented in Fig. 4 we conclude that these four tRNA isoacceptors are expressed and their levels are higher in cells in stationary phase than in exponentially growing cells.

We previously reported the presence of two tRNA^Cys^GCA genes, one of which is commonly present in mycobacteria while the other is present in some RGM [31]. Two tRNA^Cys^GCA genes were also predicted to be present in the members of the *Mmuc*- and *Mneo*-clades while three were predicted for *Maub*^T^ with one located within the "32 tRNA gene cluster". Gene alignment revealed that the structure of the third tRNA^Cys^GCA gene differs from the other two isoacceptors (see above; Additional file 3: Figures S4c and S5). All three *Maub*^T^ tRNA^Cys^ isoacceptor genes were expressed and the levels were similar in exponentially growing and stationary cells, if anything, slightly increased in stationary phase cells (Fig. 4; see also the Discussion).

It should be noted that genes encoding tRNA^SelCys^ or *selB* appear not to be present in any of the *Mmuc*- and *Mneo*-clade member genomes. Also, no tRNA genes were predicted to be positioned in the ribosomal RNA operons.

### Aminoacyl-tRNA synthetases, AARS

**Fig. 5:**
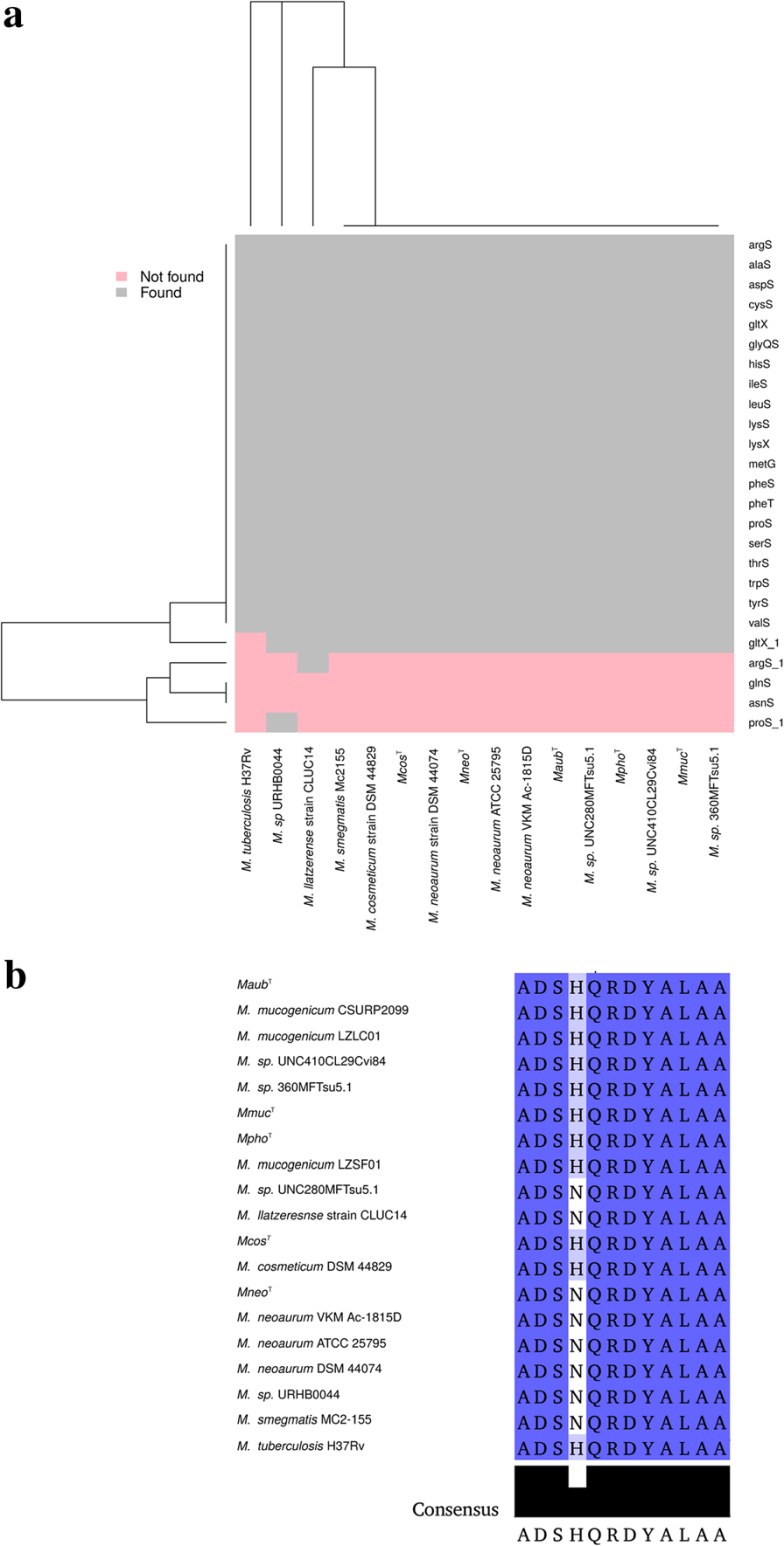
Presence of aminoacyl tRNA synthetase genes and comparison of peptide encoded by tmRNA. **a** Heat-map showing presence (grey) and absence (pink) of aminoacyl-tRNA synthetase genes in *Mmuc*- and *Mneo*-clade members and *Mtb*H37Rv and *Msmeg*MC2-155 as indicated. **b** The peptide sequence for the proteolysis tag encoded by tmRNA as indicated

The tRNAs depend on the aminoacyl-tRNA synthetases (AARSs) for their function. We therefore analyzed members of the *Mmuc*- and *Mneo*-clades for the presence of AARS genes (Fig. 5a; Additional file 3: Table S6a). While 18 AARS genes were predicted to be present in these genomes we were unable to identify any homologous genes for asparaginyl-tRNA synthetase (AsnRS) and glutaminyl-tRNA synthetase (GlnRS) in keeping with previous findings [64, 65]. For bacteria that lack AsnRS and GlnRS genes, tRNA charging of asparagine and glutamine can be accommodated using the tRNA-dependent amidotransferase pathway (Adt), which includes GatCAB [66]. Accordingly, *gatCAB* homologs were predicted in the 16 genomes suggesting that the Adt pathway is operating in these mycobacteria (Additional file 3: Table S6b; of note, *gatA* was not predicted to be present in *Msp*410 possibly due to draft genome status).

The *Mtb* isoleucyl-tRNA synthetase (IleRS) is eukaryotic like [67]. Hence, we were interested in understanding whether this is also the case for the members of the *Mmuc*- and *Mneo*-clades. Only a single IleRS gene copy was predicted to be present in these mycobacteria and our analysis suggested that all encode a eukaryotic like IleRS (Additional file 3: Figure S6a).

We also predicted genes encoding AARS paralogs in some of the mycobacteria (Additional file 3: Supplementary text; Figure S6b-f; Table S6a, c) and genes encoding cyclodipeptide synthetases (CDPSs) in the *Mneo* strains (Additional file 3: Figure S7a, b). In *Mtb*H37Rv this enzyme can hijack aminoacylated tRNAs resulting in formation of cyclodipeptides that subsequently are used for mycocyclosin biosynthesis [68–71]. Prediction of the CDPS gene in *Mneo* strains suggests that it can be present in both SGM and RGM.

### Non-coding RNAs

Non-coding RNAs (ncRNAs) were predicted for members of the *Mmuc*- and *Mneo*-clades as well as *M. sp*. URBH0044 using the Rfam database (Fig. 1 and Table 1; Additional file 4: Table S7). Using these 17 mycobacterial genomes, we predicted 46 ncRNAs which grouped into eight different categories: 1) small sRNAs, 2) antisense RNAs, 3) gene, ribozyme (RNase P RNA), 4) cis-regulatory riboswitches, 5) cis-regulatory thermoregulators, 6) cis-regulatory RNAs, 7) Introns and 8) gene, *e.g*. tmRNA. Of these, 18 ncRNA genes were predicted to be present in all 17 species/strains while others were predicted to be present in and unique to a specific species: ctRNA_p42d and Sar RNA in *Mmuc*^T^, GOLLD (Giant, Ornate, Lake- and *Lactobacillales*-Derived; [72]) RNA in *Maub*^T^, Intron gpII RNA in *Mpho*^T^ and cis-reg *ykkC-yxkD* RNA in *Mcos*^T^ (Additional file 4: Figure S8; Table S7). The size for the corresponding ncRNAs ranged from approximately 50 nucleotides (Ms_AS-5) to roughly 400 nucleotides (RNase P RNA, tmRNA) and >400 nucleotides (GOLLD RNA; Additional file 4: Figure S8b). Below we focus on RNase P RNA, tmRNA, 4.5S RNA, GOLLD RNA, Ms1 RNA ("6S RNA"), 6C RNA and Sar RNA.

### RNase P RNA, tmRNA and 4.5S RNA

**Fig. 6:**
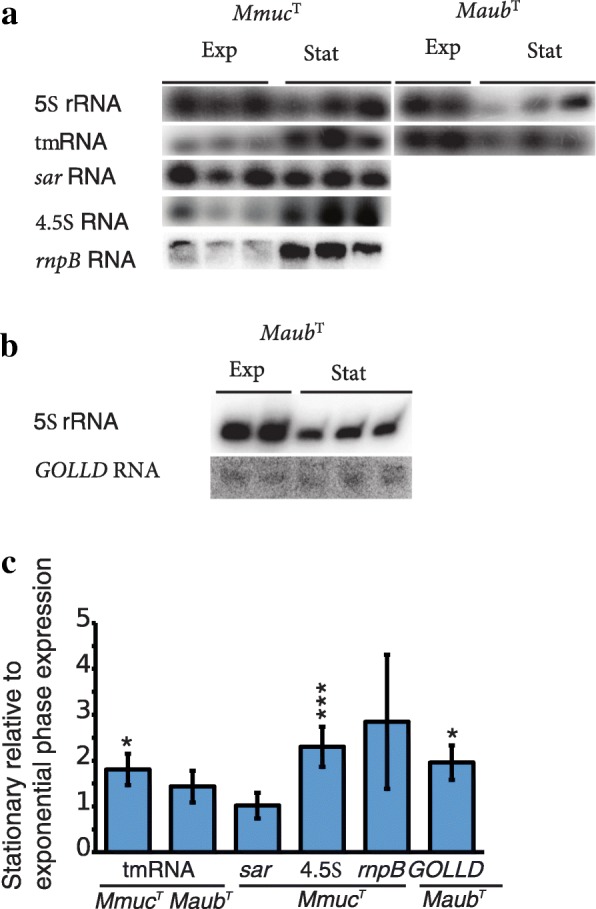
Expression of selected ncRNA genes in *Mmuc*^T^ and *Maub*^T^*.* **a** Representative gels showing levels of 5S rRNA, tmRNA, *sar* RNA, 4.5S RNA and *rnpB* (RNase P) RNA in stationary and exponentially growing *Mmuc*^T^ and *Maub*^T^ cells as indicated (see also Additional file 4: Figure S13). **b** Representative gels showing levels of 5S rRNA and GOLLD RNA in stationary and exponentially growing *Maub*^T^ cells. **c** Levels of tmRNA, *sar* RNA, 4.5S RNA, *rnpB* (RNase P RNA) and GOLLD RNA in stationary cells vs exponentially growing cells in *Mmuc*^T^ and *Maub*^T^ as indicated. The experiments [see (**a**) and (**b**)] were performed as outlined in Methods and values represent the mean and errors based on three independent experiments. Statistical significance using Student’s unpaired, two-tailed t-test; *: *p* < 0.05; ***: *p*< 0.001

As expected genes encoding RNase P RNA (*rnpB*), tmRNA (*ssrA*) and 4.5S RNA (*ffs*) were identified in the genomes for all *Mmuc*- and *Mneo*-clade members. Alignments suggest that *ssrA* and *ffs* are well conserved whereas *rnpB* show more diversity (Additional file 4: Figure S9a-f) consistent with it being a good biomarker for mycobacterial species identification [73]. We also noted that the *ssrA* (tmRNA) encoded C-terminal protein tag is well conserved compared to *Mtb*H37Rv and *Msmeg*MC^2^-155 with variation only at the fourth position (His or Asn; Fig. 5b). Analyzing the location of the genes encoding proteins that interact with these RNAs [the C5 protein (*rnpA*, RNase P), SmpB (*smpB*, tmRNA) and the Ffh protein (*ffh*, 4.5S RNA)] revealed that, as in other bacteria (*e.g*., *E. coli*), the protein coding genes are separated relative to the chromosomal locations for the respective RNA coding genes *rnpB*, *ssrA* and *ffs* (Additional file 4: Figure S9a, c, e). This raises the question how the expression of these gene pairs is regulated, which warrants further studies. In the meantime, measuring the levels of expression of RNase P RNA, tmRNA and 4.5S RNA by northern analysis showed higher levels in stationary *Mmuc*^T^ and *Maub*^T^ cells than in exponentially growing cells (Fig. 6; Additional file 4: Figure S10; for *Maub*^T^ we only measured the levels for tmRNA).

We generated phylogenetic trees for *rnpB*, *ssrA* and *ffs* extracted from 113 mycobacterial genomes (Additional file 4: Figure S9g-i). Interestingly, in comparison to the core phylogeny (Additional file 2: Figure S3a) these three ncRNA genes position the *Mmuc*-clade and in particular the *Mneo* strains closer to the mycobacterial ancestor. Moreover, the *rnpB* and *ssrA* present in *Mmuc*-clade members have the *Mneo* homologs as their ancestors. For *ssrA*, *Mmuc*- and *M. chelonae*-clade members, and *Mcos*^T^ share the same ancestor, *Mneo*. Noteworthy, based on core phylogeny *M. chelonae*-clade members are considered to be the earliest diverging mycobacterial lineage (Additional file 2: Figure S3a [61–63]; unpublished). In conclusion, it appears that the evolutionary route for these ncRNAs is different compared to the core genes.

### GOLLD RNA

The large GOLLD (>400 nucleotides long; Additional file 4: Figure S11a) RNA was only predicted to be present in *Maub*^T^. The predicted gene is part of two scaffolds. However, its gene synteny is supported by the presence of the GOLLD RNA gene on one contig in two other *Mycobacterium* spp., *e.g*. *Mycobacterium abscessus* M24 and *Mycobacterium conceptionense* strain MLE (Additional file 4: Figure S11a; NCBI database; we cannot conclusively exclude the presence of multiple GOLLD RNA gene copies; see also the Discussion). The gene is located within the "32 tRNA cluster" with tRNA genes present both up- and downstream of the GOLLD RNA gene. In addition, a pseudo tRNA gene is predicted to be located within the GOLLD RNA gene (Additional file 4: Figure S11b).

Northern blot analysis suggested that GOLLD RNA is expressed in *Maub*^T^ at a low level, as judged from the weak signal. The level was two-fold higher in stationary phase compared to exponentially growing cells (Fig. 6b, c; Additional file 4: Figure S10) following the same trend as for RNase P RNA, tmRNA and 4.5S RNA (see above). No expression of the predicted pseudo tRNA gene located within the GOLLD RNA gene could be detected, in spite of putative σ^B^ promoters upstream of the pseudo tRNA gene (Additional file 4: Figure S11a; see Discussion).

### Ms1 RNA ("6S RNA") and 6C RNA

**Fig. 7:**
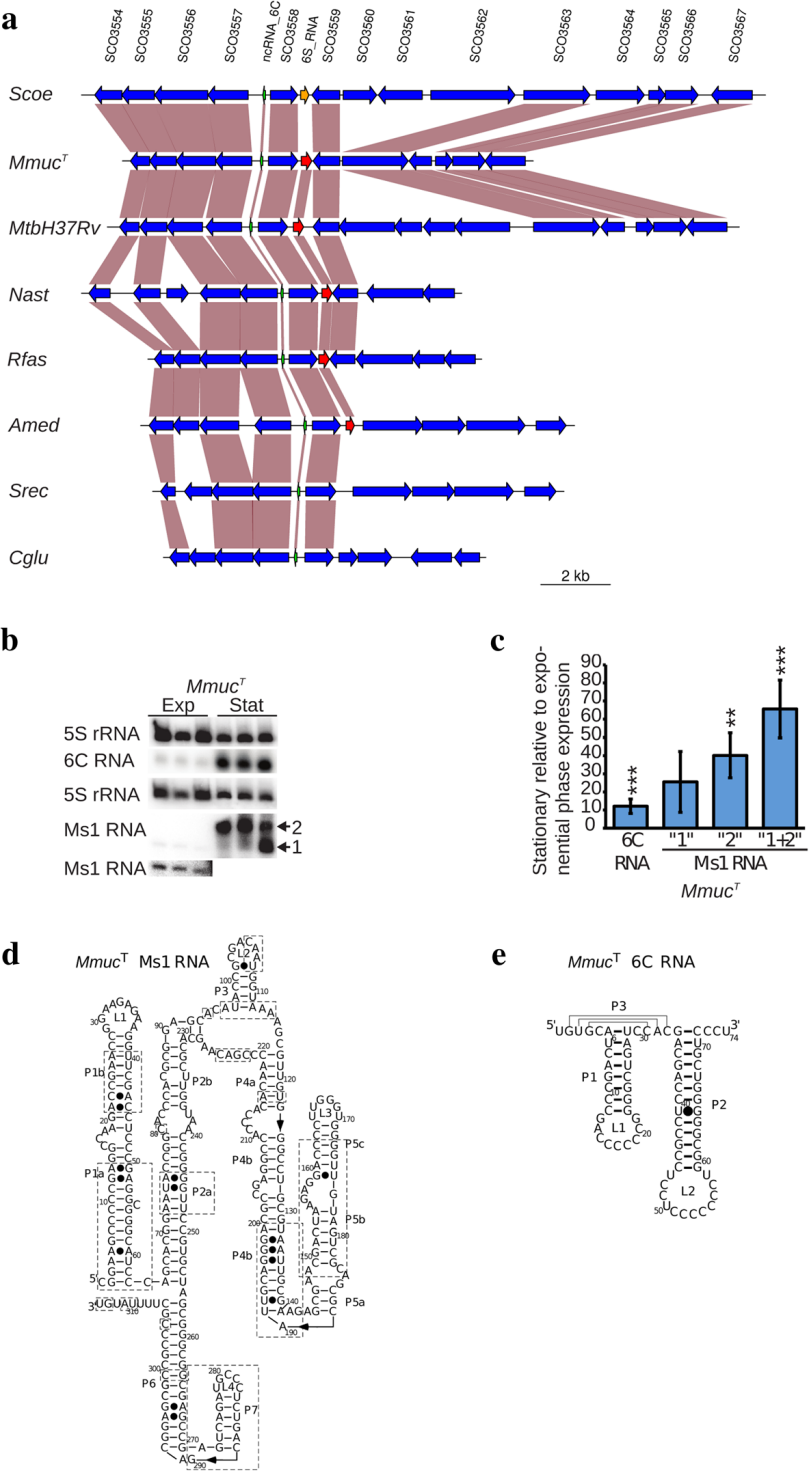
Analysis of the Ms1 and 6C RNA genes. **a** Gene synteny plot for the region encoding Ms1 and 6C RNA genes in selected *Actinomycetes* as indicated. The Ms1 RNA gene is marked in red (orange in *Streptomyces coelicolor*), while the 6C RNA gene is marked in green. The vertical boxes marked in brown highlight homologous genes. **b** Gels showing levels of 5S rRNA, Ms1 RNA and 6C RNA from three independent experiments in stationary and exponentially growing *Mmuc*^T^ and *Maub*^T^ cells as indicated. Note the presence of the extra band, marked as band 1, in one of the experiments. The bottom panel represents overexposure of the Ms1 RNA bands from exponentially growing *Mmuc*^T^ cells. **c** Levels of Ms1 RNA and 6C RNA in stationary cells vs exponentially growing cells in *Mmuc*^T^ as indicated using data presented in (**b**). The experiments were performed as outlined in Methods and values represent the mean and errors based on three independent experiments. Statistical significance using Student’s unpaired, two-tailed t-test; **: *p* < 0.01; ***: *p*< 0.001. **d** Predicted secondary structure for *Mmuc*^T^ Ms1 RNA. The boxes mark regions that vary in sequence comparing *Mmuc*^T^, *Mpho*^T^, *Maub*^T^, *Mneo*^T^ and *Mcos*^T^ (see Additional file 4: Figure S12b). Helices and loops are named P1 to P7 and L1 to L3 as indicated. **e** Predicted secondary structure for *Mmuc*^T^ 6C RNA. The helices and loops are named as in (**d**), see also Additional file 4: Figure S12d

The gene encoding Ms1 RNA was predicted to be present in all members of the *Mmuc*- and *Mneo*-clades as well as in other available mycobacterial and *Actinobacteria* genomes (Fig. 7a; Additional file 4: Figure S12). Comparing gene synteny for the Ms1 RNA region (and sequence alignment) in mycobacteria and *Streptomyces coelicolor* revealed that the Ms1 RNA gene is positioned where the 6S RNA gene maps in *S. coelicolor* [74] (Additional file 4: Figure S12a, b). This supports the notion that Ms1 RNA might be a mycobacterial variant of 6S RNA [75]; see Discussion. A phylogenetic Ms1 DNA tree further suggests that the gene present in *Mneo* strains and *Mmuc*-clade members is close to the mycobacterial ancestral gene (Additional file 4: Figure S12c). We also note that in this tree the *M. chelonae*- and *Mmuc*-clades are positioned close together indicating the evolutionary path of the Ms1 RNA gene.

Northern analysis suggested that Ms1 RNA is expressed in *Mmuc*^T^ and that its level of expression is higher in stationary cells (Fig. 7b, c; Additional file 4: Figure S10; the reason for two bands in one of the samples is unknown but might be related to degradation).

Secondary structural models of the Ms1 RNA were generated based on the *Mmuc*^T^ Ms1 RNA sequence using the M-fold tool/ RNA-fold (Fig. 7d). Comparing the predicted Ms1 RNA structures of *Mmuc*^T^, *Mpho*^T^, *Maub*^T^, *Mneo*^T^ and *Mcos*^T^ revealed nucleotide variations in several regions marked with dashed boxes (Fig. 7d). Several of these changes are consistent with the predicted Ms1 RNA secondary structure. Noteworthy, unconventional base pairing such as G•A base pairs distort the helical structure and these distortions might be part of a protein binding site(s).

Close to the Ms1 RNA gene we identified the presence of the gene encoding 6C RNA, which is widespread among *Actinobacteria* [76, 77]. Its location relative to the Ms1 RNA gene is conserved, with one gene in between, comparing the mycobacteria studied here and *S. coelicolor* (Fig. 7a; Additional file 4: Figure S12a). Sequence alignment also suggested that 6C RNA is highly conserved among mycobacteria belonging to the *Mmuc*- and *Mneo*-clades (Additional file 4: Figure S12d). Although its function in mycobacteria is unclear it is expressed in exponentially growing *Mmuc*^T^ cells and stationary cells with a significantly higher level in stationary cells (Fig. 7b, c; see Discussion).

The 6C RNA secondary structure was manually folded and resulted in a stable structure composed of three helices, P1 to P3, with two C-rich loop structures (Fig. 7e).

### Sar antisense RNA

The ncRNA categorized as Sar antisense RNA was predicted to be unique to *Mmuc*^T^ (and *Mmuc*^CSURP2099^) and located in a phage region (0.65 Mbp region; Fig. 1b; Additional file 4: Figure S8a, b; Table S7). The homology to other Sar RNA genes is low [78]. The *Mmuc*^T^ Sar RNA gene is expressed and the level was modestly higher in stationary cells compared to exponentially growing cells (Fig. 7a, c). In *Salmonella typhimurium* Sar RNA was identified as a regulator during development of the temperate bacteriophage P22 [78]. Its function in *Mmuc*^T^ is unknown. But, the gene is located in the intergenic region between two genes (MUCO_DSM00642 and MUCO_DSM00643) encoding proteins of unknown function where the former encodes a signal peptide, while the Sar RNA gene is transcribed from the opposite strand. It is therefore conceivable that this RNA is involved in regulating the expression of either of these genes or both.

### ncRNAs, comparison with other mycobacteria

We also predicted ncRNA genes in *Mtb*H37Rv and *Msmeg*MC^2^-155 using the Rfam data base (Additional file 4: Figure S8; Table S7; see also [34] and Refs therein). This comparison revealed that 18 (out of 20) ncRNA genes present in the *Mmuc*- and *Mneo*-clades are also present in *Mtb*H37Rv and *Msmeg*MC^2^-155. There are also seven ncRNA genes in *Mtb*H37Rv and *Msmeg*MC^2^-155 for which no orthologs could be found in the 17 genomes (Table 1; Additional file 4: Figure S8). Moreover, the Intron_gpII RNA (Rfam annotation, RF00029), which is present in two copies in *Mtb*H37Rv, is also predicted to be present in *Mpho*^T^ with three copies (Additional file 4: Figure S13a, b). The sequences of the three *Mpho*^T^ gene copies are similar but they differ from the two genes in *Mtb*H37Rv. Comparison of their gene synteny revealed that for *Mtb*H37Rv both Intron_gpII RNA genes are located within genes while for *Mpho*^T^ the three Intron_gpII RNA genes were predicted to be present in intergenic regions (Additional file 4: Figure S13a). Together this suggested that the Intron_gpII RNA ncRNA genes in these two mycobacteria likely are of different origin.

## Discussion

The size of the genomes for *Mmuc*- and *Mneo*-clade members including type strains range between 5.4 to 6.5 Mbp. Core gene phylogeny based on 291 core genes present in 109 mycobacterial genomes and genes common to the *Mmuc*- and *Mneo*-clade members and *M. sp*. URHB0044 positioned the *Mmuc*- and *Mneo*-clades next to one another (Fig. 2a; Additional file 2: Figure S3). The tree based on 2226 genes present in 17 mycobacteria and ANI values cluster the *Mmuc*-clade members (Fig. 2) and indicated that *Mmuc*^LZSF01^ is closer to *Mpho*^T^ than it is to *Mmuc*^T^. Hence, *Mmuc*^LZSF01^ should be considered to be a *Mpho* strain. Consistent with this is also the finding that the numbers of tRNA genes in *Mpho*^T^ and *Mmuc*^LZSF01^ are conserved, as are the structures of 14 tRNA genes encompassing this cluster, while the corresponding cluster in *Mmuc*^T^ lacks three tRNA genes (see below). This analysis also indicates that *Msp*360 and *Msp*410 are likely to be two *Mmuc* strains. Considering the *Mneo*-clade, the positioning of *Mneo*^VKMAc-1815D^ deviates from the other *Mneo* strains including the type strain *Mneo*^T^. This raises the question whether *Mneo*^VKMAc-1815D^ should be considered as a subspecies of *Mneo* or perhaps a different species. For *M. sp*. URHB0044, our data does not provide any information about which clade it belongs to and possibly it is a new mycobacterial species.

### Variation in tRNA among Mmuc- and Mneo-clade members

The number of predicted coding sequences (CDSs) correlates with genome size while the number of tRNA coding genes does not (Table 1). This is also apparent by comparing the number of tRNA genes in the SGM *Mtb*H37Rv (4.4 Mbp; [29]) and RGM *Msmeg*MC^2^-155 (7 Mbp; [30]), which carry 45 and 47 tRNA genes, respectively. Evidently, the number of tRNA genes does not relate to differences in growth rates. Analyzing the tRNA gene organization in the complete *Mmuc*^T^ genome, in total 57 tRNA genes, suggests that the majority of the tRNA genes are transcribed from single genes (32 of 46; not considering the cluster having 11 tRNA genes, which are predicted to be transcribed from two operons, one encompassing eight and the other three genes; Fig. 3). The remaining predicted tRNA operons encode two (four operons) and three (two operons) tRNA genes (Fig. 3a; Additional file 3: Figure S4b-e). This is similar to *Mtb*H37Rv and *Msmeg*MC^2^-155 where 31 of 45 and 33 of 47, respectively, appear to be transcribed from single tRNA genes. The remaining tRNA genes in these two mycobacteria are predicted to be transcribed from operons with two or three tRNA genes and these are the same as those predicted in *Mmuc*^T^ (Additional file 3: Figure S4a, b). In other bacteria, such as the Gram negative *E. coli* K12 and the Gram positive Firmicutes (*e.g*., *B. subtilis* and *Staphylococcus aureus*), the tRNA genes are transcribed from large operons [27, 33, 79–84]. For example, a cluster of as many as 27 tRNA was identified in *S. aureus* [82]. This is in contrast to mycobacteria in which tRNAs in general appear to be transcribed mainly from single genes. This is also supported from our analysis of the tRNA genes in the draft genomes of *Mpho*^T^, *Maub*^T^, *Mneo*^T^ and *Mcos*^T^ (Additional file 3: Figure S4b-e).

Several of the tRNA genes that are common to *Mmuc*^T^, *Mtb*H37Rv and *Msmeg*MC^2^155 (for which complete genomes are available) are positioned at different locations on the circular chromosome suggesting rearrangements of these tRNA genes after these mycobacteria diverged (Additional file 5: Figure S14a, b). However, the positioning of the tRNA genes near the origin of replication, *oriC*, appears to be conserved comparing *Mmuc*^T^, *Mtb*H37Rv and *Msmeg*MC^2^-155 (*oriC*, its location is predicted on the basis of the positioning of *dnaA*, *dnaN* and *rpmH*; [32, 85]; positioning of *oriC* is also supported by the GC skew, see Fig. 1). In this context, certain intergenic regions in *Mtb*H37Rv have been identified as necessary for optimal growth and 11 specific tRNA genes are located in these regions [86]. The same tRNA genes are located at roughly the same positions (with the same orientation) on the *Mmuc*^T^ chromomosome except for the tRNA^Thr^(GGT) and tRNA^Met^(CAT) genes, which have been re-located (Additional file 5: Figure S14a, b). This raises interesting questions with respect to whether it is the tRNA genes and their expression that are needed for optimal growth or if it is their location on the chromosome in relation to *oriC*. Thus, it would be interesting to follow the same protocol as Zhang et al. [86] using other mycobacteria such as *Mmuc*^T^ to address these questions.

### Horizontally transferred tRNA gene clusters

For *Mmuc*^T^ and *Mpho*^T^ (and *Mmuc*^CSURP2099^, *Msp*360 and *Msp*410) we identified tRNA gene clusters carrying 11 and 14 tRNA genes, respectively, while *Maub*^T^ carries a different cluster encoding 32 tRNA genes. On the basis of gene synteny and tRNA gene sequence this latter 32 tRNA gene cluster is likely to be of a different origin than the clusters in *Mmuc*^T^ and *Mpho*^T^ (see also below), which are the same apart from that *Mmuc*^T^ lacks three tRNA genes. Moreover, compared to the commonly present tRNAs the sequences for the genes encompassing these clusters suggest that they encode for structurally different tRNAs. Hence, it is likely that these tRNA gene clusters are the result of horizontal gene transfer and that this occurred after *Mmuc*^T^ and *Mpho*^T^ diverged from *Maub*^T^. This is supported by the presence of homing HNH endonuclease genes within these tRNA gene clusters. Homing endonucleases are known to be involved in moving genes [87, 88] including tRNA genes as in *E. coli* bacteriophages [89]. It is therefore plausible that these large tRNA gene clusters present in *Mmuc*^T^, *Mpho*^T^ and *Maub*^T^ originate from phages.

In the *Mmuc*^T^-*Mpho*^T^ tRNA cluster, the HNH endonuclease gene is positioned between blocks of eight and three genes (six in *Mpho*^T^; Fig. 3b). Among the eight tRNA genes, one encodes tRNA^Ile^(TAT). This tRNA gene is not present in members of the *Mneo*-clade, *Maub*^T^ or *Mtb*H37Rv and *Msmeg*MC^2^-155 and it reads AUA codons. The AUA codon is rare among members of the *Mmuc*- and *Mneo*-clades, while it is more abundant in *Mtb*H37Rv (unpublished data). When the tRNA^Ile^(TAT) gene is missing the AUA codon is read by tRNA^Ile^(CAT) carrying a lysidine (L, 2-lysyl-cytidine) instead of C at position 34 (underlined). The enzyme responsible for this modification is TilS [90] and all the *Mmuc*- and *Mneo*-clade members (and *Mtb*H37Rv) have TilS homologs. The *Mmuc*^T^ tRNA^Ile^(TAT) is expressed and the level is higher in stationary cells. Taken together, for those mycobacteria having the tRNA^Ile^(TAT) gene there appears to be two ways to decode AUA; whether this arrangement gives any advantage remains to be investigated. Also, since the number of tRNA genes in the *Mmuc*^T^-*Mpho*^T^ tRNA cluster differ makes it possible to use this as a marker to differentiate these two species.

In contrast to the *Mmuc*^T^-*Mpho*^T^ tRNA cluster, the *Maub*^T^ gene cluster encompasses both tRNA and protein genes. Large tRNA gene clusters have previously been reported to be present in *M. abscessus* M24 (34 tRNA genes; [91]) and *M. conceptionense* MLE (37 tRNA genes; NCBI bioproject id PRJNA288077; see also [92]). The majority of the tRNA genes encompassing the *Maub*^T^ cluster are also present in the *M. abscessus* M24 and *M. conceptionense* MLE clusters (Additional file 3: Figure S11b; Table S5). In these three mycobacteria, this cluster also encodes GOLLD RNA and HNH endonuclease. This large ncRNA (>400 nucleotides long) was first identified in *Lactobacillus brevis* ATCC 367 [72]. The GOLLD RNA is expressed in *Maub*^T^ albeit at a low level. Its function, however, is not known but it is associated with a prophage and its expression is linked to phage production. Interestingly, in many instances the GOLLD RNA gene is present close to the location of tRNA genes and it also encodes a predicted tRNA gene [72] and for *Maub*^T^ our data suggests that this gene corresponds to a pseudo tRNA gene. Moreover, our phylogenetic analysis indicates that the mycobacterial GOLLD RNA genes likely are of phage origin (unpublished data). Together, this suggests that this large "32 tRNA gene" cluster in *Maub*^T^, which includes the GOLLD RNA gene and a HNH endonuclease gene, is the result of horizontal gene transfer.

The *Maub*^T^ "32 tRNA gene" cluster harbors an additional tRNA^Cys^ gene equipping *Maub*^T^ with three tRNA^Cys^ genes (that are expressed; Fig. 4). Noteworthy, *Mmuc*- and *Mneo*-clade members as well as *Msmeg*MC^2^-155 all have two while *Mtb*H37Rv has one tRNA^Cys^ gene (for other RGM, see [31, 32]; Additional files 3 and 5: Figures S4, S5 and S14). Inspection of their secondary structures reveals that the two extra tRNA^Cys^ both have four base pairs long anticodon stems with the potential to form non-Watson-Crick base pairs, C27-U43 (additional tRNA^Cys^ gene present in all *Mmuc*- and *Mneo*-clade members) and A27-C43 (tRNA^Cys^ gene present only in *Maub*^T^). Whether this influences function or if these extra tRNA^Cys^ isoacceptors indeed are functional or influence growth remains to be investigated.

### RNase P processing and tRNA gene structure

The tRNA genes are transcribed as precursors (pre-tRNAs) and the endoribonuclease P (RNase P) is responsible for generating the tRNA 5' termini. The majority of tRNAs carry a guanosine at their 5' ends, referred to as G_+1_, and seven base pairs long amino acid acceptor (aa-) stems. The residue immediately 5' (the -1 position; N_-1_) of G_+1_ at the RNase P cleavage site in pre-tRNAs has been suggested to interact with the conserved residue A248 (*E. coli* numbering) in the RNA subunit of RNase P (RPR) while G_+1_ is suggested to act as a guiding nucleotide ([35] and Refs therein). The majority (≈60%) of the tRNA genes in *E. coli* have a U at -1, while in mycobacteria the occurrence of U at this position is below 20%. Interestingly, G at -1 is frequent in mycobacteria and can be as high as 30% (Additional file 5: Figure S15). These observations lead to two important points. First, mycobacteria have A at the corresponding "248 position" in the RPR. It is therefore unlikely that the majority of tRNA precursors form a Watson-Crick base pair between residue -1 and "A248" in mycobacterial RPRs ([35] and Refs therein). Second, since G at the cleavage site functions as a guiding nucleotide the question is how mycobacterial RNase P handle the processing of those tRNA transcripts with G at -1. The tRNA^His^ transcript carries G at -1 and is cleaved at -1 instead of +1 generating a functional tRNA with an eight base pair long aa-stem ([35] and Refs therein). For the other mycobacterial tRNA transcripts carrying G at -1, RNase P might first cleave at -1 and then at the correct site, +1, generating functional tRNAs with seven base pairs long aa-stems. Another possibility, mycobacterial RNase P has an intrinsic capacity to efficiently cleave tRNA transcripts at the correct site +1 despite the presence of G at -1. In this context, we note that the frequency of G_-1_ for the "HGT tRNA genes" in *Mmuc*^T^, *Mpho*^T^ and *Maub*^T^ discussed above is low (or absent), while for U_-1_ it is ≈50% (Additional file 5: Figure S15). This further supports the notion that these tRNA genes have been acquired through horizontal gene transfer.

### Stable ncRNAs – RPR, tmRNA, 4.5S RNA, Ms1 RNA ("6S RNA") and 6C RNA

A number of functional ncRNAs have been identified in bacteria and recently several reports document the presence of significant numbers of ncRNAs in *Mtb*H37Rv and *Msmeg*MC^2^-155 using both *in silico* and experimental approaches (reviewed by [34, 93]). Here we identified several putative ncRNAs in *Mmuc*- and *Mneo*-clade members. Focusing on the "classical" ncRNAs such as the catalytic RNase P RNA, the scavenging tmRNA and the signal recognition particle 4.5S RNA we showed that their levels in *Mmuc*^T^ (and tmRNA in *Maub*^T^) are higher in stationary phase compared to exponential phase. This is in keeping with their levels of expression being influenced by growth conditions (see *e.g*. [94–96]). Interestingly, tmRNA is involved in the control of the *Caulobacter* cell-cycle, sporulation in *Bacillus* and cell development in *Streptomyces* species [96]. Given that mycobacteria undergo changes in their cell morphology, including spore formation, upon exposure to environmental changes [97–99] it would be interesting to study the impact of tmRNA on cell morphology among mycobacteria.

The 6S RNA is a global transcriptional regulator in bacteria with a role in the adaptation to stationary phase. It binds to the housekeeping σ factor (σ^A^) in the RNA polymerase holoenzyme and thereby preventing the polymerase from binding to DNA promoters that require σ^A^ for transcription. The identification of 6S RNA in mycobacteria has been an enigma [100]. We predicted the presence of genes homologous to the Ms1 RNA gene in all *Mmuc*- and *Mneo*-clade members. In other bacteria the level of 6S RNA increases when the cells enter stationary phase [100]. Our data showed that the level of Ms1 RNA in *Mmuc*^T^ cells is significantly higher in stationary phase compared to exponential phase in keeping with what has been reported for *Mtb*H37Rv and *Msmeg*MC^2^-155 [75, 101]. Moreover, Hnilicová et al. [75] provided data suggesting that Ms1 RNA binds to the RNA polymerase core enzyme and not to any of the σ factors, including σ^A^. Searching for the presence of the Ms1 RNA gene among the *Actinobacteria* reveals that homologs are present in several species (Fig. 7a; Additional file 4: Figure S12). The gene synteny for 6S RNA in *S. coelicolor* [74, 102] and the Ms1 RNA gene in mycobacteria are similar. Together this suggests that Ms1 RNA might be a 6S RNA variant that interacts with RNA polymerase and thereby affect the expression of genes; however, whether it has the same function as 6S RNA in other bacteria remains to be deciphered (see [75]). In this context, the level of Ms1 RNA accumulates upon infection of mice lungs. Also, overexpression of Ms1 RNA in *Mtb*H37Rv affects the levels of roughly 300 genes [93, 101]. Hence, like 6S RNA, Ms1 RNA appears to be a global regulator with a role in adaptation to stationary phase and growth inside the host cells.

We also identified another known and conserved gene close to the Ms1 RNA gene in the *Mmuc*- and *Mneo*-clade members, the 6C RNA gene for which we have little functional information. Its location is conserved relative to the Ms1 RNA gene among different *Actinobacteria* and the level of 6C RNA is higher in *Mmuc*^T^ cells in stationary phase compared to exponentially growing cells, albeit not as pronounced as for Ms1 RNA. The expression of 6C RNA has also been detected in other mycobacteria, *Cornynebacterium glutamicum* and *S. coelicolor* [76, 77, 103, 104]. In *S. coelicolor*, 6C RNA is upregulated during development/sporulation and it has been discussed that it reflects dormancy and slower metabolism [76]. For *C. glutamicum*, it is suggested to be involved in the SOS response and possibly also in the control of cell division as well as the GlxR, a regulator of carbon source metabolism and energy conversion, regulatory network [77, 105]. The role of mycobacterial 6C RNA is unknown but from the discussion above it is conceivable that it is involved in regulating cell shape and development including dormancy and spore formation in mycobacteria [97, 98], for a review see [99]. Clearly, deciphering the functions of these (and other) ncRNAs will provide information about the way mycobacteria adapts to different growth conditions and perhaps also in relation to cell differentiation.

## Conclusions

The genome sizes for *Mmuc*- and *Mneo*-clade members range between 5.4 to 6.5 Mbp and phages, IS elements, horizontally transferred tRNA gene clusters, and phage-derived ncRNAs have likely influenced the evolution of the *Mmuc*- and *Mneo*-clades. While the number of predicted coding sequences correlates with genome size, the number of tRNA coding genes does not. Among the *Mmuc*- and *Mneo*-clade members, 39 tRNA genes are common. Members of the *Mmuc*-clade harbor several tRNA genes that have been acquired through HGT. The majority of the tRNA genes in mycobacteria are transcribed from single genes. As in other mycobacteria tRNA charging of asparagine and glutamine is accommodated using the tRNA-dependent amidotransferase pathway (Adt) in the *Mmuc*- and *Mneo*-clade members. The levels of RNase P RNA (essential for the processing of tRNAs), tmRNA, and 4.5S RNA are higher at stationary phase compared to exponentially growing cells. Two new and horizontally transferred ncRNAs are present and expressed in *Mmuc*-clade members. The levels of the ncRNAs, 6C RNA and Ms1 RNA, are higher in cells at stationary phase compared to exponentially growing cells. The Ms1 RNA likely represents a mycobacterial 6S RNA variant. The evolutionary routes for the ncRNAs RNase P RNA, tmRNA and Ms1 RNA are different from that of the core genes.

## Additional files

Additional file 1: **Introduction.** Table and Figure legends. **Table S1.** Compilation of oligonucleotide probes used in the present study. **Table S2.** Summary of genome assembly. **Table S3.** Phage analysis of Mmuc- and Mneo-clade members. **Table S4a.** Annotation of IS elements in MmucT. **Table S4b.** Annotation of IS elements in Mmuc- and Mneo-clade members. **Figure S1.** Genome alignments. (ZIP 1120 kb)

Additional file 2: **Introduction.** Figure legends. **Figure S2a, b.** Functional classification of genes. **Figure S3a, b.** Phylogenetic analysis. (ZIP 151 kb)

Additional file 3: **Introduction.** Table and Figure legends. **Table S5.** Compilation of predicted tRNA genes in the "32 tRNA gene cluster". **Table S6a.** Compilation of predicted aminoacyl-tRNA synthetases (AARS) paralogs. **Table S6b.** Compilation of predicted genes encoding GatCAB enzymes. **Table S6c.** Compilation of regular and extra gene copy aminoacyl-tRNA synthetase genes. Supplementary text information. Prediction of genes encoding aminoacyl-tRNA synthetase paralogs and cyclodipeptide synthetase genes in Mmuc- and Mneo-clade members. **Figure S4a-e.** Analysis of tRNA genes [90]. **Figure S5.** tRNA sequence alignment for all tRNA genes. **Figure S6a-f.** Analysis of isoleucyl-tRNA synthetase and selected AARS genes. **Figure S7a, b.** Cyclodipeptide synthase (CDPS) – PF16715 [106, 107]. (ZIP 166 kb)

Additional file 4: **Introduction.** Table and Figure legends. **Table S7.** Rfam non-coding RNA annotations. **Figure S8a, b.** ncRNA genes. **Figure S9a-i.** RNase P RNA (rnpB), tmRNA and 4.5S RNA. **Figure S10.** Northern blot analysis of selected tRNAs and ncRNAs with size markers. **Figure S11a, b.** Analysis of GOLLD RNA. **Figure S12a-d.** Comparison of Ms1 RNA and 6C RNA genes in mycobacteria. **Figure S13a, b.** Comparison of a intron Group II gene cluster. (ZIP 7530 kb)

Additional file 5: **Introduction.** Figure legends. **Figure S14a, b.** Comparing positioning of tRNA genes in MmucT, MtbH37Rv and MsmegMC2-155. **Figure S15a, b.** Frequency of the identity of the nucleobase at position -1 in tRNA genes [35]. (ZIP 3300 kb)

## Data Availability

All data and materials are available and adheres to BMC Evolutionary Biology policies on sharing data and materials. Genome sequences have been deposited at NCBI, nucleotide sequence accession numbers: POTL00000000: *Mycobacterium mucogenicum* DSM 44124 POTM00000000: *Mycobacterium phocaicum* DSM 45104 POTN00000000: *Mycobacterium aubagnense* DSM 45150 POTO00000000: *Mycobacterium neoaurum* DSM 44074 POTP00000000: *Mycobacterium cosmeticum* DSM 44829

## Abbreviations

AARS: Aminoacyl-tRNA Synthetase
Adt: Amidotransferase pathway
ANI: Average Nucleotide Identity
ArgRS: Arginyl-tRNA synthetase
Asn: Asparagine
AsnRS: Asparaginyl-tRNA synthetase
CDPS: Cyclodipeptide synthetase
CDS: Coding sequence
DSM: Deutsche Sammlung von Mikroorganismen
GlnRS: Glutaminyl-tRNA synthetase
GluRS: Glutamyl-tRNA synthetase
HGT: Horizontal Gene Transfer
His: Histidine
HNH: The approximately 35 amino acids long peptide carry two conserved histidines and one asparagine (HNH)
IleRS: Isoleucyl-tRNA-synthetase
IS: Insertion sequence
*M. sp*. URHB0044: *Mycobacterium species* URHB0044
*Maub*: *Mycobacterium aubagnense*
Mbps, kbp and bp: Million base pairs, thousand base pairs and base pair, respectively
*Mche*: *Mycobacterium chelonae*
*Mcos*: *Mycobacterium cosmeticum*
*Mllat*: *Mycobacterium llaterzerense* strain CLUC14
*Mmuc*: *Mycobacterium mucogenicum*
*Mmuc*^CSURP2099^: *Mycobacterium mucogenicum* strain CSURP2099
*Mmuc*^LZLC01^: *Mycobacterium mucogenicum* strain LZLC01
*Mmuc*^LZSF01^: *Mycobacterium mucogenicum* strain LZSF01
*Mneo*: *Mycobacterium neoaurum*
*Mneo*^VKMAc-1815D^: *Mycobacterium neoaurum* strain VKMAc-1815D
*Mpho*: *Mycobacterium phocaicum*
*Msmeg*: *Mycobacterium smegmatis*
*Msp*280: *M. sp*. UNC280 or *Mycobacterium species* UNC280
*Msp*360: *M. sp*. 360MFT or *Mycobacterium species* 360MFT
*Msp*410: *M. sp*. UNC410 or *Mycobacterium species* UNC410
*Mtb*: *Mycobacterium tuberculosis*
ncRNA: Non-coding RNA
ProRS: Prolyl-tRNA synthetase
Rfam: Database for ncRNA families and structured RNA elements
RPR: Ribonuclease P RNA
rRNA (rDNA): Ribosomal RNA (DNA)
T (as superscript): Type strain
tmRNA: Transfer-messenger RNA
