# Supplementary material for: Comparative genomics of Mycobacterium mucogenicum and Mycobacterium neoaurum clade members emphasizing tRNA and non-coding RNA
Source: BMC Evol Biol. 2019 Jun 18;19:124. doi: 10.1186/s12862-019-1447-7 (PMC6582537; doi:10.1186/s12862-019-1447-7)
Supplement: Supplementary file 1 — Introduction. Table and Figure legends. Table S1. Compilation of oligonucleotide probes used in the present study. Table S2. Summary of genome assembly. Table S3. Phage analysis of Mmuc- and Mneo-clade members. Table S4a. Annotation of IS elements in MmucT. Table S4b. Annotation of IS elements in Mmuc- and Mneo-clade members. Figure S1. Genome alignments. (ZIP 1120 kb) [file 12862_2019_1447_MOESM1_ESM.zip › 12862_2019_1447_MOESM1_ESM/ADDITIONAL FILE 1 INTRODUCTION .pdf]

Additional file 1: Introduction. Table and Figure legends, Additional file 1.

Table S1. Compilation of oligonucleotide probes used in the present study.

Table S2. Summary of genome assembly.

Table S3. Phage analysis of *Mmuc*- and *Mneo*-clade members.

Table S4a. Annotation of IS elements in *Mmuc*^{T}.

Table S4b. Annotation of IS elements in *Mmuc*- and *Mneo*-clade members.

Figure S1. Genome alignments.

(a) Whole-genome alignment for 10 members of the *Mmuc*-clade including the type strains *Mmuc*^{T}, *Mpho*^{T} and *Maub*^{T}. Connecting lines between the genomes mark homologous regions while diagonal lines indicate genome rearrangements: blue lines, genomic inversions and white gaps, deletions/ insertions, see also figure legend 1b. Noteworthy, the blue diagonal lines between *Mmuc*^{T} and *Mmuc*^{CSURP2099}, and *Mmuc*^{CSURP2099} and *Mmuc*^{LZLC01} does not indicate genomic rearrangement rather it is related to scaffolding and assembly of the *Mmuc*^{CSURP2099} and *Mmuc*^{LZLC01} genomes. For both *Mmuc*^{CSURP2099} and *Mmuc*^{LZLC01} *dnaA* was positioned at the center of the scaffold.

(b) Whole-genome alignment for four *Mneo* strains as indicated and described above.

(c) Whole-genome alignment for the two available *Mcos*^{T} genomes, one sequenced in this study and the other available at the NCBI database (ftp://ftp.ncbi.nlm.nih.gov/genomes/).
