# Supplementary figures and images for: Comparative genomics of Mycobacterium mucogenicum and Mycobacterium neoaurum clade members emphasizing tRNA and non-coding RNA
Source: BMC Evol Biol. 2019 Jun 18;19:124. doi: 10.1186/s12862-019-1447-7 (PMC6582537; doi:10.1186/s12862-019-1447-7)

Fig S1a

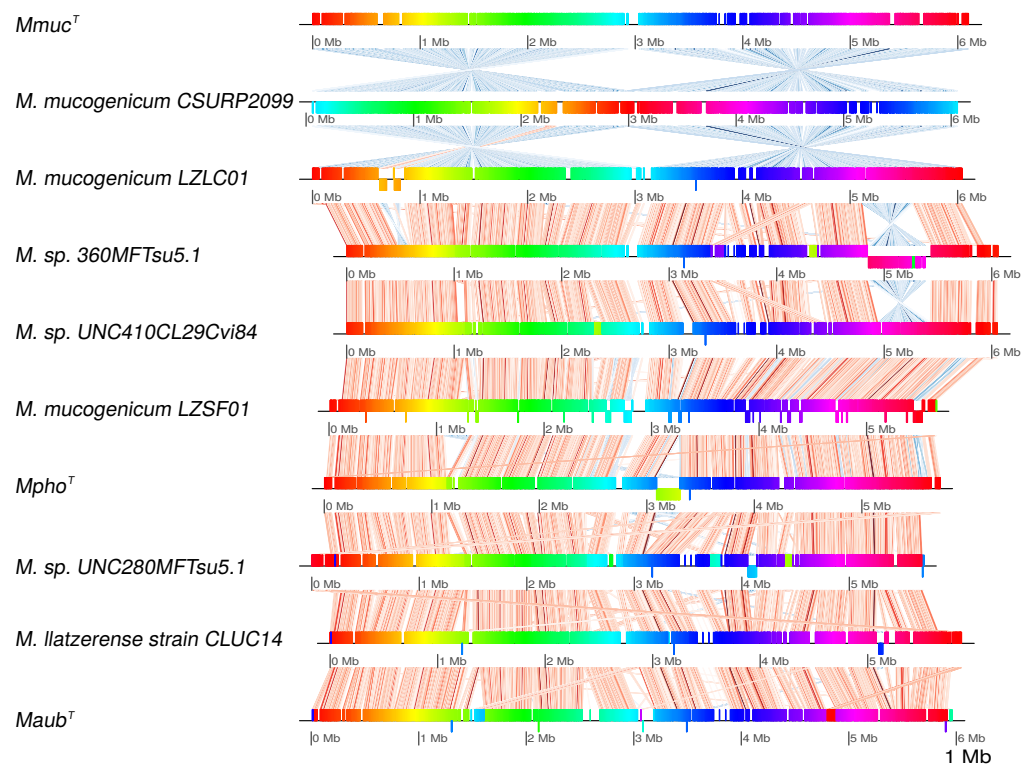

Fig S1b

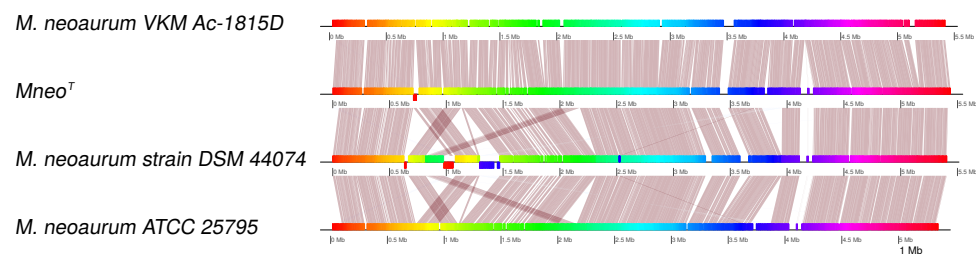

Fig S1c

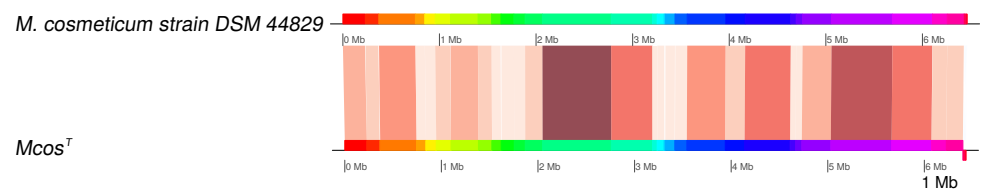

Supplement: Supplementary file 1 — Introduction. Table and Figure legends. Table S1. Compilation of oligonucleotide probes used in the present study. Table S2. Summary of genome assembly. Table S3. Phage analysis of Mmuc- and Mneo-clade members. Table S4a. Annotation of IS elements in MmucT. Table S4b. Annotation of IS elements in Mmuc- and Mneo-clade members. Figure S1. Genome alignments. (ZIP 1120 kb) [file 12862_2019_1447_MOESM1_ESM.zip › 12862_2019_1447_MOESM1_ESM/Fig S1a-c.pdf]

Fig S2a

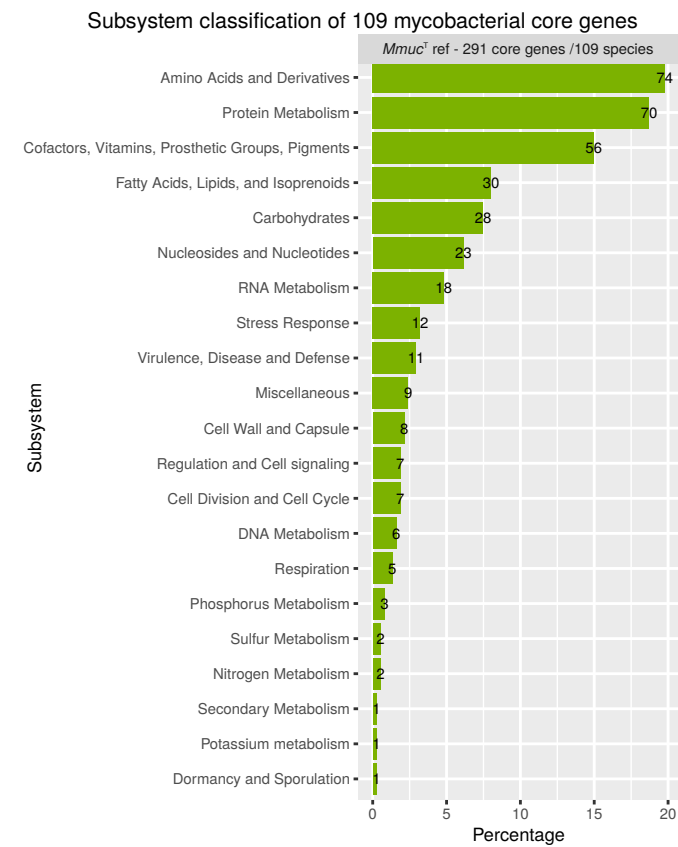

Fig S2b

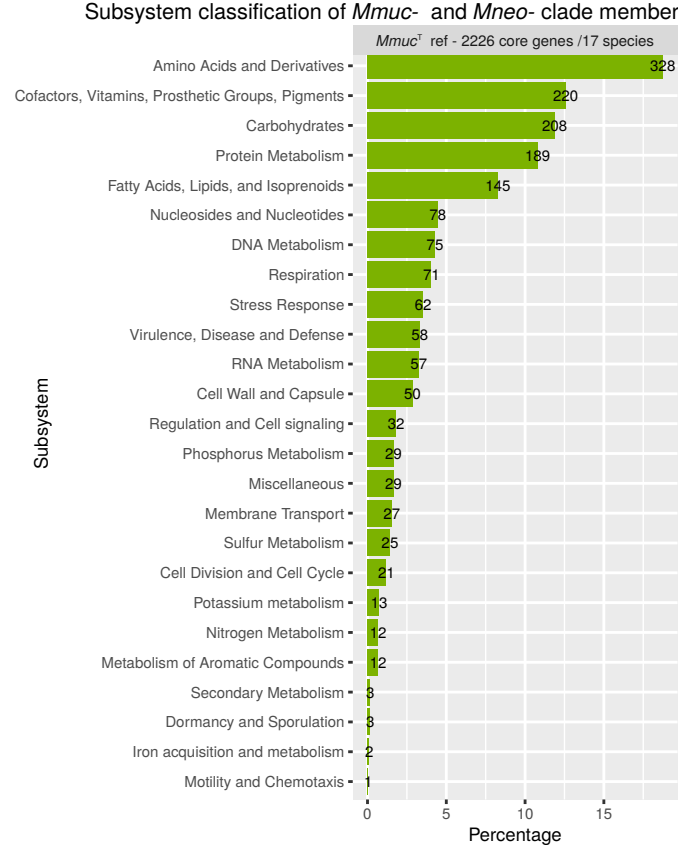

Supplement: Supplementary file 2 — Introduction. Figure legends. Figure S2a, b. Functional classification of genes. Figure S3a, b. Phylogenetic analysis. (ZIP 151 kb) [file 12862_2019_1447_MOESM2_ESM.zip › 12862_2019_1447_MOESM2_ESM/Fig S2ab.pdf]

Fig S6f

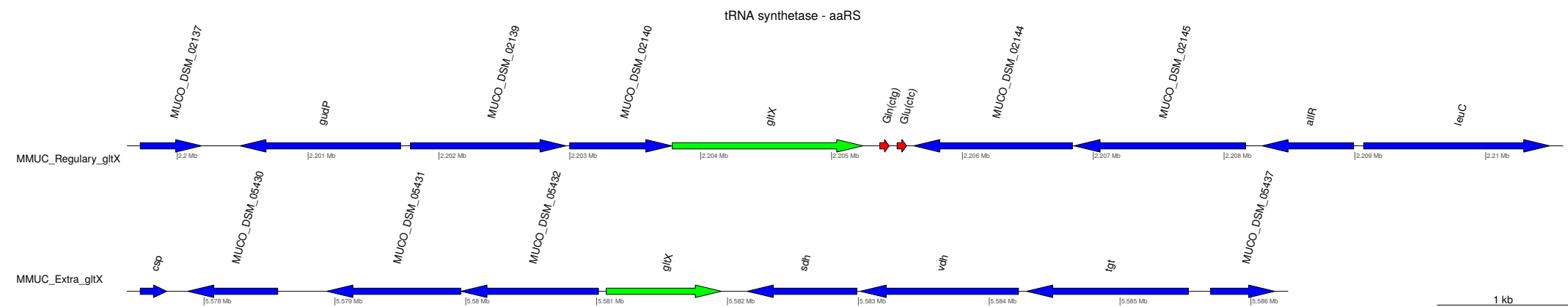

Supplement: Supplementary file 3 — Introduction. Table and Figure legends. Table S5. Compilation of predicted tRNA genes in the "32 tRNA gene cluster". Table S6a. Compilation of predicted aminoacyl-tRNA synthetases (AARS) paralogs. Table S6b. Compilation of predicted genes encoding GatCAB enzymes. Table S6c. Compilation of regular and extra gene copy aminoacyl-tRNA synthetase genes. Supplementary text information. Prediction of genes encoding aminoacyl-tRNA synthetase paralogs and cyclodipeptide synthetase genes in Mmuc- and Mneo-clade members. Figure S4a-e. Analysis of tRNA genes [90]. Figure S5. tRNA sequence alignment for all tRNA genes. Figure S6a-f. Analysis of isoleucyl-tRNA synthetase and selected AARS genes. Figure S7a, b. Cyclodipeptide synthase (CDPS) – PF16715 [106, 107]. (ZIP 166 kb) [file 12862_2019_1447_MOESM3_ESM.zip › 12862_2019_1447_MOESM3_ESM/Fig S6f.pdf]

Fig S6d

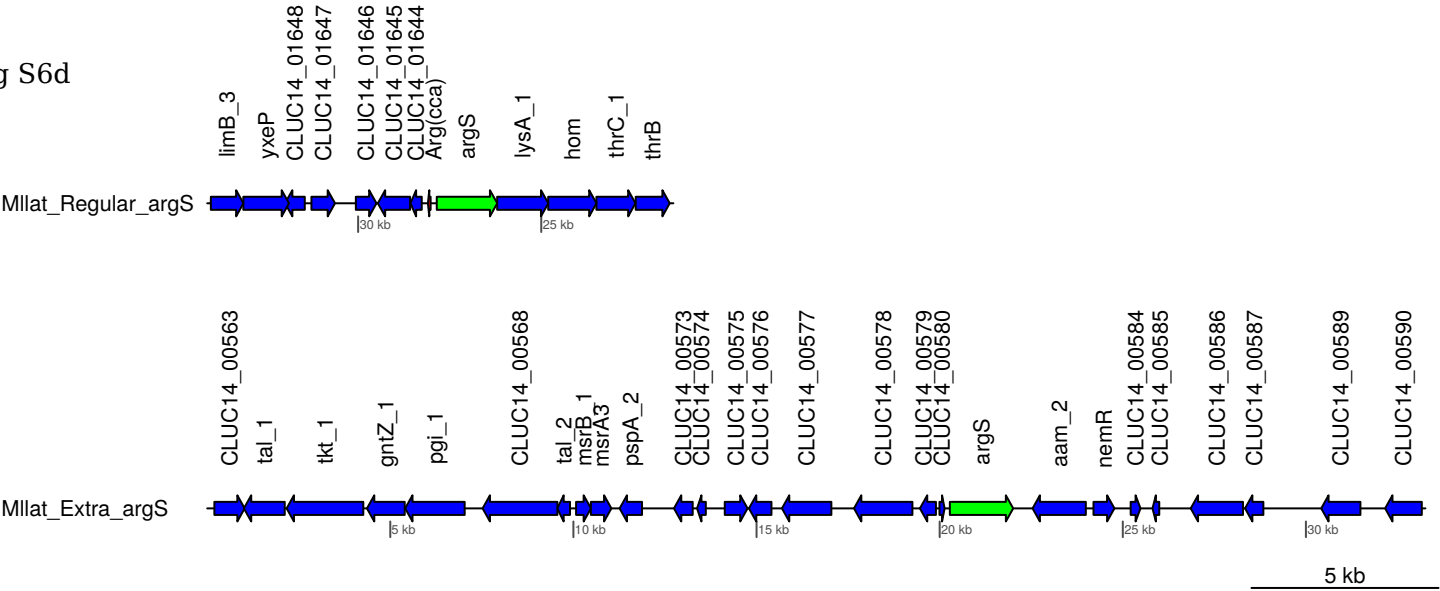

Fig S6e

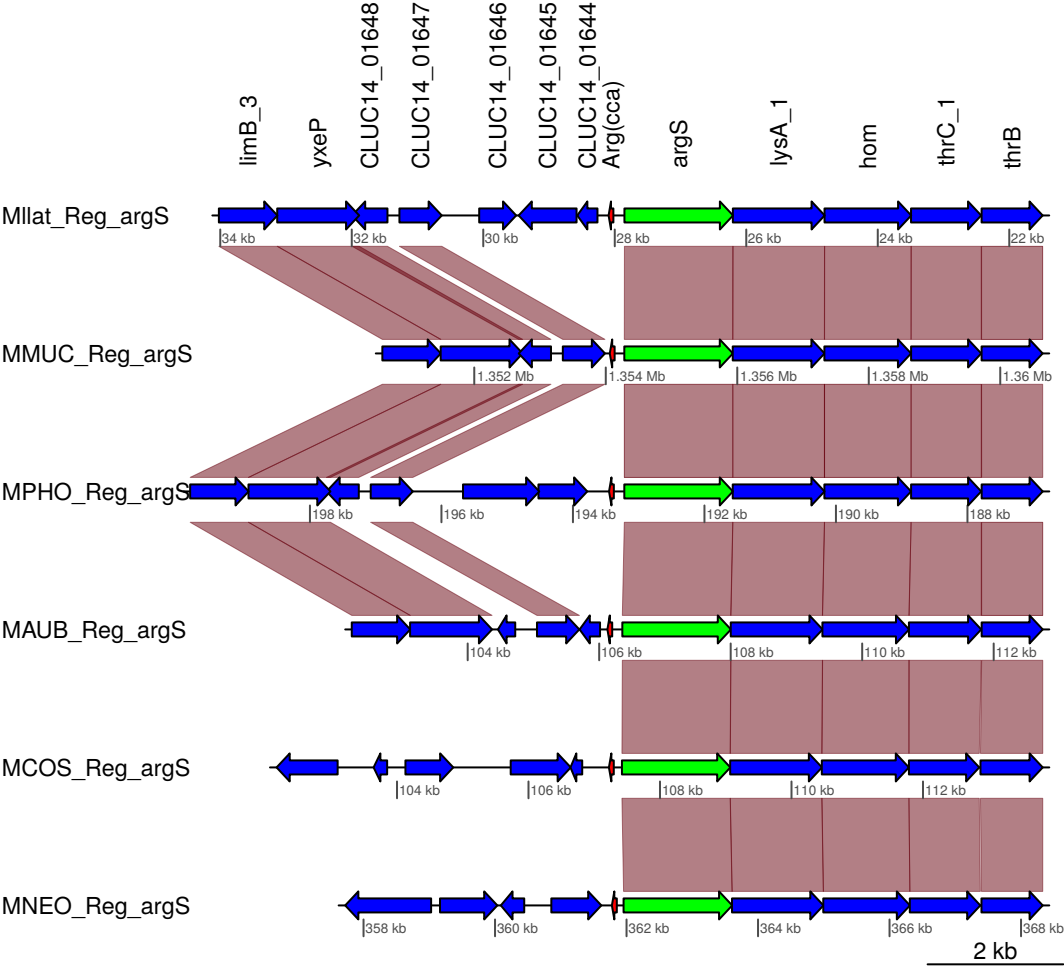

Supplement: Supplementary file 3 — Introduction. Table and Figure legends. Table S5. Compilation of predicted tRNA genes in the "32 tRNA gene cluster". Table S6a. Compilation of predicted aminoacyl-tRNA synthetases (AARS) paralogs. Table S6b. Compilation of predicted genes encoding GatCAB enzymes. Table S6c. Compilation of regular and extra gene copy aminoacyl-tRNA synthetase genes. Supplementary text information. Prediction of genes encoding aminoacyl-tRNA synthetase paralogs and cyclodipeptide synthetase genes in Mmuc- and Mneo-clade members. Figure S4a-e. Analysis of tRNA genes [90]. Figure S5. tRNA sequence alignment for all tRNA genes. Figure S6a-f. Analysis of isoleucyl-tRNA synthetase and selected AARS genes. Figure S7a, b. Cyclodipeptide synthase (CDPS) – PF16715 [106, 107]. (ZIP 166 kb) [file 12862_2019_1447_MOESM3_ESM.zip › 12862_2019_1447_MOESM3_ESM/Fig S6de.pdf]

Fig S6b

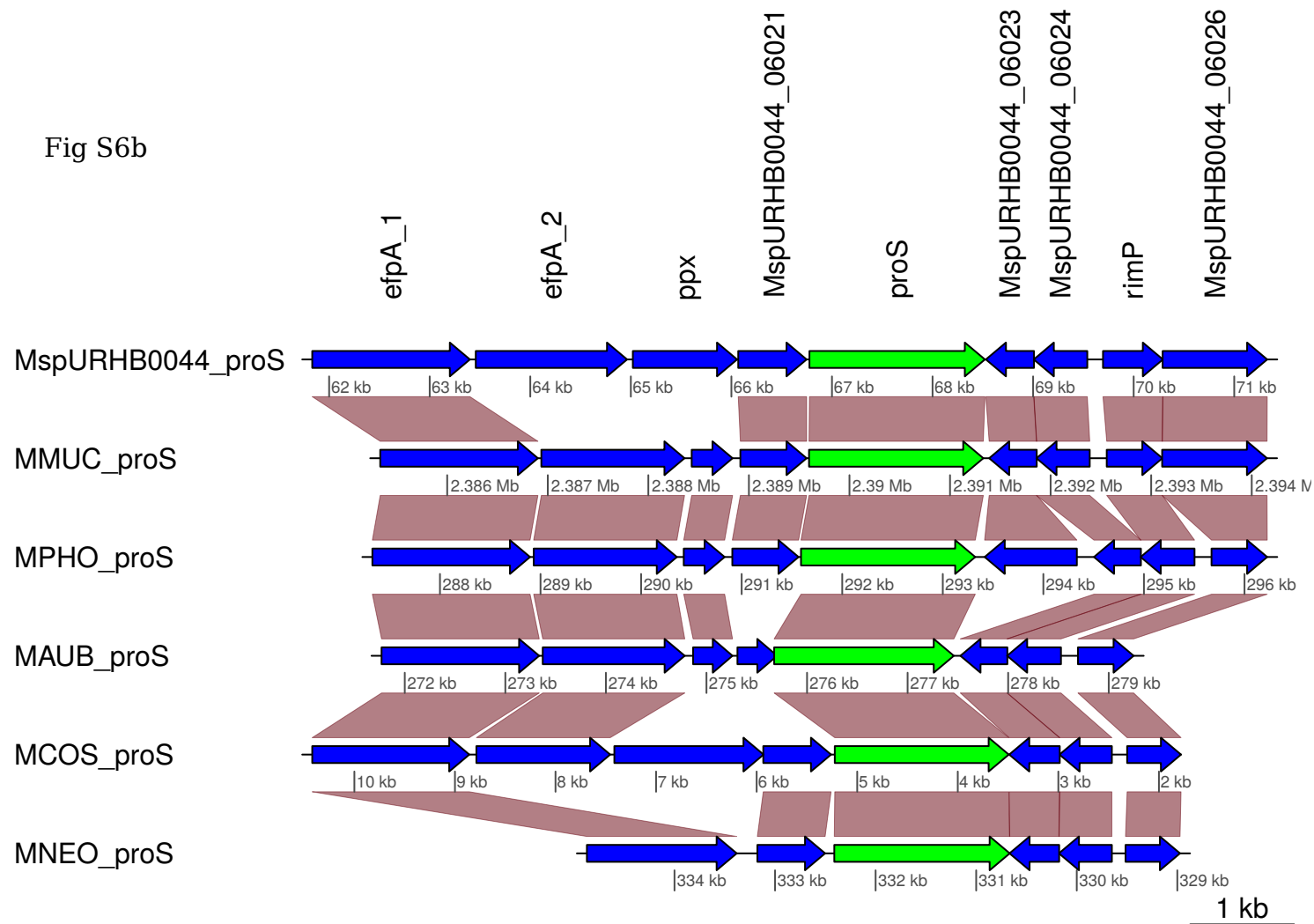

Fig S6c

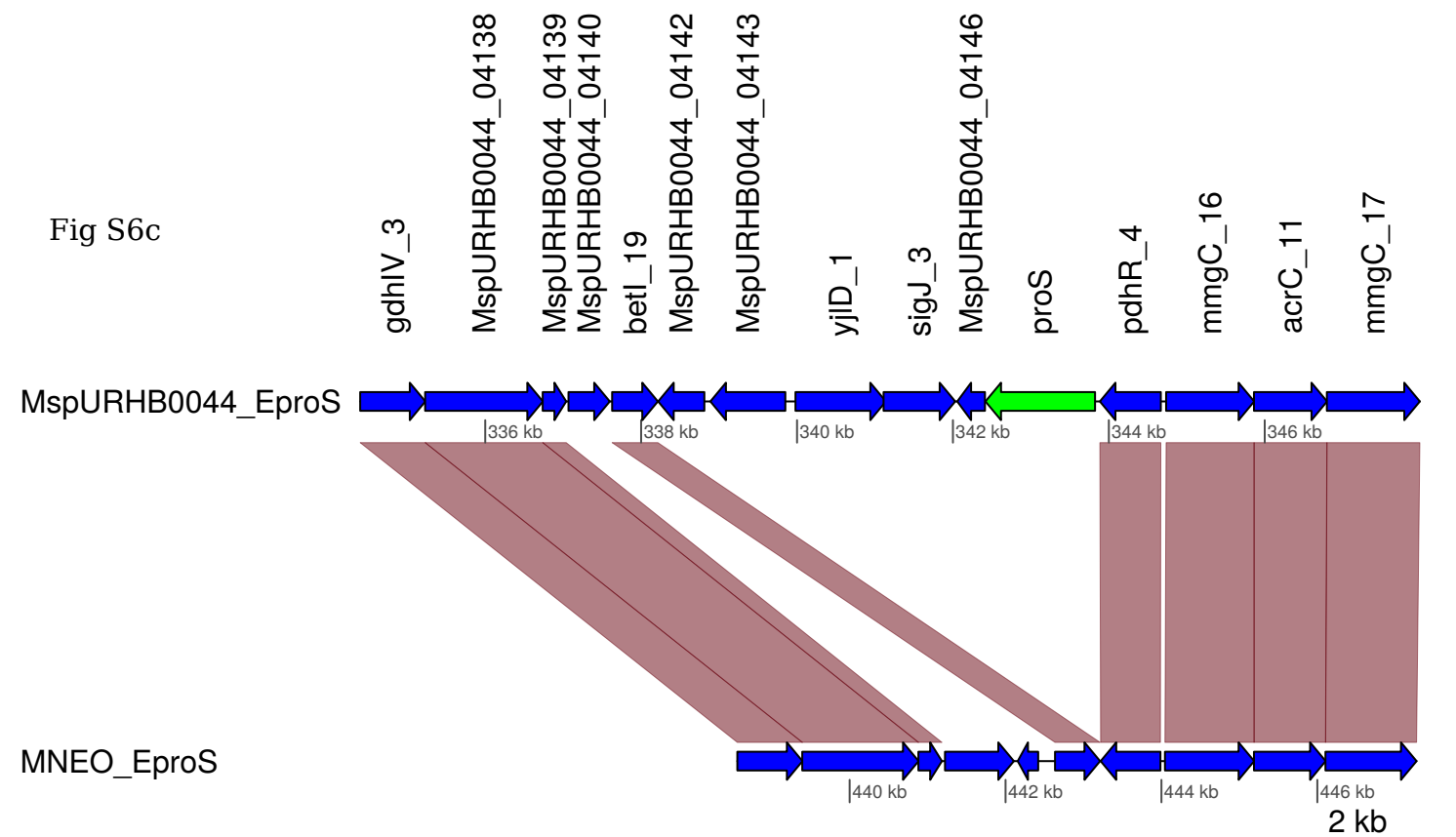

Supplement: Supplementary file 3 — Introduction. Table and Figure legends. Table S5. Compilation of predicted tRNA genes in the "32 tRNA gene cluster". Table S6a. Compilation of predicted aminoacyl-tRNA synthetases (AARS) paralogs. Table S6b. Compilation of predicted genes encoding GatCAB enzymes. Table S6c. Compilation of regular and extra gene copy aminoacyl-tRNA synthetase genes. Supplementary text information. Prediction of genes encoding aminoacyl-tRNA synthetase paralogs and cyclodipeptide synthetase genes in Mmuc- and Mneo-clade members. Figure S4a-e. Analysis of tRNA genes [90]. Figure S5. tRNA sequence alignment for all tRNA genes. Figure S6a-f. Analysis of isoleucyl-tRNA synthetase and selected AARS genes. Figure S7a, b. Cyclodipeptide synthase (CDPS) – PF16715 [106, 107]. (ZIP 166 kb) [file 12862_2019_1447_MOESM3_ESM.zip › 12862_2019_1447_MOESM3_ESM/Fig S6bc.pdf]

Fig S4e

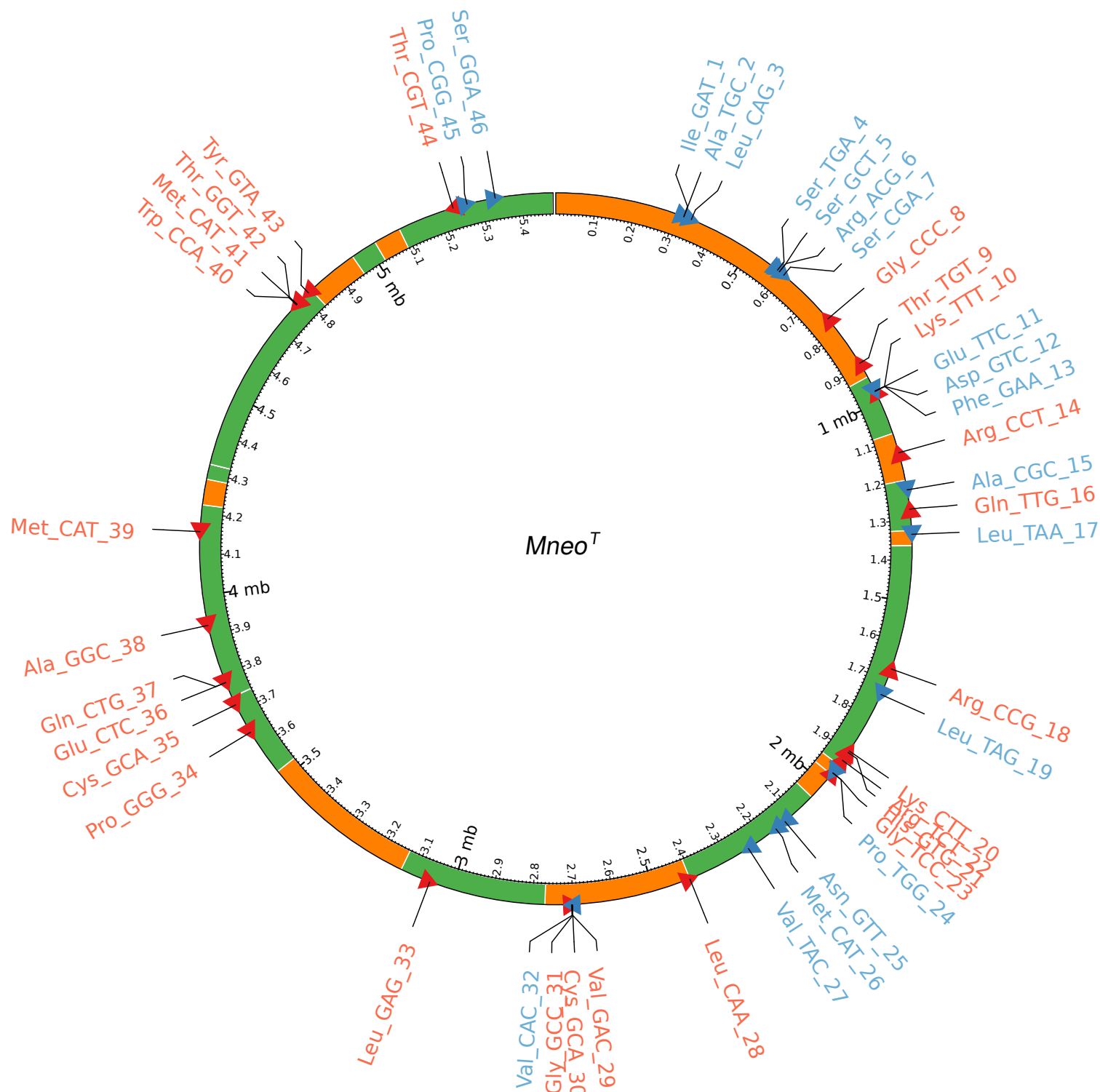

Supplement: Supplementary file 3 — Introduction. Table and Figure legends. Table S5. Compilation of predicted tRNA genes in the "32 tRNA gene cluster". Table S6a. Compilation of predicted aminoacyl-tRNA synthetases (AARS) paralogs. Table S6b. Compilation of predicted genes encoding GatCAB enzymes. Table S6c. Compilation of regular and extra gene copy aminoacyl-tRNA synthetase genes. Supplementary text information. Prediction of genes encoding aminoacyl-tRNA synthetase paralogs and cyclodipeptide synthetase genes in Mmuc- and Mneo-clade members. Figure S4a-e. Analysis of tRNA genes [90]. Figure S5. tRNA sequence alignment for all tRNA genes. Figure S6a-f. Analysis of isoleucyl-tRNA synthetase and selected AARS genes. Figure S7a, b. Cyclodipeptide synthase (CDPS) – PF16715 [106, 107]. (ZIP 166 kb) [file 12862_2019_1447_MOESM3_ESM.zip › 12862_2019_1447_MOESM3_ESM/Fig S4e.pdf]

Fig S4d

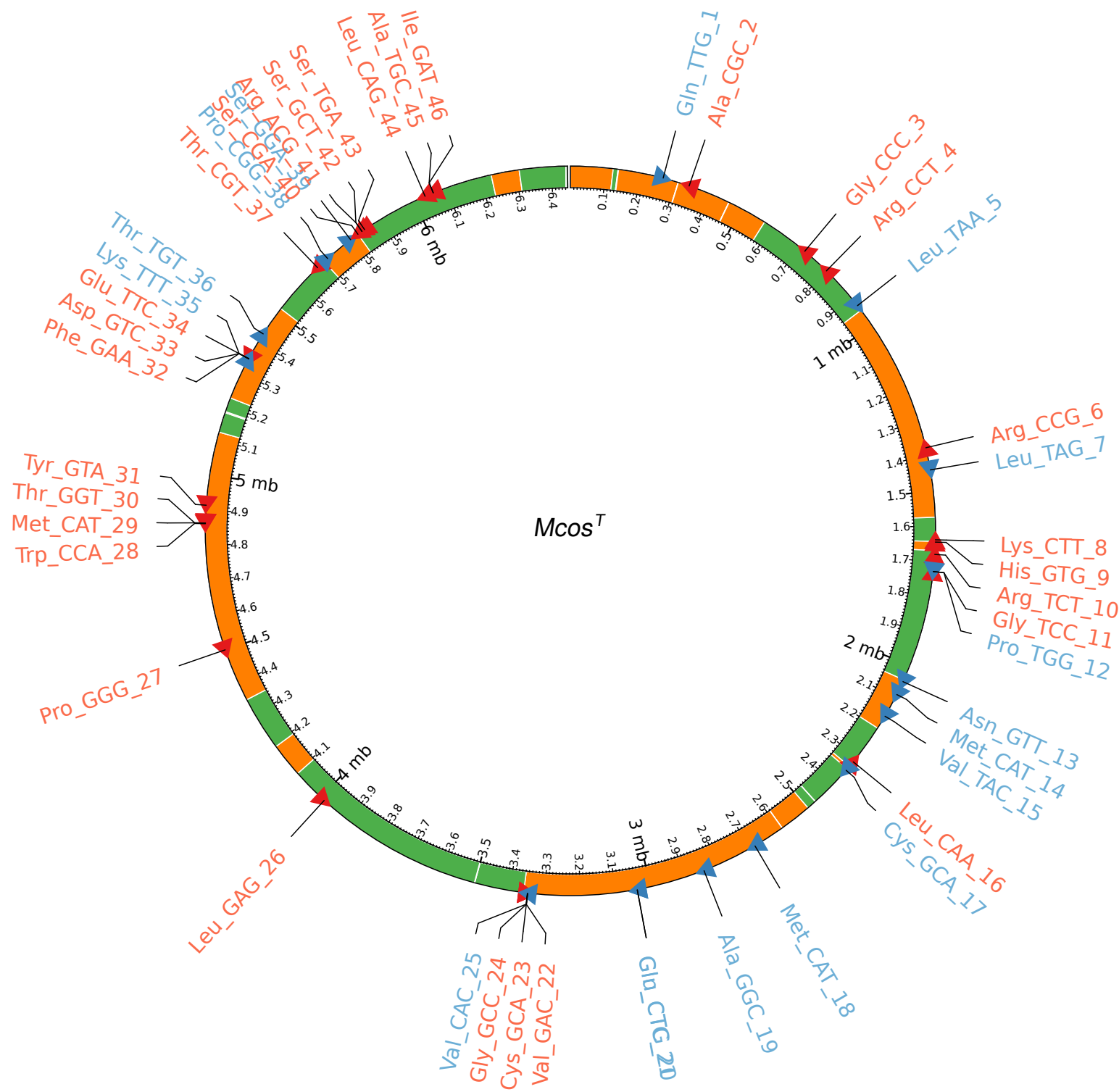

Supplement: Supplementary file 3 — Introduction. Table and Figure legends. Table S5. Compilation of predicted tRNA genes in the "32 tRNA gene cluster". Table S6a. Compilation of predicted aminoacyl-tRNA synthetases (AARS) paralogs. Table S6b. Compilation of predicted genes encoding GatCAB enzymes. Table S6c. Compilation of regular and extra gene copy aminoacyl-tRNA synthetase genes. Supplementary text information. Prediction of genes encoding aminoacyl-tRNA synthetase paralogs and cyclodipeptide synthetase genes in Mmuc- and Mneo-clade members. Figure S4a-e. Analysis of tRNA genes [90]. Figure S5. tRNA sequence alignment for all tRNA genes. Figure S6a-f. Analysis of isoleucyl-tRNA synthetase and selected AARS genes. Figure S7a, b. Cyclodipeptide synthase (CDPS) – PF16715 [106, 107]. (ZIP 166 kb) [file 12862_2019_1447_MOESM3_ESM.zip › 12862_2019_1447_MOESM3_ESM/Fig S4d.pdf]

Fig S4c

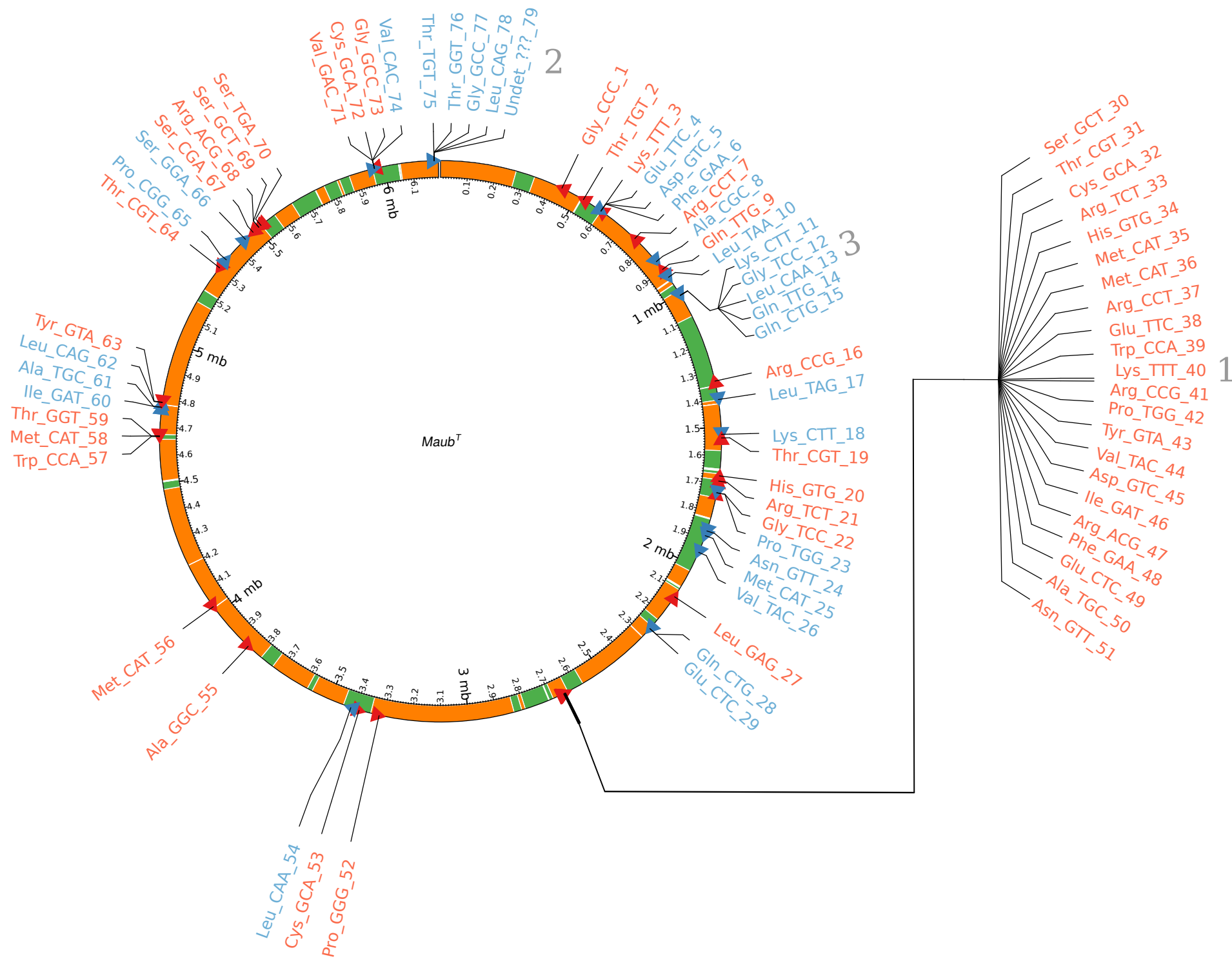

Supplement: Supplementary file 3 — Introduction. Table and Figure legends. Table S5. Compilation of predicted tRNA genes in the "32 tRNA gene cluster". Table S6a. Compilation of predicted aminoacyl-tRNA synthetases (AARS) paralogs. Table S6b. Compilation of predicted genes encoding GatCAB enzymes. Table S6c. Compilation of regular and extra gene copy aminoacyl-tRNA synthetase genes. Supplementary text information. Prediction of genes encoding aminoacyl-tRNA synthetase paralogs and cyclodipeptide synthetase genes in Mmuc- and Mneo-clade members. Figure S4a-e. Analysis of tRNA genes [90]. Figure S5. tRNA sequence alignment for all tRNA genes. Figure S6a-f. Analysis of isoleucyl-tRNA synthetase and selected AARS genes. Figure S7a, b. Cyclodipeptide synthase (CDPS) – PF16715 [106, 107]. (ZIP 166 kb) [file 12862_2019_1447_MOESM3_ESM.zip › 12862_2019_1447_MOESM3_ESM/Fig S4c.pdf]

Fig S4b

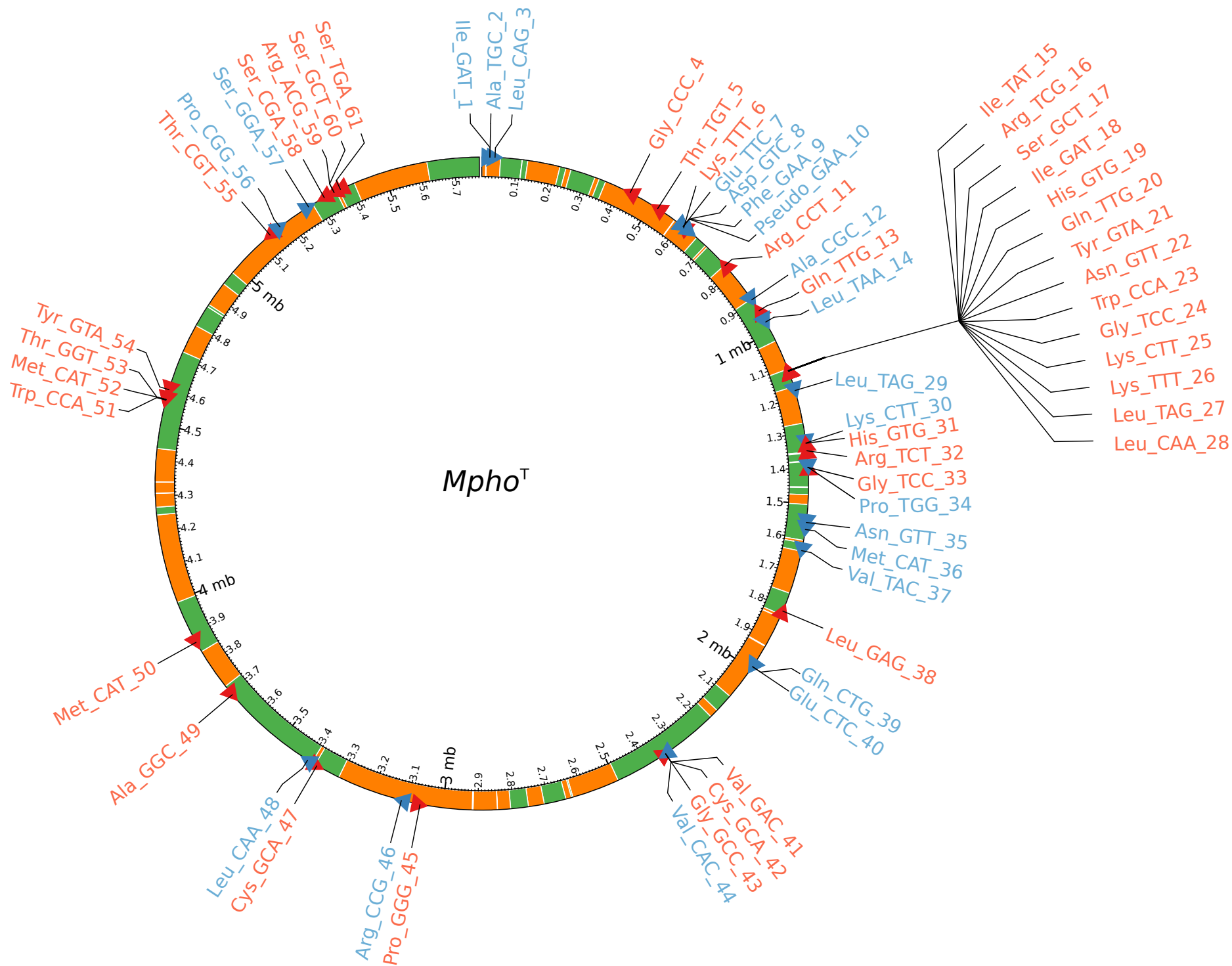

Supplement: Supplementary file 3 — Introduction. Table and Figure legends. Table S5. Compilation of predicted tRNA genes in the "32 tRNA gene cluster". Table S6a. Compilation of predicted aminoacyl-tRNA synthetases (AARS) paralogs. Table S6b. Compilation of predicted genes encoding GatCAB enzymes. Table S6c. Compilation of regular and extra gene copy aminoacyl-tRNA synthetase genes. Supplementary text information. Prediction of genes encoding aminoacyl-tRNA synthetase paralogs and cyclodipeptide synthetase genes in Mmuc- and Mneo-clade members. Figure S4a-e. Analysis of tRNA genes [90]. Figure S5. tRNA sequence alignment for all tRNA genes. Figure S6a-f. Analysis of isoleucyl-tRNA synthetase and selected AARS genes. Figure S7a, b. Cyclodipeptide synthase (CDPS) – PF16715 [106, 107]. (ZIP 166 kb) [file 12862_2019_1447_MOESM3_ESM.zip › 12862_2019_1447_MOESM3_ESM/Fig S4b.pdf]

Fig S4a

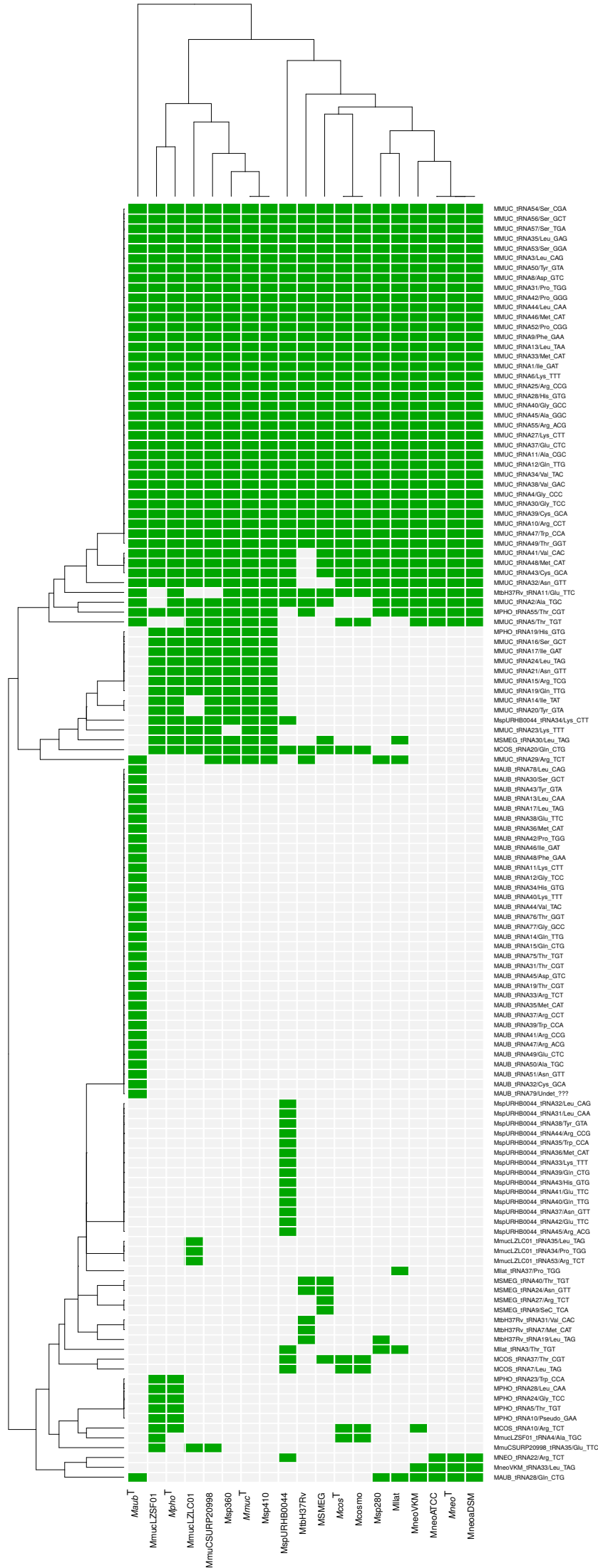

Supplement: Supplementary file 3 — Introduction. Table and Figure legends. Table S5. Compilation of predicted tRNA genes in the "32 tRNA gene cluster". Table S6a. Compilation of predicted aminoacyl-tRNA synthetases (AARS) paralogs. Table S6b. Compilation of predicted genes encoding GatCAB enzymes. Table S6c. Compilation of regular and extra gene copy aminoacyl-tRNA synthetase genes. Supplementary text information. Prediction of genes encoding aminoacyl-tRNA synthetase paralogs and cyclodipeptide synthetase genes in Mmuc- and Mneo-clade members. Figure S4a-e. Analysis of tRNA genes [90]. Figure S5. tRNA sequence alignment for all tRNA genes. Figure S6a-f. Analysis of isoleucyl-tRNA synthetase and selected AARS genes. Figure S7a, b. Cyclodipeptide synthase (CDPS) – PF16715 [106, 107]. (ZIP 166 kb) [file 12862_2019_1447_MOESM3_ESM.zip › 12862_2019_1447_MOESM3_ESM/Fig S4a.pdf]

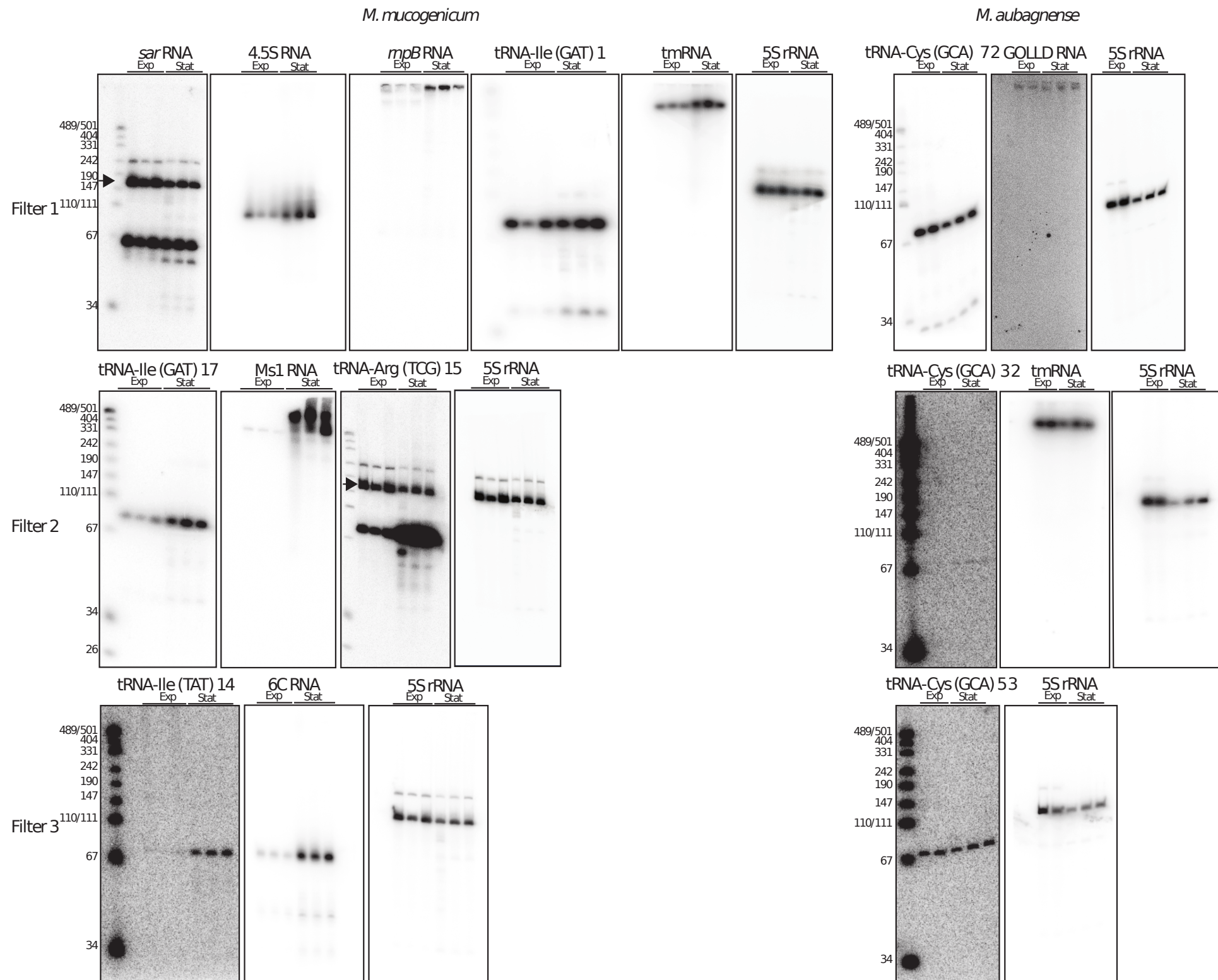

Supplement: Supplementary file 4 — Introduction. Table and Figure legends. Table S7. Rfam non-coding RNA annotations. Figure S8a, b. ncRNA genes. Figure S9a-i. RNase P RNA (rnpB), tmRNA and 4.5S RNA. Figure S10. Northern blot analysis of selected tRNAs and ncRNAs with size markers. Figure S11a, b. Analysis of GOLLD RNA. Figure S12a-d. Comparison of Ms1 RNA and 6C RNA genes in mycobacteria. Figure S13a, b. Comparison of a intron Group II gene cluster. (ZIP 7530 kb) [file 12862_2019_1447_MOESM4_ESM.zip › 12862_2019_1447_MOESM4_ESM/Fig S10.pdf]

Fig S13a

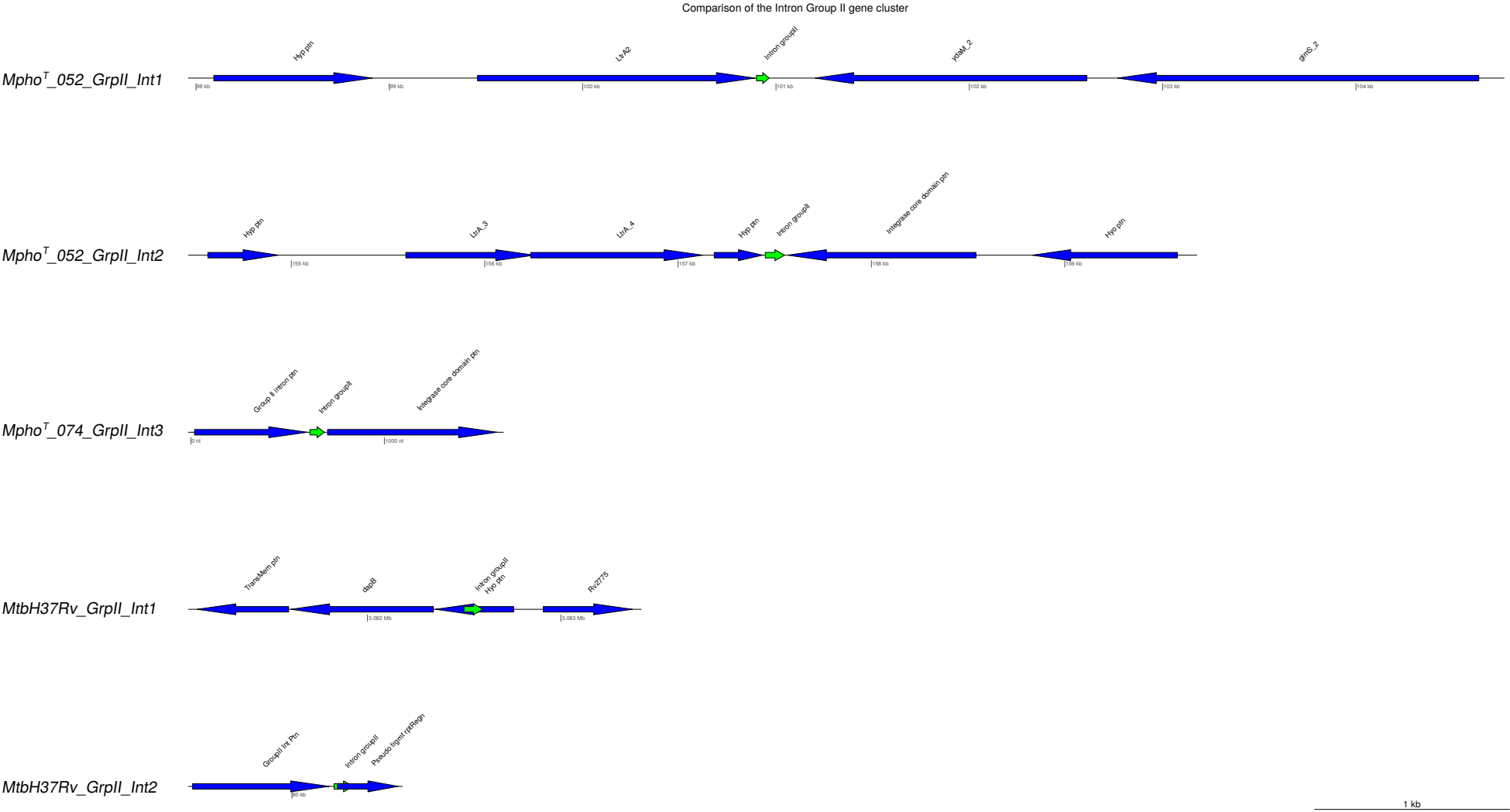

Fig S13b

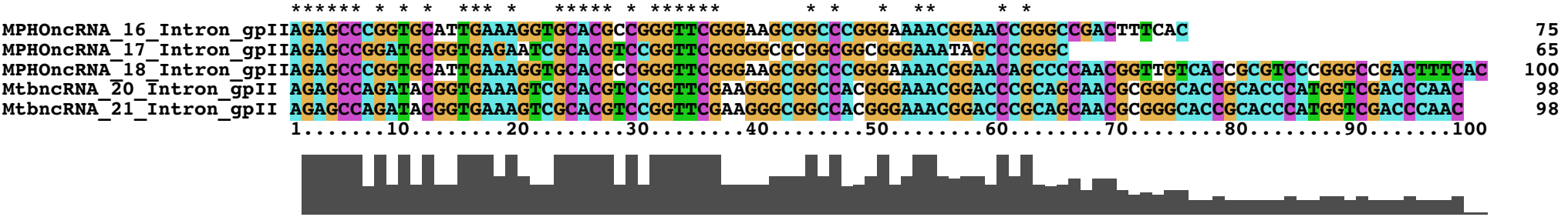

Supplement: Supplementary file 4 — Introduction. Table and Figure legends. Table S7. Rfam non-coding RNA annotations. Figure S8a, b. ncRNA genes. Figure S9a-i. RNase P RNA (rnpB), tmRNA and 4.5S RNA. Figure S10. Northern blot analysis of selected tRNAs and ncRNAs with size markers. Figure S11a, b. Analysis of GOLLD RNA. Figure S12a-d. Comparison of Ms1 RNA and 6C RNA genes in mycobacteria. Figure S13a, b. Comparison of a intron Group II gene cluster. (ZIP 7530 kb) [file 12862_2019_1447_MOESM4_ESM.zip › 12862_2019_1447_MOESM4_ESM/Fig S13ab.pdf]

Fig S8a

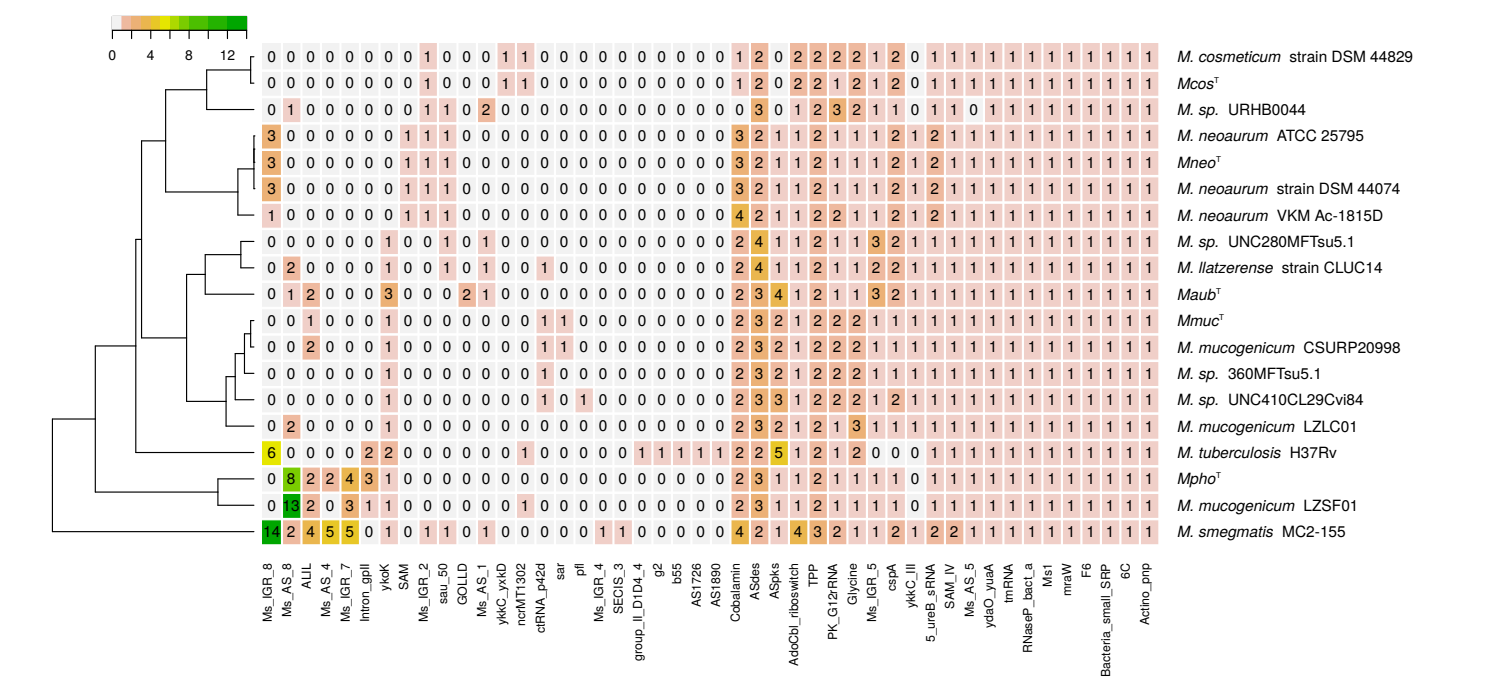

Fig S8b

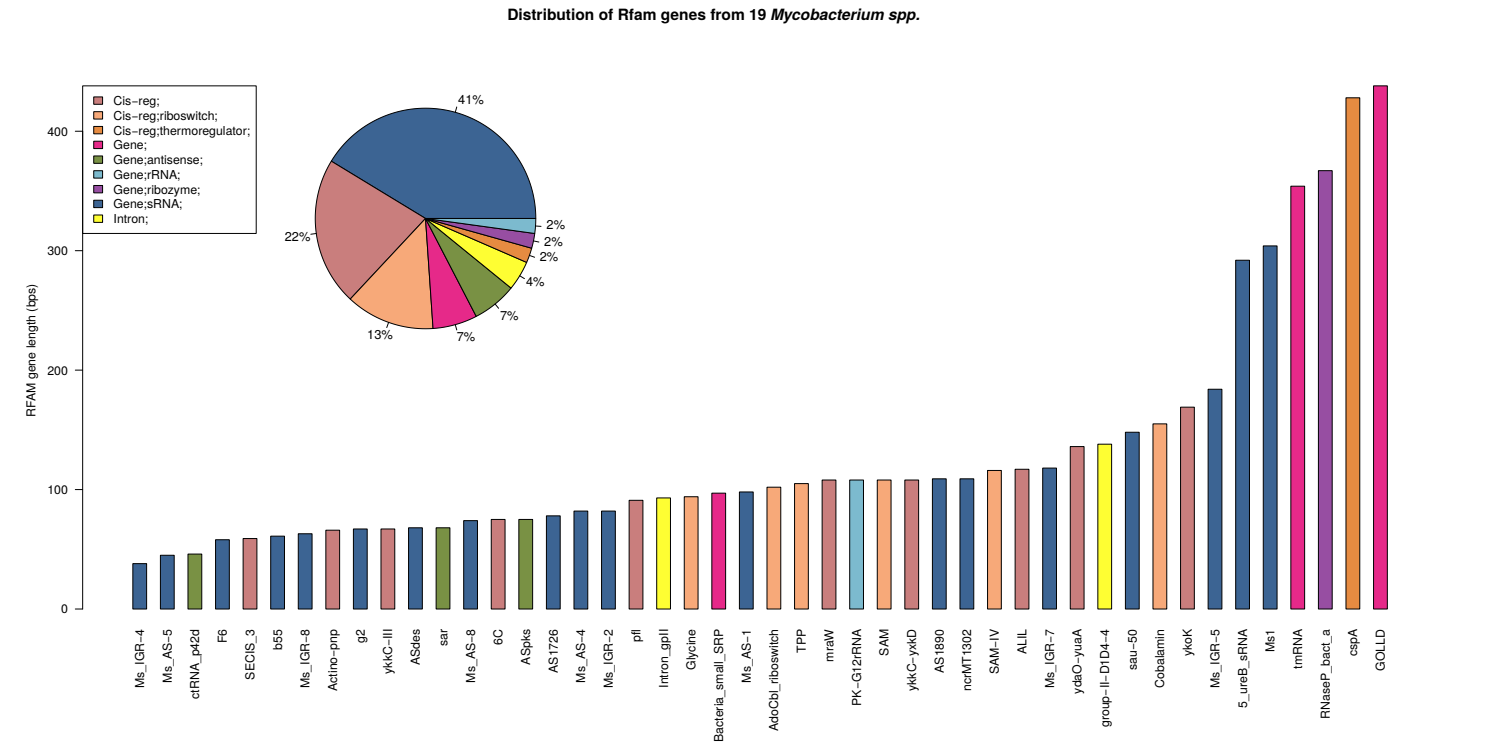

Supplement: Supplementary file 4 — Introduction. Table and Figure legends. Table S7. Rfam non-coding RNA annotations. Figure S8a, b. ncRNA genes. Figure S9a-i. RNase P RNA (rnpB), tmRNA and 4.5S RNA. Figure S10. Northern blot analysis of selected tRNAs and ncRNAs with size markers. Figure S11a, b. Analysis of GOLLD RNA. Figure S12a-d. Comparison of Ms1 RNA and 6C RNA genes in mycobacteria. Figure S13a, b. Comparison of a intron Group II gene cluster. (ZIP 7530 kb) [file 12862_2019_1447_MOESM4_ESM.zip › 12862_2019_1447_MOESM4_ESM/Fig S8ab.pdf]

Fig S15a

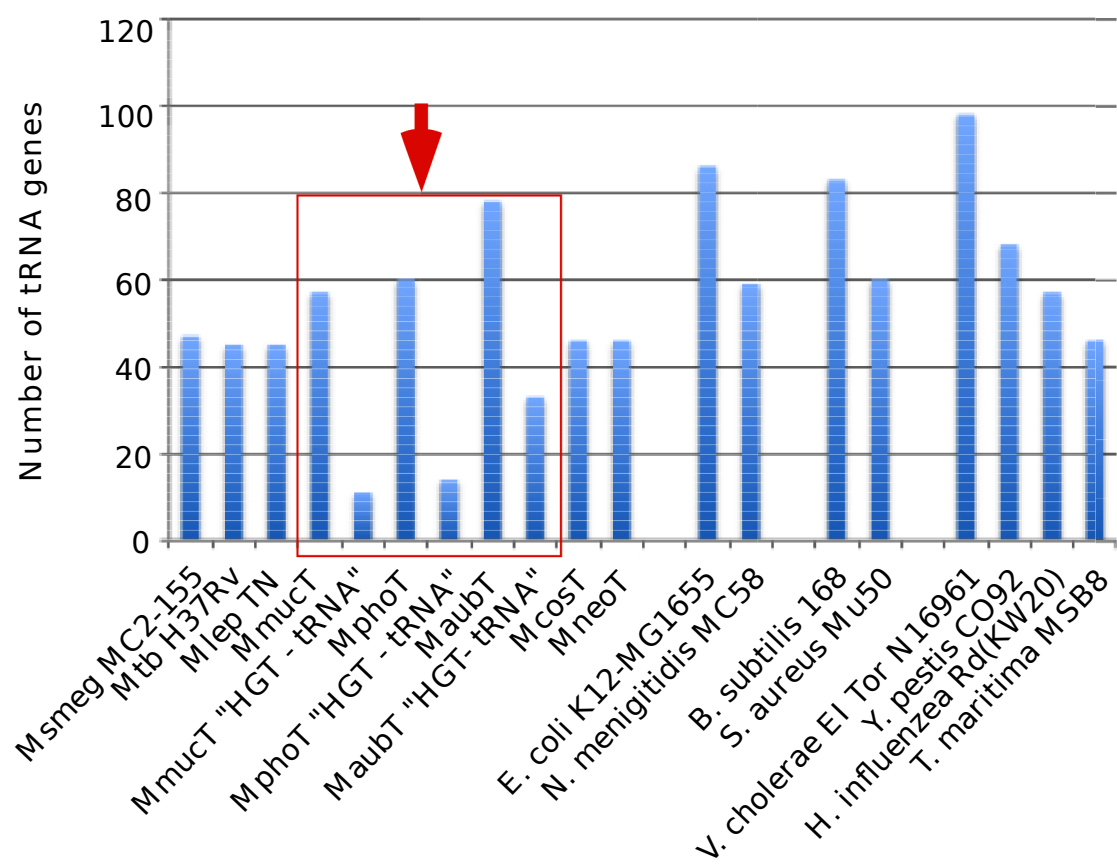

Fig S15b

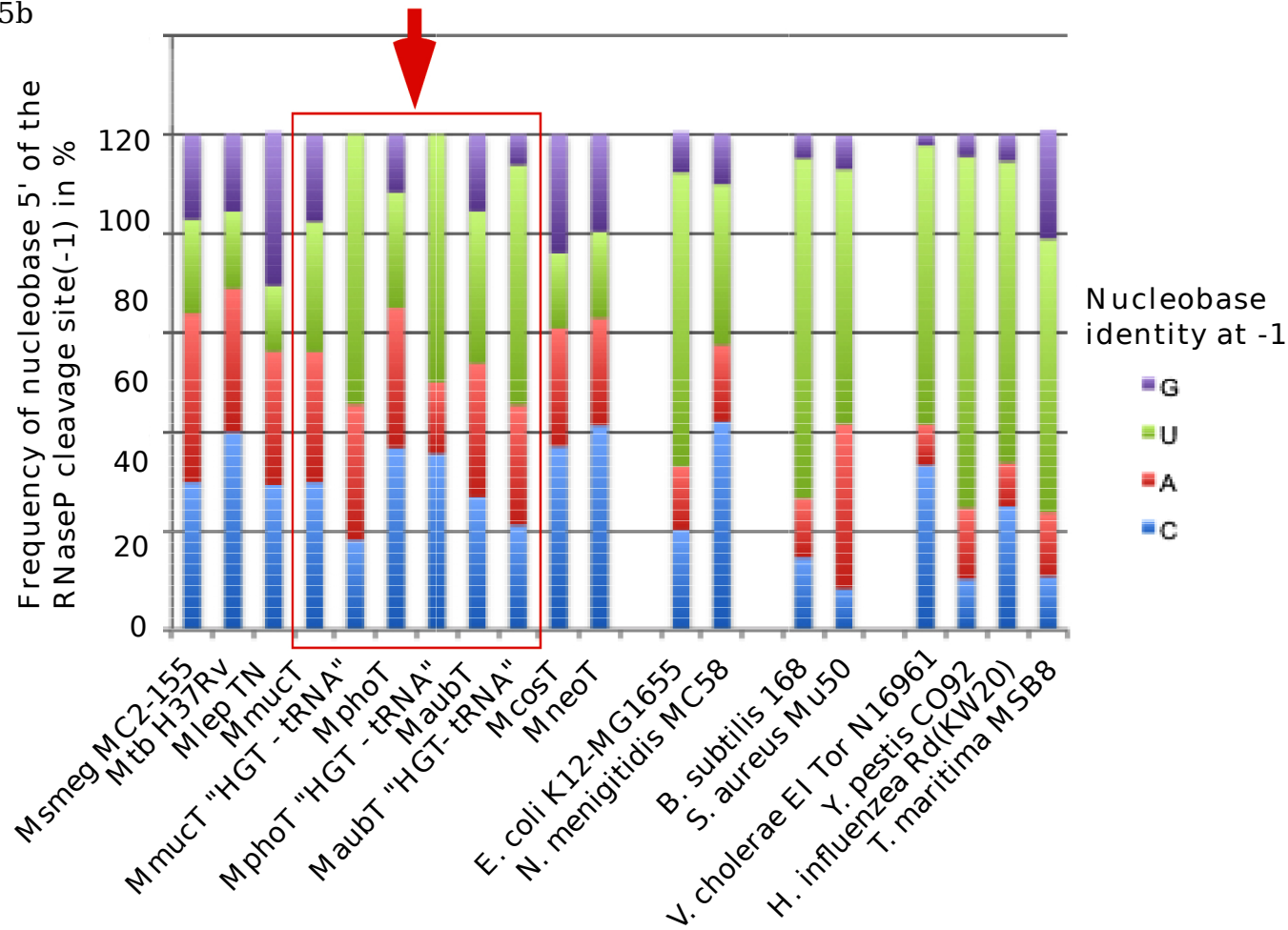

Supplement: Supplementary file 5 — Introduction. Figure legends. Figure S14a, b. Comparing positioning of tRNA genes in MmucT, MtbH37Rv and MsmegMC2-155. Figure S15a, b. Frequency of the identity of the nucleobase at position -1 in tRNA genes [35]. (ZIP 3300 kb) [file 12862_2019_1447_MOESM5_ESM.zip › 12862_2019_1447_MOESM5_ESM/Fig S15ab.pdf]
