# Supplementary material for: Comparative genomics of Mycobacterium mucogenicum and Mycobacterium neoaurum clade members emphasizing tRNA and non-coding RNA
Source: BMC Evol Biol. 2019 Jun 18;19:124. doi: 10.1186/s12862-019-1447-7 (PMC6582537; doi:10.1186/s12862-019-1447-7)
Supplement: Supplementary file 2 — Introduction. Figure legends. Figure S2a, b. Functional classification of genes. Figure S3a, b. Phylogenetic analysis. (ZIP 151 kb) [file 12862_2019_1447_MOESM2_ESM.zip › 12862_2019_1447_MOESM2_ESM/ADDITIONAL FILE 2 INTRODUCTION.pdf]

Additional file 2: Introduction. Table and Figure legends, Additional file 2.

Figure S2. Functional classification of genes.

Functional classification of core genes into subsystems for *Mmuc*^{T}. The x-axis represents the number of core genes in percentage while numbers represents number of genes in the different categories. (a) 291 core genes present in 109 mycobacteria, (b) 2226 core genes present in 17 *Mmuc*- and *Mneo*-clade members.

Figure S3. Phylogenetic analysis.

(a) Phylogenetic tree based on core genes predicted (291) to be present in 109 mycobacteria. Red colored bar mark SGM while blue correspond to RGM. The percentage values in the nodes in both (a) and (b) represent bootstrap values generated by 1000 cycles.

(b) Phylogenetic tree based on the 16S rRNA gene encompassing 112 mycobacteria.
