# Supplementary material for: Comparative genomics of Mycobacterium mucogenicum and Mycobacterium neoaurum clade members emphasizing tRNA and non-coding RNA
Source: BMC Evol Biol. 2019 Jun 18;19:124. doi: 10.1186/s12862-019-1447-7 (PMC6582537; doi:10.1186/s12862-019-1447-7)
Supplement: Supplementary file 3 — Introduction. Table and Figure legends. Table S5. Compilation of predicted tRNA genes in the "32 tRNA gene cluster". Table S6a. Compilation of predicted aminoacyl-tRNA synthetases (AARS) paralogs. Table S6b. Compilation of predicted genes encoding GatCAB enzymes. Table S6c. Compilation of regular and extra gene copy aminoacyl-tRNA synthetase genes. Supplementary text information. Prediction of genes encoding aminoacyl-tRNA synthetase paralogs and cyclodipeptide synthetase genes in Mmuc- and Mneo-clade members. Figure S4a-e. Analysis of tRNA genes [90]. Figure S5. tRNA sequence alignment for all tRNA genes. Figure S6a-f. Analysis of isoleucyl-tRNA synthetase and selected AARS genes. Figure S7a, b. Cyclodipeptide synthase (CDPS) – PF16715 [106, 107]. (ZIP 166 kb) [file 12862_2019_1447_MOESM3_ESM.zip › 12862_2019_1447_MOESM3_ESM/ADDITIONAL FILE 3 INTRODUCTION.pdf]

Additional file 3: Introduction. Table and Figure legends, Additional file 3.

Table S5. Compilation of predicted tRNA genes in the "32 tRNA gene cluster" in *Maub*^{T}, *M. absessus* M24 and *M. conceptionense* MLE. Numbers 1, 2 and 3 refer to 1, 2 and 3 in Figs S4c and S11b.

Table S6a. Compilation of predicted aminoacyl-tRNA synthetases (AARS) paralogs.

Table S6b. Compilation of genes encoding GatCAB enzymes predicted to present in *Mmuc*- and *Mneo*-clade members.

Table S6c. Compilation of regular and extra gene copy, aminoacyl-tRNA synthetase gene pairwise blast results for *Mmuc*- and *Mneo*-clade members.

Supplementary text information. Prediction of genes encoding aminoacyl-tRNA synthetase paralogs and cyclodipeptide synthetase genes in *Mmuc*- and *Mneo*-clade members (see also Table S6a, c and Fig S6b-f).

Figure S4. Analysis of tRNA genes.

(a) Compilation of the number of tRNA genes in *Mmuc*- and *Mneo*-clade members compared to *Mtb*H37Rv and *Msmeg*MC^{2}-155. Green indicates presence and grey absence of the tRNA genes indicated on the right.

(b) Mapping of tRNA genes present in *Mpho*^{T}. Blue boxes and arrows mark tRNA genes that are transcribed from the leading (+) strand, while red marks those that are transcribed from the lagging (-) strand.

(c) Mapping of tRNA genes present in *Maub*^{T} as in (b). Shaded tRNA genes labelled 1 to 3 mark tRNA genes constituting the "32 tRNA gene" cluster. These tRNA genes were predicted to be present on three different scaffolds and after comparing the large tRNA gene clusters present in *M. abscessus* M24 (34 tRNA genes; [90]) and *M. conceptionense* MLE (37 tRNA genes; NCBI bioproject id PRJNA288077) we infer

that the shaded tRNA genes constitute the "32 tRNA gene" cluster (see main text and Additional file 3; Fig S11b).

(d) Mapping of tRNA genes present in *Mcos*^{T} as in (b).

(e) Mapping of tRNA genes present in *Mneo*^{T} as in (b).

Figure S5. tRNA sequence alignment for all tRNA genes.

Sequence alignments for predicted tRNA genes in *Mmuc*- and *Mneo*-clade members and *Mtb*H37Rv and *Msmeg*MC^{2}-155.

Figure S6. Analysis of isoleucyl-tRNA synthetase and selected AARS genes.

(a) Sequence alignment for the isoleucyl-tRNA-synthetase gene, *ileS*, from the five type strains *Mmuc*^{T}, *Mpho*^{T}, *Maub*^{T}, *Mneo*^{T}, *Mcos*^{T} and *Mtb*H37Rv. The "signatures" that classify *ileS* as eukaryotic like as indicated.

(b) Gene synteny for the prolyl-tRNA synthetase gene, *proS*, in *M. spp*. URHB0044 and *Mneo*^{T}. *proS* is highlighted in green.

(c) Gene synteny for the *proS* paralog in *M. spp*. URHB0044 and the five type strains *Mmuc*^{T}, *Mpho*^{T}, *Maub*^{T}, *Mneo*^{T} and *Mcos*^{T}. Green marks the *proS* paralog.

(d) Gene synteny for the arginyl-tRNA synthetase gene, *argS*, and the *argS* paralog in *Mllat*. Green mark genes of interest, while red marks the gene encoding tRNA^{Arg}CCA.

(e) Gene synteny for *argS* in *Mllat* and in the five type strains *Mmuc*^{T}, *Mpho*^{T}, *Maub*^{T}, *Mneo*^{T} and *Mcos*^{T}. Green marks the *argS* and red marks the gene encoding tRNA^{Arg}CCA.

(f) Gene synteny for the glutamyl-tRNA synthetase gene, *gltX*, and the *gltX* paralog (extra) in *Mmuc*^{T}. Green highlight *gltX* and the *gltX* paralog, while red marks the gene encoding tRNA^{Gln}CTG and tRNA^{Glu}CTC.

Figure S7. Cyclodipeptide synthase (CDPS) – PF16715.

(a) Sequence alignment of the cyclodipeptide synthase (CDPS) PF16715 from *Mtb*H37Rv and *Mneo*^{T}. The difference in amino acid sequence suggests that these two CDPS possibly are of different origin.

(b) Presence of CDPS domain structures in *Mtb*H37Rv and *Mneo*^{T} cyclodipeptide synthase based on data extracted from the Pfam and string databases [106,107].
