# Supplementary material for: Comparative genomics of Mycobacterium mucogenicum and Mycobacterium neoaurum clade members emphasizing tRNA and non-coding RNA
Source: BMC Evol Biol. 2019 Jun 18;19:124. doi: 10.1186/s12862-019-1447-7 (PMC6582537; doi:10.1186/s12862-019-1447-7)
Supplement: Supplementary file 3 — Introduction. Table and Figure legends. Table S5. Compilation of predicted tRNA genes in the "32 tRNA gene cluster". Table S6a. Compilation of predicted aminoacyl-tRNA synthetases (AARS) paralogs. Table S6b. Compilation of predicted genes encoding GatCAB enzymes. Table S6c. Compilation of regular and extra gene copy aminoacyl-tRNA synthetase genes. Supplementary text information. Prediction of genes encoding aminoacyl-tRNA synthetase paralogs and cyclodipeptide synthetase genes in Mmuc- and Mneo-clade members. Figure S4a-e. Analysis of tRNA genes [90]. Figure S5. tRNA sequence alignment for all tRNA genes. Figure S6a-f. Analysis of isoleucyl-tRNA synthetase and selected AARS genes. Figure S7a, b. Cyclodipeptide synthase (CDPS) – PF16715 [106, 107]. (ZIP 166 kb) [file 12862_2019_1447_MOESM3_ESM.zip › 12862_2019_1447_MOESM3_ESM/Behra et al SUPP INFO EVOL-BIOL-00281 R1.pdf]

Additional file 3: Supplementary text information

Prediction of additional aminoacyl-tRNA synthetase genes, see also Additional file 3: Fig S6b-f and Table S6a, b.

Cited references below are listed at the end of the text.

Prediction of additional aminoacyl-tRNA synthetase (AARS) genes

In *M. sp*. URHB0044 we predicted two prolyl-tRNA synthetase (ProRS) genes. The "extra" ProRS gene in *M. sp*. URHB0044 is located in a region where five genes, including *sigJ*, seem to be unique for this species (relative to the other 16 species; Additional file 3: Fig S6b, c).

We also predicted the presence of two arginyl-tRNA synthetase (ArgRS) genes in *Mllat*. The extra ArgRS gene appears to be located in a region unique to *Mllat* since we were unable to detect homologs for several of the genes present in this region in the other mycobacteria (Additional file 3: Fig S6d, e; Table S6a) with one exception. On the basis of NCBI blast search we detected the presence of several of these genes, including the "extra" ArgRS gene, in the recently released *Mmuc*^{LZSF01} genome (see main text and discussion). Noteworthy, the "common" ArgRS gene is located next to a tRNA^{Arg}CCA gene in *Mllat* as well as in the type strains *Mmuc*^{T}, *Mpho*^{T}, *Maub*^{T}, *Mneo*^{T} and *Mcos*^{T} (Additional file 3: Fig S6e).

Two glutamyl-tRNA synthetase (GluRS) genes were also predicted in members belonging to the *Mmuc*- and *Mneo*-clades. This is in contrast to *Mtb*H37Rv and other SGM, which harbors only one GluRS gene (Fig 5a; unpublished data). However, the predicted "extra" GluRS gene is shorter than the regular GluRS gene. Also, the regular GluRS gene is positioned next to two tRNA genes encoding tRNA^{Gln}CTG and tRNA^{Glu}CTC (Additional file

3: Fig S6f). In this context, the GluRS paralog glutamyl-Q-tRNA^{Asp} synthetase is involved in modification of tRNA^{Asp} in *E. coli* [1].

AARS orthologous has been reported to be present in other mycobacteria such as *Mtb*H37Rv and *Msmeg*MC^{2}155. Even *M. leprae*, with its small genome size (3.2 Mbp), carries CysRS and LysRS orthologous [2]. The presence of two aminoacyl-tRNA-synthetases for certain amino acids in mycobacteria raises the question whether the second copy is functioning as a synthetase or if it has some other function related to tRNA, and/or amino acylated tRNA (see above, [1]). In this context, the paralog LysRS GenX in *E. coli* is suggested to be involved in modification of a conserved lysine to hypussine on translational elongation factor P [3, 4]. To conclude, understanding the function of AARS paralogous warrants further studies.

References, additional material

1. Katz A, Elgamal S, Rajkovic A, Ibba M. Non-canonical roles of tRNAs and tRNA mimics in bacterial cell biology. Mol Microbiol. 2016;101:545–58.

2. Ravishankar S, Ambady A, Swetha RG, Anbarasu A, Ramaiah S, Sambandamurthy VK. Essentiality assessment of cysteinyl and lysyl-tRNA synthetases of Mycobacterium smegmatis. PLoS One. 2016;11:e0147188.

3. Ambrogelly A, O'Donoghue P, Söll D, Moses S. A bacterial ortholog of class II lysyl-tRNA synthetase activates lysine. FEBS Lett. 2010;584:3055-60.

4. Bailly M, de Crècy-Lagard V. Predicting the pathway involved in post-translational modification of elongation factor P in a subset of bacterial species. Biol Direct. 2010;5:3.
