# Supplementary material for: Comparative genomics of Mycobacterium mucogenicum and Mycobacterium neoaurum clade members emphasizing tRNA and non-coding RNA
Source: BMC Evol Biol. 2019 Jun 18;19:124. doi: 10.1186/s12862-019-1447-7 (PMC6582537; doi:10.1186/s12862-019-1447-7)
Supplement: Supplementary file 3 — Introduction. Table and Figure legends. Table S5. Compilation of predicted tRNA genes in the "32 tRNA gene cluster". Table S6a. Compilation of predicted aminoacyl-tRNA synthetases (AARS) paralogs. Table S6b. Compilation of predicted genes encoding GatCAB enzymes. Table S6c. Compilation of regular and extra gene copy aminoacyl-tRNA synthetase genes. Supplementary text information. Prediction of genes encoding aminoacyl-tRNA synthetase paralogs and cyclodipeptide synthetase genes in Mmuc- and Mneo-clade members. Figure S4a-e. Analysis of tRNA genes [90]. Figure S5. tRNA sequence alignment for all tRNA genes. Figure S6a-f. Analysis of isoleucyl-tRNA synthetase and selected AARS genes. Figure S7a, b. Cyclodipeptide synthase (CDPS) – PF16715 [106, 107]. (ZIP 166 kb) [file 12862_2019_1447_MOESM3_ESM.zip › 12862_2019_1447_MOESM3_ESM/Fig S6a.pdf]

Fig S6a

First half of Rossman fold

Second half of Rossman fold

Eukaryote (Type2), Archaebacteria

```
MCOS_05547/1-1045      1 ---------VASYPKPTGGSPNFPALEADVLEYWAADDTFRASIDRRDGAPEYVFYDGPPFANGLPHYGHLLTGYVKDIVPRYRTMRGFKVERRFGWDTHGLPA 95
MNEO_01128/1-1055      1 ---VTADPKSAAYPKPAAGTPNFPALEAEVLDYWGADDTFRASIARRDGAPEYVFYDGPPFANGLPHYGHLLTGYVKDIVPRYRTMRGYKVDRRFGWDTHGLPA 101
MMUC_02754/1-1056      1 VLTALET-DVSAYPKTASGTPNFPDLETEVLEFWARDDTFRASIARRDGAPEYVFYDGPPFANGLPHYGHLLTGYVKDIVPRYRTMRGYKVERRFGWDTHGLPA 103
MPHO_02527/1-1051      1 ---------VSAYPKTASGTPNFPDLETEVLEFWARDDTFRASIARRDGAPEYVFYDGPPFANGLPHYGHLLTGYVKDIVPRYRTMRGYKVERRFGWDTHGLPA 95
MAUB_02862/1-1061      1 VLTALETPDVSAYPKTASGTPNFPDLETEVLEFWARDDTFRASIARRDGAPEYVFYDGPPFANGLPHYGHLLTGYVKDIVPRYRTMRGFKVERRFGWDTHGLPA 104
MtbH37Rv_1536/1-1041   1 -------MTDNAYPKLAGGAPDLPALELEVLDYWSRDDTFRASIARRDGAPEYVFYDGPPFANGLPHYGHLLTGYVKDIVPRYRTMRGYKVERRFGWDTHGLPA 97

MCOS_05547/1-1045     96 ELEVQRQLGIADKAQIEQMGIEKFNDACRASVLKYTDEWRAYVTRQARWVDFDNDYKTLDLGFMESVIWAFKQLWDKGLAYEGNRVLPYCWNDETPLSNHELRM 199
MNEO_01128/1-1055    102 ELEVQRQLGITDKNQIEELGIEKFNDACRASVMKYAGEWRAYVTRQARWVDFDNDYKTMDLDFMESTIWAFKQLWDKGLAYQGNRVLPYCWNDETPLSNHELRM 205
MMUC_02754/1-1056    104 ELEVQRQLGISDKAQIEAMGIGAFNDACRASVMKYSNEWQSYVTRQARWVDFDNDYKTLDLTFMESVIWAFKQLWDKGLAYEGNRVLPYCWNDETPLSNHELRM 207
MPHO_02527/1-1051     96 ELEVQRQLGISDKAQIEAMGIGAFNDACRASVMKYSNEWQSYVTRQARWVDFDNDYKTLDLTFMESVIWAFKQLWDKGLAYEGNRVLPYCWNDETPLSNHELRM 199
MAUB_02862/1-1061    105 ELEVQRQLGISDKAQIEAMGIGAFNDACRASVMKYSNEWQAYVTRQARWVDFDNDYKTLDLTFMESVIWAFKQLWDKGLAYEGNRVLPYCWNDETPLSNHELRM 208
MtbH37Rv_1536/1-1041  98 ELEVERQLGITDKSQIEAMGIAAFNDACRASVLRYTDEWQAYVTRQARWVDFDNDYKTLDLAYMESVIWAFKQLWDKGLAYEGYRVLPYCWRDETPLSNHELRM 201

MCOS_05547/1-1045    200 DDDVYQSRQDPALTVGFRATDGP----VAGSHLLIWTTTPWTLPSNQAVAVNPEVTYVAVDGPDGNRYVLAEARLAAYARELGEEPVVVGSYPGAALVGTRYTP 299
MNEO_01128/1-1055    206 DDDVYQSRQDPALTVGFKAVDGP----AAGAYLLIWTTTPWTLPSNQAVAVNPGVSYVVVDGPDGHRFVLAEARLGAYARELGEEPVVVATYLGEQLLGTRYLP 305
MMUC_02754/1-1056    208 DDDVYQSRQDPALTVGFVATDGP----VAGARLLVWTTTPWTLPSNQAVAVNPEVTYVQVVGPDEQNYVLAEPRLAAYAREFGEEPVIVGSYLGAELLGTHYLP 307
MPHO_02527/1-1051    200 DDDVYQSRQDPALTVGFVATDGP----VAGARLLVWTTTPWTLPSNQAVAVHPEVTYVQVVGPDEQNYVLAEPRLAAYAREFGEEPVVVGSYLGAELLGTQYLP 299
MAUB_02862/1-1061    209 DDDVYQSRQDPALTVGFLATDGP----VAGARLLVWTTTPWTLPSNQAVAVNPEVTYVQVVGPDEQNYVLAEPRLAAYAREFGEEPVVVGSYLGAELLGTHYLP 308
MtbH37Rv_1536/1-1041 202 DDDVYQSRQDPAVTVGFKVVGGQPDNGLDGAYLLVWTTTPWTLPSNLAVAVSPDITYVQVQAGD-RRFVLAEARLAAYARELGEEPVVLGTYRGAELLGTRYLP 304

MCOS_05547/1-1045    300 PFPYFP-------DAVNSFQVLAADFVSTDDGTGIVHMAPAYGEDDKATADTADIVAVTPVDSKGRFDATVPDYAGQHVFDANPQIIRDLKNGTGSAGANGAVL 396
MNEO_01128/1-1055    306 PFAYFAE----SEQARNSFQVLGAEFVSTEDGTGLVHMAPAYGEDDKATADTADIVAVTPVDSKGRFDSTVPDYAGLQVFEANPQIIKDLKNGTGPAAVNAPVL 405
MMUC_02754/1-1056    308 PFAFFVD----SDKAQNAFQVLQGDFVTTEDGTGIVHMAPAYGEDDKATCDAAGIVAVTPVDSTGRFDSTVPDYQGQQVFEANPQIIKDLKNGTGPAAVNAPVL 407
MPHO_02527/1-1051    300 PFAFFVD----SDKAHNAFQVLQGDFVTTEDGTGIVHMAPAYGEDDKATCDAAGIVAVTPVDSTGRFDSTVPDYQGQQVFEANPQIIKDLKNGTGPAAVNAPVL 399
MAUB_02862/1-1061    309 PFAFFVDRGQGSTESNPAFQVLQGDFVTTEDGTGIVHMAPAYGEDDKAACDAAGIVAVTPVDSTGRFDSTVPDYQGQQVFEANPQIIKDLKNGSGPAAVNAPVL 412
MtbH37Rv_1536/1-1041 305 PFAYFMD-------WPNAFQVLAGDFVTTDDGTGIVHMAPAYGEDDMVVAEAVGIAPVTPVDSKGRFDVTVADYQGQHVFDANAQIVRDLKTQSGPAAVNGPVL 401

MCOS_05547/1-1045    397 LRHETYEHSYPHCWRCRNPLIYRAVSSWFIKVTEFRDRMVELNQQITWYPEHVKDGQFGKWLSNARDWSVSRNRYWGSPIPVWKSDDPAYPRIDVYGSLDELER 500
MNEO_01128/1-1055    406 LRQETYDHSYPHCWRCRNPLIYRAVSSWFVKVTQFRDRMVELNQQITWYPEHVKDGQFGKWLSNARDWSISRNRYWGTPIPVWVSDDPAYPRTDVYGSLDELER 509
MMUC_02754/1-1056    408 LRHETYEHSYPHCWRCRNPLIYRAVSSWFVKVTEFRDRMVELNQQITWYPEHVKDGQFGKWLSNARDWSISRNRYWGSPIPVWKSDDPAYPRIDVYGSLDELER 511
MPHO_02527/1-1051    400 LRHETYEHSYPHCWRCRNPLIYRAVSSWFVKVTEFRDRMVELNQQITWYPEHVKDGQFGKWLSNARDWSISRNRYWGSPIPVWKSDDPAYPRIDVYGSLDELER 503
MAUB_02862/1-1061    413 LRHETYEHSYPHCWRCRNPLIYRAVSSWFVKVTEFRDRMVELNQQITWYPEHVKDGQFGKWLSNARDWSISRNRYWGSPIPVWKSDDPAYPRVDVYGSLDELQR 516
MtbH37Rv_1536/1-1041 402 IRHETYEHPYPHCWRCRNPLIYRSVSSWFVRVTDFRDRMVELNQQITWYPEHVKDGQFGKWLQGARDWSISRNRYWGTPIPVWKSDDPAYPRIDVYGSLDELER 505

MCOS_05547/1-1045    501 DFGVRPDNLHRPYIDELTRPNPDDPTGKSMMRRIEDVFDVWFDSGSMPYAQVHYPFENRDWFDGTP----DVEAHFPGDFIVEYIGQTRGWFYTMHVLATALFD 600
MNEO_01128/1-1055    510 DFGVRPDNLHRPFIDELTRPNPDDPTGKSTMRRIEDVLDVWFDSGSMPYAQVHYPFENQKWFDGVSGPDGTEDAHFPGDFIVEYIGQTRGWFYTMHVLSTALFD 613
MMUC_02754/1-1056    512 DFGVRPDNLHRPYIDELTRPNPDDPTGKSTMRRIEDVFDVWFDSGSMPYAQVHYPFENQDWFDGSA----SEEAHFPGDFIVEYIGQTRGWFYTMHVLATALFD 611
MPHO_02527/1-1051    504 DFGVRPDNLHRPYIDELTRPNPDDPTGKSTMRRIEDVFDVWFDSGSMPYAQVHYPFENADWFDGSA----DESAHFPGDFIVEYIGQTRGWFYTMHVLATALFD 603
MAUB_02862/1-1061    517 DFGVRPDNLHRPYIDELTRPNPDDPTGKSTMRRIEDVFDVWFDSGSMPYAQVHYPFENRDWFDGGS----GEIAHFPGDFIVEYIGQTRGWFYTMHVLATALFD 616
MtbH37Rv_1536/1-1041 506 DFGVRPANLHRPYIDELTRPNPDDPTGRSTMRRIPDVLDVWFDSGSMPYAQVHYPFENLDWFQG----------HYPGDFIVEYIGQTRGWFYTLHVLATALFD 599

MCOS_05547/1-1045    601 KPAFKTCVAHGIVLGNDGQKMSKSLRNYPDVSEVFDRDGSDAMRWFLMASPILRGGNLIVTEQGIREGVRQVQLPFWNAYTFLTLYAPKRGVWRTDSTHVLDRY 704
MNEO_01128/1-1055    614 RPAFKTCVSHGIVLGNDGQKMSKSLRNYPDVSEVFDRDGSDAMRWFLMASPILRGGNLIVTEQGIREGVRQVQLPFWNAYTFLTLYAPAKGTWRTDSTHVLDRY 717
MMUC_02754/1-1056    612 RPAFKTCVAHGIVLGNDGQKMSKSLRNYPDVSEVFDRDGSDAMRWFLMASPILRGGNLIVTEQGIRDGVRQVLLPVWNAYSFLALYAPKKGTWRTDSTNVLDRY 715
MPHO_02527/1-1051    604 RPAFKTCVAHGIVLGNDGQKMSKSLRNYPDVSEVFDRDGSDAMRWFLMASPILRGGNLIVTEQGIRDGVRQVLLPVWNAYSFLALYAPKKGTWRTDSTHVLDRY 707
MAUB_02862/1-1061    617 RPAFKTCVAHGIVLGNDGQKMSKSLRNYPDVSEVFDRDGSDAMRWFLMASPILRGGNLIVTEQGIRDGVRQVLLPVWNAYSFLALYAPKKGTWRTDSPHVLDRY 720
MtbH37Rv_1536/1-1041 600 RPAFKTCVAHGIVLGFDGQKMSKSLRNYPDVTEVFDRDGSDAMRWFLMASPILRGGNLIVTEQGIRDGVRQVLLPLWNTYSFLALYAPKVGTWRVDSVHVLDRY 703

MCOS_05547/1-1045    705 ILAKLAALRDDLTDSLDVCDISGACEQLRQFTEALTNWYVRRSRSRFWEEDAEAIDTLHTVLEVTGRLAAPLLPLVSEVIWRGVTGERSVHLTDWPAADLLPAD 808
MNEO_01128/1-1055    718 ILAKLAALRDELTASLDVCDISGACDQLRQFTEALTNWYVRRSRSRFWEEDADAIDTLHTVLEVTGRLAAPLLPLVSEVIWRGVTGQRSVHLTDWPSADELPAD 821
MMUC_02754/1-1056    716 ILAKLADLRDTLTESLDACDISGACEQLRQFAEALTNWYVRRSRSRFWEEDADAIDTLHTVLEVTCRLAAPLLPLATEKMWQGLTDERSVHLTDWPEAGVVPAD 819
MPHO_02527/1-1051    708 ILAKLADLRDTLTDSLDACDISGACEQLRQFAEALTNWYVRRSRSRFWEEDADAIDTLHTVLEVTCRLAAPLLPLATEKMWQGLTDERSVHLTDWPEVGVVPAD 811
MAUB_02862/1-1061    721 ILAKLADLRDTLTESLDACDISGACEQLRQFAEALTNWYVRRSRSRFWEEDADAIDTLHTVLEVTCRLAAPLLPLATEVIWRGLTDERSVHLTDWPEAGVVPAD 824
MtbH37Rv_1536/1-1041 704 ILAKLAVLRDDLSESMEVYDIPGACEHLRQFTEALTNWYVRRSRSRFWAEDADAIDTLHTVLEVTTRLAAPLLPLITEIIWRGLTRERSVHLTDWPAPDLLPSD 807

MCOS_05547/1-1045    809 PDLVAAMDQVREVCSTGSSLRKAKKLRVRLPLPKLTVAVA---DPAALAPFAELIADELNVKAVEFTDDIDAYGRFELAVNARVAGPRIGKDVQAAIKAVKAGA 909
MNEO_01128/1-1055    822 PDLVAAMDQVREVCSTGSSLRKAKKLRVRLPLPKLTVAVQ---NPDSLQPFADLIADELNVKAVELSDEIDTYGRFELAVNARAAGPRIGKDVQAAIKAVKAGD 922
MMUC_02754/1-1056    820 PELVAAMDRVREVCSAGSSLRKAKKLRVRLPLPKLTVAVD---NPEALRPFTDLIADELNVKAVELTDDIDAYGKFELAVNARVAGPRIGKDVQAAIKAVKAGE 920
MPHO_02527/1-1051    812 PELVAAMDRVREVCSAGSSLRKAKKLRVRLPLPKLTVALEKSVNPAVLAPFEDLIADELNVKAVELSDDIDAYGKFELTVNARVAGPRIGKDVQAAIKAVKAGE 915
MAUB_02862/1-1061    825 PELVAAMDRVREVCSAGSSLRKAKKLRVRLPLPKLTVAVD---NPEALRPFVDLIADELNVKAVELTDDIDAYGKFELTVNARVAGPRIGKDVQAAIKAVKAGE 925
MtbH37Rv_1536/1-1041 808 ADLVAAMDQVRDVCSAASSLRKAKKLRVRLPLPKLIVAVE---NPQLLRPFVDLIGDELNVKQVELTDAIDTYGRFELTVNARVAGPRLGKDVQAAIKAVKAGD 908

MCOS_05547/1-1045    910 GVVNPDGTLTAGPAVLLEGEYTAKLVAAEPEWTAALPDGAGLVVLDGTVTPELEAEGWAKDRIRELQELRKATGLDVSDRISVVMAVPDRLAEWADTHRELIAG 1013
MNEO_01128/1-1055    923 GVLNADGTLTAGPATLLPTEFTSKLVAADAEWTAALPDGAGLVVLDGNVTPELEAEGWAKDRIRELQELRKTTGLDVSDRISVRMSVPAQFTEWAAAHRDLIAG 1026
MMUC_02754/1-1056    921 GVVNPDGTLTAGPAVLLPAEYSSKLVAAEPDFTAALPEGAGLVVLDATVTPELEAEGWAKDRIRELQDLRKSTGLDVSDRITVTFAVPADKRAWATTHADLIAG 1024
MPHO_02527/1-1051    916 GVVNPDGTLTAGPAMLLPAEYSSKLVAAEPEFTAALPGGAGLVVLDATVTPELEAEGWAKDRIRELQDLRKATGLDVSDRITVTFAVPADKQQWATTHADLIAG 1019
MAUB_02862/1-1061    926 GVVNPDGTLTAGPAVLLPTEYSSKLVAAEPEFTAALPEGAGLVVLDATVTPELEAEGWAKDRIRELQDLRKATGLEVSDRITVTFAVPADKQAWATAHADLIAG 1029
MtbH37Rv_1536/1-1041 909 GVINPDGTLLAGPAVLTPDEYNSRLVAADPESTAALPDGAGLVVLDGTVTAELEAEGWAKDRIRELQELRKSTGLDVSDRIRVVMSVPAEREDWARTHRDLIAG 1012

MCOS_05547/1-1045   1014 EVLATSFELVGADGLPDGSEIGDGVRVVLAKA 1045
MNEO_01128/1-1055   1027 EILATDFEFG---DVADGHDVGEGVLVTIAKA 1055
MMUC_02754/1-1056   1025 EILATAFNVVDAGELADGSDVGDGVRAAIAKA 1056
MPHO_02527/1-1051   1020 EILATGFTVVDAGELADGADVGDGVRAAIAKA 1051
MAUB_02862/1-1061   1030 EILATDFTVVVATDLADGADVGDGVRAVIAKA 1061
MtbH37Rv_1536/1-1041 1013 EILATDFEFA---DLADGVAIGDGVRVSIEKT 1041
```
