# Supplementary material for: Comparative genomics of Mycobacterium mucogenicum and Mycobacterium neoaurum clade members emphasizing tRNA and non-coding RNA
Source: BMC Evol Biol. 2019 Jun 18;19:124. doi: 10.1186/s12862-019-1447-7 (PMC6582537; doi:10.1186/s12862-019-1447-7)
Supplement: Supplementary file 4 — Introduction. Table and Figure legends. Table S7. Rfam non-coding RNA annotations. Figure S8a, b. ncRNA genes. Figure S9a-i. RNase P RNA (rnpB), tmRNA and 4.5S RNA. Figure S10. Northern blot analysis of selected tRNAs and ncRNAs with size markers. Figure S11a, b. Analysis of GOLLD RNA. Figure S12a-d. Comparison of Ms1 RNA and 6C RNA genes in mycobacteria. Figure S13a, b. Comparison of a intron Group II gene cluster. (ZIP 7530 kb) [file 12862_2019_1447_MOESM4_ESM.zip › 12862_2019_1447_MOESM4_ESM/ADDITIONAL FILE 4 INTRODUCTION.pdf]

Additional file 4: Introduction. Table and Figure legends, Additional file 4.

Table S7. Rfam non-coding RNA annotations for *Mmuc*- and *Mneo*-clade members.

Figure S8. ncRNA genes.

(a) Genome wide distribution and number (see color code) of the RFAM predicted ncRNA genes in *Mmuc*- and *Mneo*-clade members compared to their presence in *Mtb*H37Rv and *Msmeg*MC^2-155 as indicated. The different RFAM RNA categories are given on the x-axis.

(b) Presentation of predicted length of the RFAM RNAs as indicated. The pie chart represents overall distribution (in %) of the different RFAM RNA types in *Mmuc*- and *Mneo*-clade members.

Figure S9. RNase P RNA (*rnpB*), tmRNA and 4.5S RNA.

(a) *rnpB* (marked in red) gene synteny for the five type strains *Mmuc*^{T}, *Mpho*^{T}, *Maub*^{T}, *Mneo*^{T} and *Mcos*^{T} compared to *Mtb*H37Rv. The *rnpA* gene is marked in green.

(b) Sequence alignment of *rnpB* from *Mmuc*- and *Mneo*-clade members.

(c) tmRNA (marked in red) gene synteny for the five type strains *Mmuc*^{T}, *Mpho*^{T}, *Maub*^{T}, *Mneo*^{T} and *Mcos*^{T} compared to *Mtb*H37Rv. The *smpB* gene is marked in green.

(d) Sequence alignment of the tmRNA gene (*ssrA*) and the sequence for the proteolysis tag (highlighted in yellow) encoded by tmRNA as indicated.

(e) Gene synteny for the 4.5S RNA gene (marked in red) for the five type strains *Mmuc*^{T}, *Mpho*^{T}, *Maub*^{T}, *Mneo*^{T} and *Mcos*^{T} compared to *Mtb*H37Rv. The *ffh* gene is marked in green.

(f) Sequence alignment of the 4.5S RNA gene (*ffs*) as indicated.

(g) Phylogenetic tree based on the RNase P RNA (*rnpB*) gene. Red colored bar mark SGM while blue mark RGM.

(h) Phylogenetic tree based on the tmRNA (*ssrA*) gene. Red colored bar mark SGM while blue mark RGM.

(i) Phylogenetic tree based on the 4.5S RNA (*ffs*) gene. Red colored bar mark SGM while blue mark RGM.

Figure S10. Northern blot analysis of selected tRNAs and ncRNAs with size markers as indicated.

Three replicate filters for *Mmuc*^{T} (three rows in the left column) and three for *Maub*^{T} (three rows in the right column) were generated. Each filter had a pUC19-Msp 1 ^{32}P-5'-end labelled size marker and three biological replicates of RNA extracted from exponentially (Exp) growing cells and stationary (Stat) cells (two replicates for *Maub*^{T} Exp cells). The filters were sequentially probed with the ^{32}P-5'-end labelled probes specific for the indicated RNAs (Additional file 1: Table S1) with stripping of the probe between each new probing according to the manufacturers instruction. Arrows mark remaining signals from 5S rRNA that were not possible to remove. To the left of each filter group shows the size marker in base pairs as indicated. Of note, the filters are not arranged in the order they were probed and that the blots for each filter were manually aligned in the y-axis dimension and may be shifted compared to the blot showing the size marker.

Figure S11. Analysis of GOLLD RNA.

(a) Sequence alignment of the GOLLD RNA gene from *Maub*^{T}, *M. abscessus* bolleti M24, *M. conceptionense* MLE, *M. spp*. H110 and *M. ssp*. HXXIII. The GOLLD RNA

gene is located between the genes encoding tRNA^{Leu}CAG and tRNA^{Asn}GTT, highlighted in purple. The blue color marks the predicted tRNA gene located inside the GOLLD RNA gene, while residues highlighted in pink and in green correspond to SigB promoters (-35 and -10 regions). The A and C residues highlighted in red mark the predicted GOLLD RNA 5' end and 3' end, respectively.

(b) Gene synteny for the GOLLD RNA gene in *Maub*^{T}, *M. abscessus* bolleti M24 and *M. conceptionense* MLE. The GOLLD RNA gene is highlighted in red, while genes marked in green correspond to genes encoding tRNA.

Figure S12. Comparison of Ms1 RNA and 6C RNA genes in mycobacteria.

(a) Gene synteny for the Ms1 RNA (marked in red; 6S RNA, marked in orange, for *Scoe*) and 6C RNA (marked in green) genes in selected members of the *Mmuc*- and *Mneo*-clades including the five type strains *Mmuc*^{T}, *Mpho*^{T}, *Maub*^{T}, *Mneo*^{T} and *Mcos*^{T} and *S. coelicolor* A3(2) (*Scoe*).

(b) Sequence alignment for the Ms1 RNA gene from *Mmuc*- and *Mneo* clade members compared to the 6S RNA gene in *S. coelicolor* A3(2).

(c) Mycobacterial phylogenetic tree based on the Ms1 RNA gene indicate its presence in all selected mycobacteria. Noteworthy, *Mycobacterium nebraskense* carries two Ms1 RNA gene copies.

(d) Sequence alignment for the 6C RNA gene from *Mmuc*- and *Mneo*-clade members compared to the 6C RNA gene in *S. coelicolor* A3(2).

Figure S13. Comparison of a intron Group II gene cluster.

(a) Gene synteny for Group II genes in *Mpho*^{T} and *Mtb*H37Rv. The Group II genes are highlighted in green.

(b) Sequence alignment of Group II gene clusters in *Mpho*^{T} and *Mtb*H37Rv as indicated.
