# Supplementary material for: Comparative genomics of Mycobacterium mucogenicum and Mycobacterium neoaurum clade members emphasizing tRNA and non-coding RNA
Source: BMC Evol Biol. 2019 Jun 18;19:124. doi: 10.1186/s12862-019-1447-7 (PMC6582537; doi:10.1186/s12862-019-1447-7)
Supplement: Supplementary file 5 — Introduction. Figure legends. Figure S14a, b. Comparing positioning of tRNA genes in MmucT, MtbH37Rv and MsmegMC2-155. Figure S15a, b. Frequency of the identity of the nucleobase at position -1 in tRNA genes [35]. (ZIP 3300 kb) [file 12862_2019_1447_MOESM5_ESM.zip › 12862_2019_1447_MOESM5_ESM/ADDITIONAL FILE 5 INTRODUCTION.pdf]

Additional file 5: Introduction. Figure legends, Additional file 5.

Figure S14. Comparing positioning of tRNA genes in *Mmuc*^{T}, *Mtb*H37Rv and *Msmeg*MC^{2}-155.

(a) Comparing tRNA gene localization in *Mmuc*^{T} and *Mtb*H37Rv showing rearrangements of tRNA genes. For tRNA gene identification for *Mmuc*^{T}, see figure legend 3. Red marks rearranged tRNA genes while green represent those genes that have maintained their position.

(b) Comparing tRNA gene localization in *Mmuc*^{T} and *Msmeg*MC^{2}-155 showing genome rearrangements. For tRNA gene identification for *Mmuc*^{T}, see figure legend 3.

Figure S15. Frequency of the identity of the nucleobase at position -1 in tRNA genes.

(a) Number of tRNA genes in different bacteria as indicated.

(b) Frequency of nucleobase identity immediately 5' of the RNase P cleavage site (the -1 position) in pre-tRNA transcripts in indicated bacteria (for details see main text and [35] and Refs therein).
