# Supplementary material for: Comparative genomics of Mycobacterium mucogenicum and Mycobacterium neoaurum clade members emphasizing tRNA and non-coding RNA
Source: BMC Evol Biol. 2019 Jun 18;19:124. doi: 10.1186/s12862-019-1447-7 (PMC6582537; doi:10.1186/s12862-019-1447-7)
Supplement: Supplementary file 2 — Introduction. Figure legends. Figure S2a, b. Functional classification of genes. Figure S3a, b. Phylogenetic analysis. (ZIP 151 kb) [file 12862_2019_1447_MOESM2_ESM.zip › 12862_2019_1447_MOESM2_ESM/Fig S3b.zip!/Fig S3a.pdf]

Fig S3a

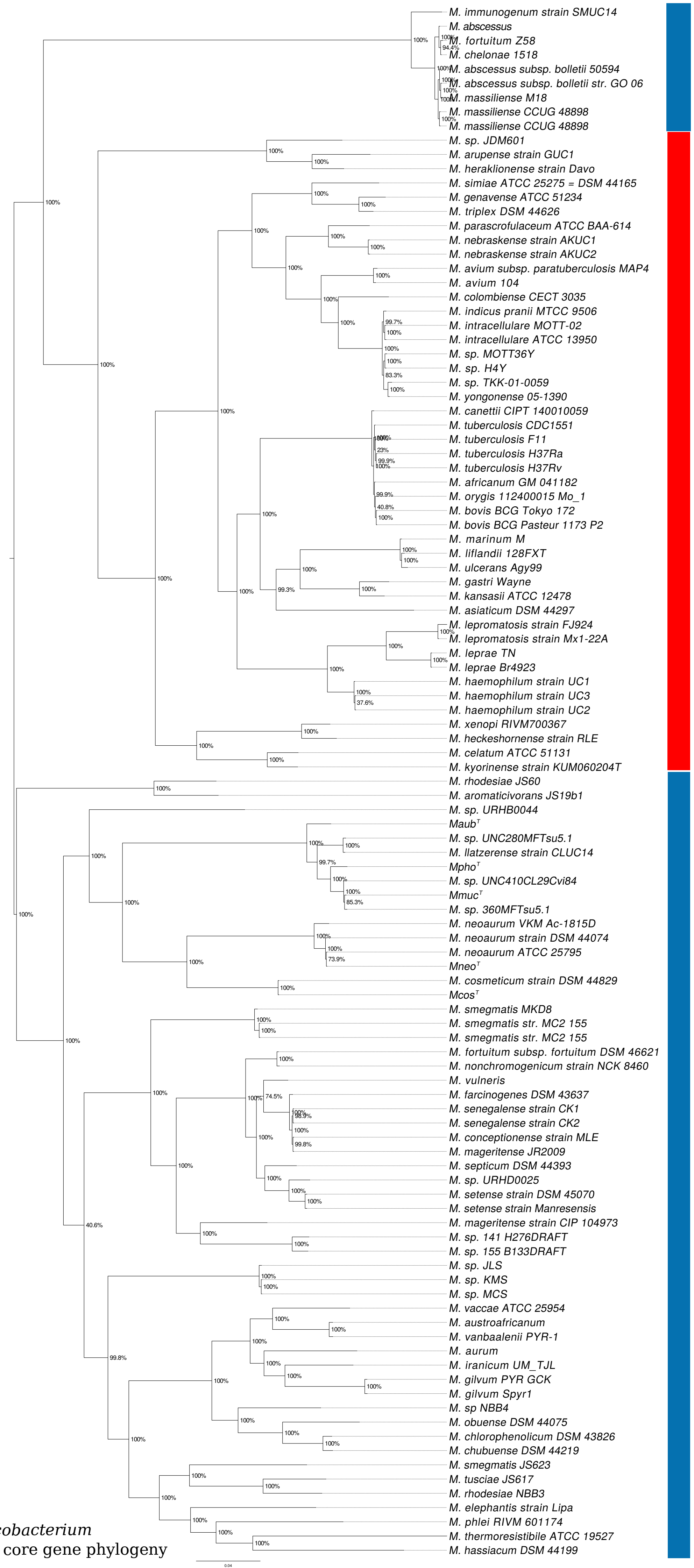

Mycobacterium
291 core gene phylogeny
