# Supplementary material for: Comparative genomics of Mycobacterium mucogenicum and Mycobacterium neoaurum clade members emphasizing tRNA and non-coding RNA
Source: BMC Evol Biol. 2019 Jun 18;19:124. doi: 10.1186/s12862-019-1447-7 (PMC6582537; doi:10.1186/s12862-019-1447-7)
Supplement: Supplementary file 2 — Introduction. Figure legends. Figure S2a, b. Functional classification of genes. Figure S3a, b. Phylogenetic analysis. (ZIP 151 kb) [file 12862_2019_1447_MOESM2_ESM.zip › 12862_2019_1447_MOESM2_ESM/Fig S3b.zip!/Fig S3b.pdf]

Fig S3b

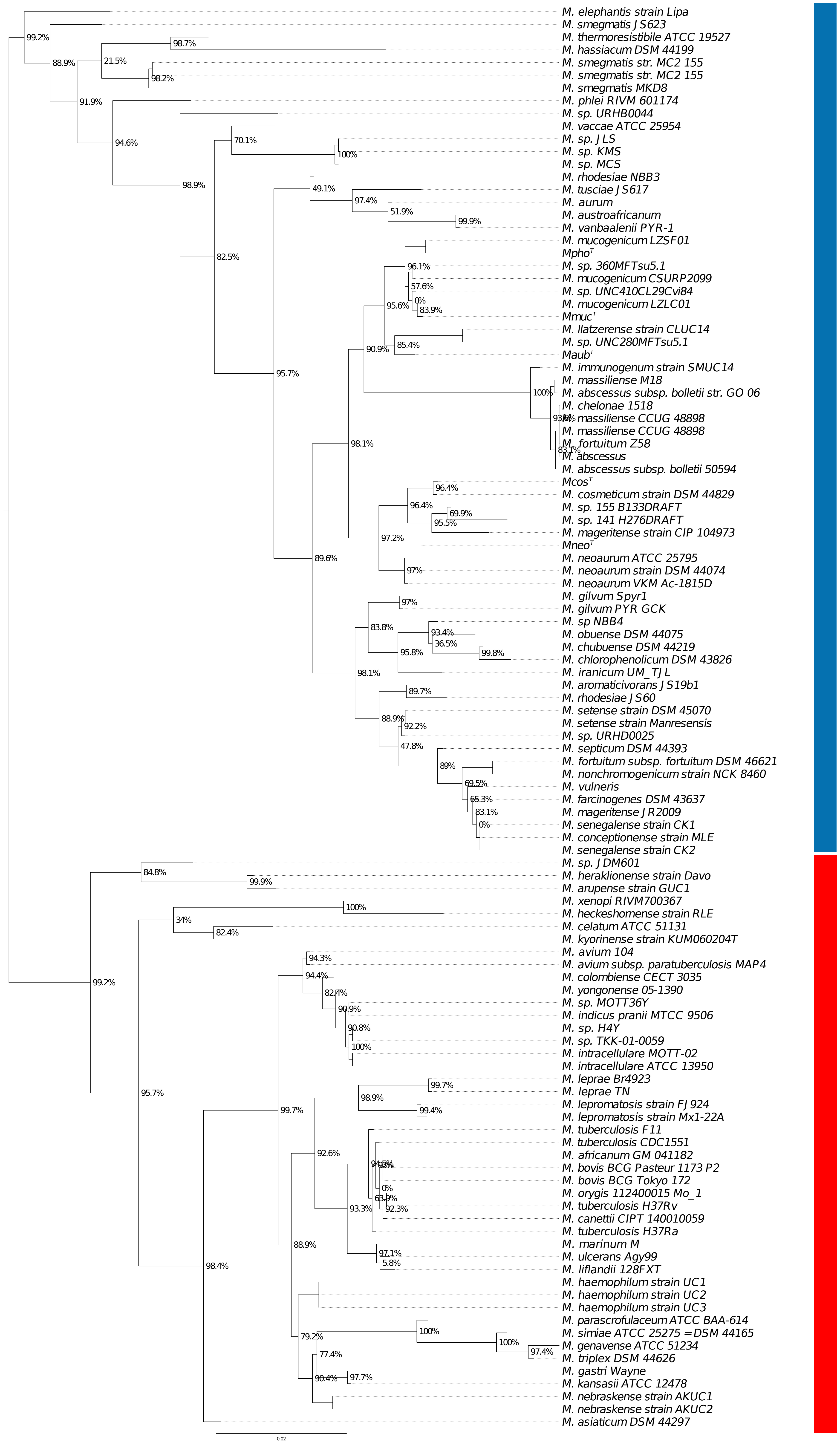
M. elephantis strain Lipa
M. smegmatis JS623
M. thermoresistibile ATCC 19527
M. hassiacum DSM 44199
M. smegmatis str. MC2 155
M. smegmatis str. MC2 155
M. smegmatis MKD8
M. phlei RIVM 601174
M. sp. URHB0044
M. vaccae ATCC 25954
M. sp. JLS
M. sp. KMS
M. sp. MCS
M. rhodesiae NBB3
M. tusciae JS617
M. aurum
M. austroafricanum
M. vanbaalenii PYR-1
M. mucogenicum LZSF01
Mpho^T
M. sp. 360MFTsu5.1
M. mucogenicum CSURP2099
M. sp. UNC410CL29Cvi84
M. mucogenicum LZLC01
Mmuc^T
M. llatzerense strain CLUC14
M. sp. UNC280MFTsu5.1
Maub^T
M. immunogenum strain SMUC14
M. massiliense M18
M. abscessus subsp. bolletii str. GO 06
M. chelonae 1518
M. massiliense CCUG 48898
M. massiliense CCUG 48898
M. fortuitum Z58
M. abscessus
M. abscessus subsp. bolletii 50594
Mcos^T
M. cosmeticum strain DSM 44829
M. sp. 155 B133DRAFT
M. sp. 141 H276DRAFT
M. mageritense strain CIP 104973
Mneo^T
M. neoaurum ATCC 25795
M. neoaurum strain DSM 44074
M. neoaurum VKM Ac-1815D
M. gilvum Spyr1
M. gilvum PYR GCK
M. sp NBB4
M. obuense DSM 44075
M. chubuense DSM 44219
M. chlorophenolicum DSM 43826
M. iranicum UM_TJL
M. aromaticivorans JS19b1
M. rhodesiae JS60
M. setense strain DSM 45070
M. setense strain Manresensis
M. sp. URHD0025
M. septicum DSM 44393
M. fortuitum subsp. fortuitum DSM 46621
M. nonchromogenicum strain NCK 8460
M. vulneris
M. farcinogenes DSM 43637
M. mageritense JR2009
M. senegalense strain CK1
M. conceptionense strain MLE
M. senegalense strain CK2
M. sp. JDM601
M. heraklionense strain Davo
M. arupense strain GUC1
M. xenopi RIVM700367
M. heckeshornense strain RLE
M. celatum ATCC 51131
M. kyorinense strain KUM060204T
M. avium 104
M. avium subsp. paratuberculosis MAP4
M. colombiense CECT 3035
M. yongonense 05-1390
M. sp. MOTT36Y
M. indicus pranii MTCC 9506
M. sp. H4Y
M. sp. TKK-01-0059
M. intracellulare MOTT-02
M. intracellulare ATCC 13950
M. leprae Br4923
M. leprae TN
M. lepromatosis strain FJ924
M. lepromatosis strain Mx1-22A
M. tuberculosis F11
M. tuberculosis CDC1551
M. africanum GM 041182
M. bovis BCG Pasteur 1173 P2
M. bovis BCG Tokyo 172
M. orygis 112400015 Mo_1
M. tuberculosis H37Rv
M. canettii CIPT 140010059
M. tuberculosis H37Ra
M. marinum M
M. ulcerans Agy99
M. liflandii 128FXT
M. haemophilum strain UC1
M. haemophilum strain UC2
M. haemophilum strain UC3
M. parascrofulaceum ATCC BAA-614
M. simiae ATCC 25275 =DSM 44165
M. genavense ATCC 51234
M. triplex DSM 44626
M. gastri Wayne
M. kansasii ATCC 12478
M. nebraskense strain AKUC1
M. nebraskense strain AKUC2
M. asiaticum DSM 44297
0.02
