# Supplementary material for: Comparative genomics of Mycobacterium mucogenicum and Mycobacterium neoaurum clade members emphasizing tRNA and non-coding RNA
Source: BMC Evol Biol. 2019 Jun 18;19:124. doi: 10.1186/s12862-019-1447-7 (PMC6582537; doi:10.1186/s12862-019-1447-7)
Supplement: Supplementary file 4 — Introduction. Table and Figure legends. Table S7. Rfam non-coding RNA annotations. Figure S8a, b. ncRNA genes. Figure S9a-i. RNase P RNA (rnpB), tmRNA and 4.5S RNA. Figure S10. Northern blot analysis of selected tRNAs and ncRNAs with size markers. Figure S11a, b. Analysis of GOLLD RNA. Figure S12a-d. Comparison of Ms1 RNA and 6C RNA genes in mycobacteria. Figure S13a, b. Comparison of a intron Group II gene cluster. (ZIP 7530 kb) [file 12862_2019_1447_MOESM4_ESM.zip › 12862_2019_1447_MOESM4_ESM/Fig S12d.zip!/Fig S12b.pdf]

Fig S12b

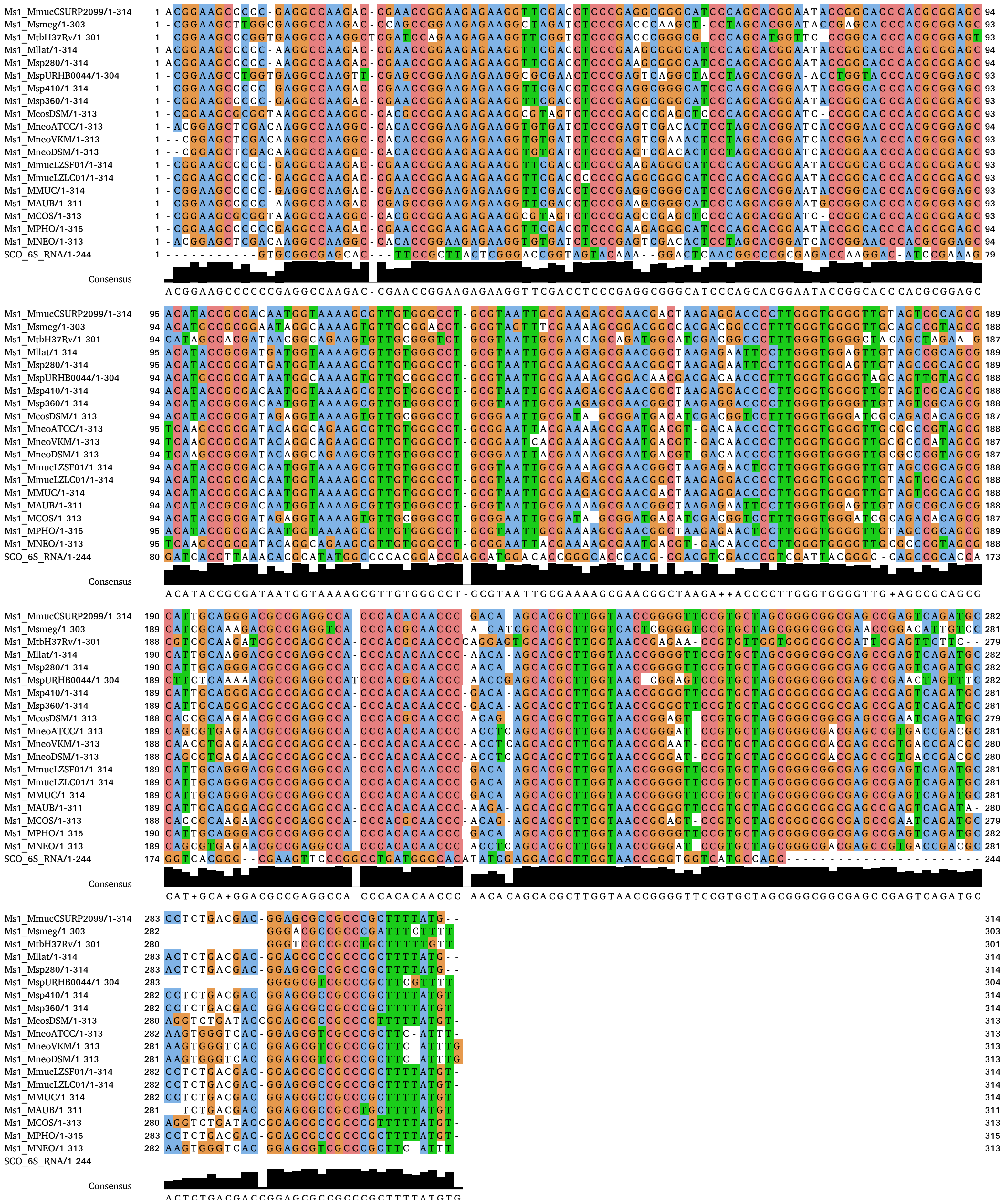
Ms1_MmucCSURP2099/1-314
Ms1_Msmeg/1-303
Ms1_MtbH37Rv/1-301
Ms1_Mllat/1-314
Ms1_Msp280/1-314
Ms1_MspURHB0044/1-304
Ms1_Msp410/1-314
Ms1_Msp360/1-314
Ms1_McosDSM/1-313
Ms1_MneoATCC/1-313
Ms1_MneoVKM/1-313
Ms1_MneoDSM/1-313
Ms1_MmucLZSF01/1-314
Ms1_MmucLZLC01/1-314
Ms1_MMUC/1-314
Ms1_MAUB/1-311
Ms1_MCOS/1-313
Ms1_MPHO/1-315
Ms1_MNEO/1-313
SCO_6S_RNA/1-244
Consensus
