# Supplementary material for: Comparative genomics of Mycobacterium mucogenicum and Mycobacterium neoaurum clade members emphasizing tRNA and non-coding RNA
Source: BMC Evol Biol. 2019 Jun 18;19:124. doi: 10.1186/s12862-019-1447-7 (PMC6582537; doi:10.1186/s12862-019-1447-7)
Supplement: Supplementary file 4 — Introduction. Table and Figure legends. Table S7. Rfam non-coding RNA annotations. Figure S8a, b. ncRNA genes. Figure S9a-i. RNase P RNA (rnpB), tmRNA and 4.5S RNA. Figure S10. Northern blot analysis of selected tRNAs and ncRNAs with size markers. Figure S11a, b. Analysis of GOLLD RNA. Figure S12a-d. Comparison of Ms1 RNA and 6C RNA genes in mycobacteria. Figure S13a, b. Comparison of a intron Group II gene cluster. (ZIP 7530 kb) [file 12862_2019_1447_MOESM4_ESM.zip › 12862_2019_1447_MOESM4_ESM/Fig S12d.zip!/Fig S12c.pdf]

Fig S12c

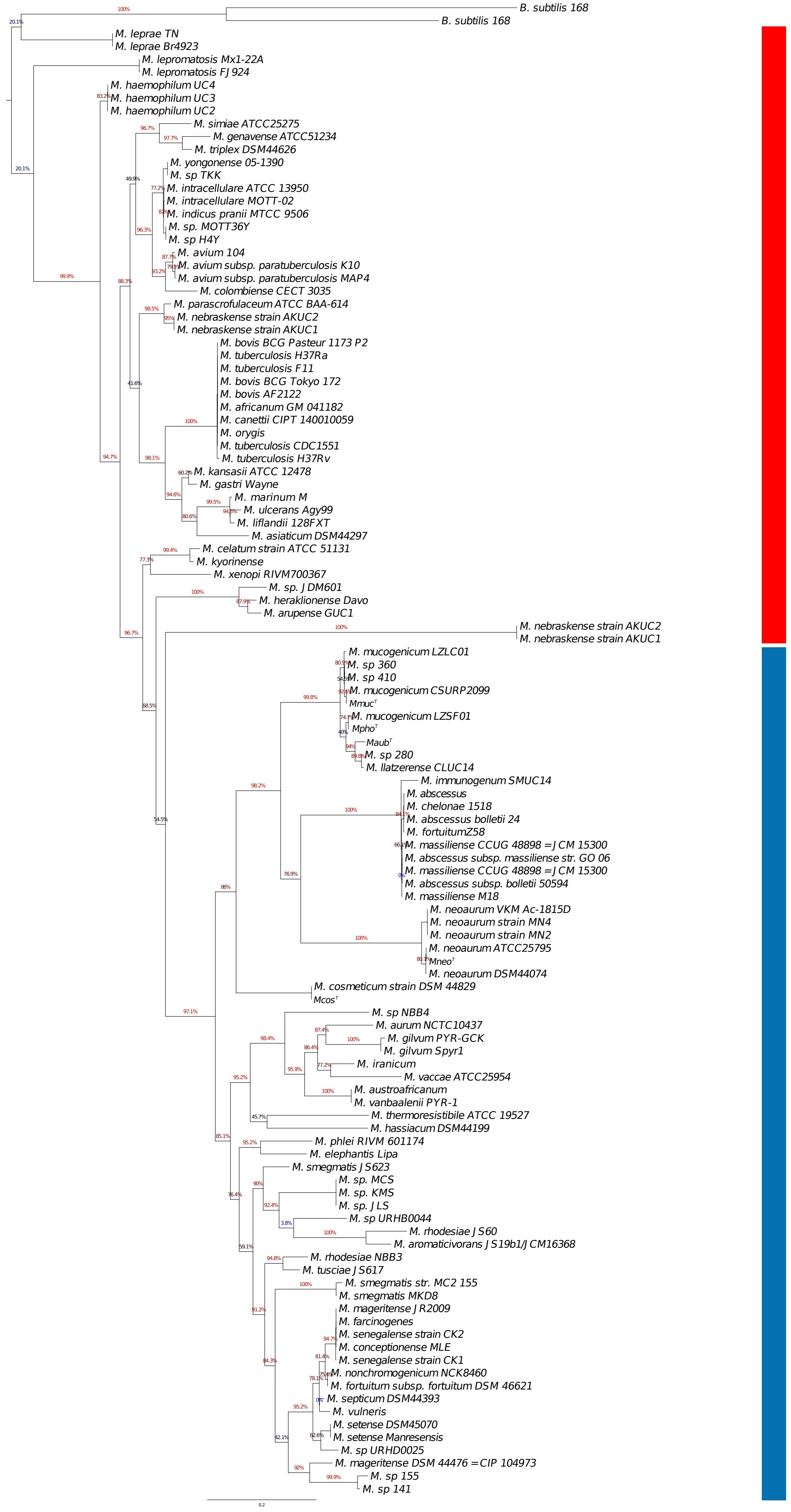

B. subtilis 168
B. subtilis 168
M. leprae TN
M. leprae Br4923
M. lepromatosis Mx1-22A
M. lepromatosis FJ924
M. haemophilum UC4
M. haemophilum UC3
M. haemophilum UC2
M. simiae ATCC25275
M. genavense ATCC51234
M. triplex DSM44626
M. yongonense 05-1390
M. sp TKK
M. intracellulare ATCC 13950
M. intracellulare MOTT-02
M. indicus pranii MTCC 9506
M. sp. MOTT36Y
M. sp H4Y
M. avium 104
M. avium subsp. paratuberculosis K10
M. avium subsp. paratuberculosis MAP4
M. colombiense CECT 3035
M. parascrofulaceum ATCC BAA-614
M. nebraskense strain AKUC2
M. nebraskense strain AKUC1
M. bovis BCG Pasteur 1173 P2
M. tuberculosis H37Ra
M. tuberculosis F11
M. bovis BCG Tokyo 172
M. bovis AF2122
M. africanum GM 041182
M. canettii CIPT 140010059
M. orygis
M. tuberculosis CDC1551
M. tuberculosis H37Rv
M. kansasii ATCC 12478
M. gastri Wayne
M. marinum M
M. ulcerans Agy99
M. liflandii 128FXT
M. asiaticum DSM44297
M. celatum strain ATCC 51131
M. kyorinense
M. xenopi RIVM700367
M. sp. JDM601
M. heraklionense Davo
M. arupense GUC1
M. nebraskense strain AKUC2
M. nebraskense strain AKUC1
M. mucogenicum LZLC01
M. sp 360
M. sp 410
M. mucogenicum CSURP2099
Mmuc
M. mucogenicum LZSF01
Mpho
Maub
M. sp 280
M. llatzerense CLUC14
M. immunogenum SMUC14
M. abscessus
M. chelonae 1518
M. abscessus bolletii 24
M. fortuitumZ58
M. massiliense CCUG 48898 = JCM 15300
M. abscessus subsp. massiliense str. GO 06
M. massiliense CCUG 48898 = JCM 15300
M. abscessus subsp. bolletii 50594
M. massiliense M18
M. neoaurum VKM Ac-1815D
M. neoaurum strain MN4
M. neoaurum strain MN2
M. neoaurum ATCC25795
Mneo
M. neoaurum DSM44074
M. cosmeticum strain DSM 44829
Mcos
M. sp NBB4
M. aurum NCTC10437
M. gilvum PYR-GCK
M. gilvum Spyr1
M. iranicum
M. vaccae ATCC25954
M. austroafricanum
M. vanbaalenii PYR-1
M. thermoresistibile ATCC 19527
M. hassiacum DSM44199
M. phlei RIVM 601174
M. elephantis Lipa
M. smegmatis JS623
M. sp. MCS
M. sp. KMS
M. sp. JLS
M. sp URHB0044
M. rhodesiae JS60
M. aromaticivorans JS19b1/JCM16368
M. rhodesiae NBB3
M. tusciae JS617
M. smegmatis str. MC2 155
M. smegmatis MKD8
M. mageritense JR2009
M. farcinogenes
M. senegalense strain CK2
M. conceptionense MLE
M. senegalense strain CK1
M. nonchromogenicum NCK8460
M. fortuitum subsp. fortuitum DSM 46621
M. septicum DSM44393
M. vulneris
M. setense DSM45070
M. setense Manresensis
M. sp URHD0025
M. mageritense DSM 44476 = CIP 104973
M. sp 155
M. sp 141
0.2
