# Supplementary material for: Comparative genomics of Mycobacterium mucogenicum and Mycobacterium neoaurum clade members emphasizing tRNA and non-coding RNA
Source: BMC Evol Biol. 2019 Jun 18;19:124. doi: 10.1186/s12862-019-1447-7 (PMC6582537; doi:10.1186/s12862-019-1447-7)
Supplement: Supplementary file 4 — Introduction. Table and Figure legends. Table S7. Rfam non-coding RNA annotations. Figure S8a, b. ncRNA genes. Figure S9a-i. RNase P RNA (rnpB), tmRNA and 4.5S RNA. Figure S10. Northern blot analysis of selected tRNAs and ncRNAs with size markers. Figure S11a, b. Analysis of GOLLD RNA. Figure S12a-d. Comparison of Ms1 RNA and 6C RNA genes in mycobacteria. Figure S13a, b. Comparison of a intron Group II gene cluster. (ZIP 7530 kb) [file 12862_2019_1447_MOESM4_ESM.zip › 12862_2019_1447_MOESM4_ESM/Fig S12d.zip!/Fig S12d.pdf]

Fig S12d

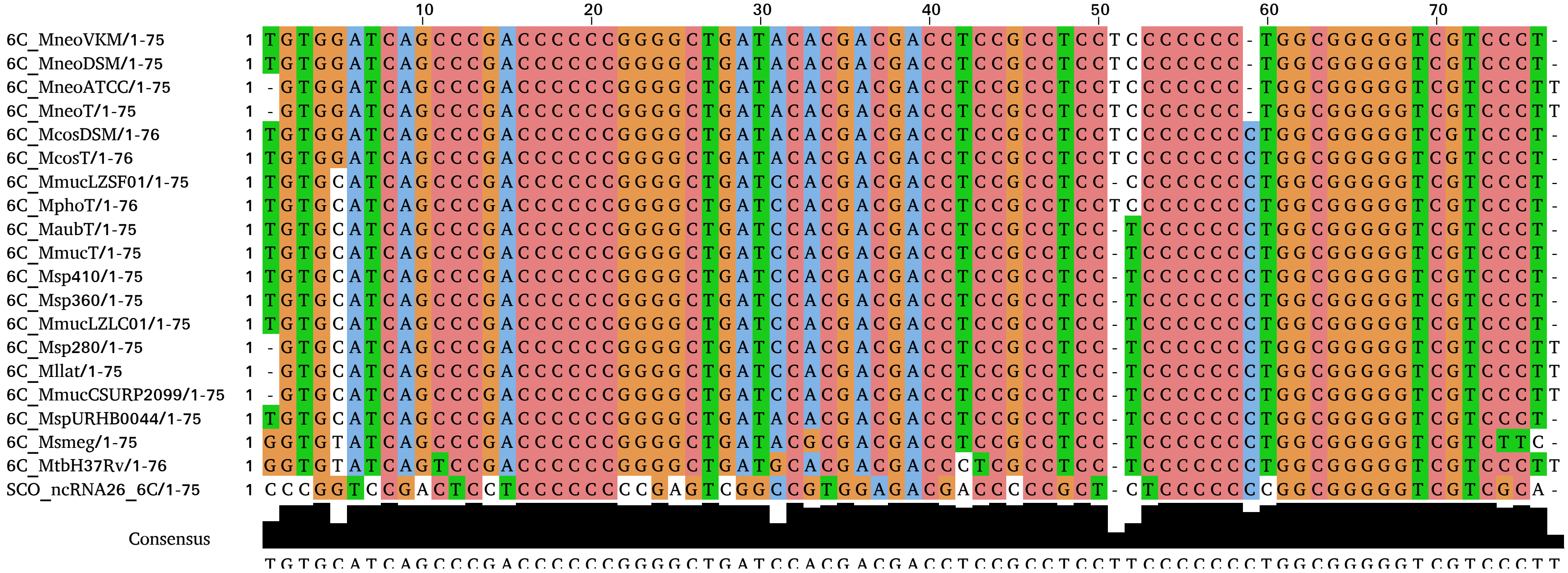
10 20 30 40 50 60 70
6C_MneoVKM/1-75 1 TGTGGATCAGCCCGACCCCCCGGGGCTGATACACGACGACCTCCGCCTCCTCCCCCCC-TGGCGGGGGTCGTCCCT-
6C_MneoDSM/1-75 1 TGTGGATCAGCCCGACCCCCCGGGGCTGATACACGACGACCTCCGCCTCCTCCCCCCC-TGGCGGGGGTCGTCCCT-
6C_MneoATCC/1-75 1 -GTGGATCAGCCCGACCCCCCGGGGCTGATACACGACGACCTCCGCCTCCTCCCCCCC-TGGCGGGGGTCGTCCCTT
6C_MneoT/1-75 1 -GTGGATCAGCCCGACCCCCCGGGGCTGATACACGACGACCTCCGCCTCCTCCCCCCC-TGGCGGGGGTCGTCCCTT
6C_McosDSM/1-76 1 TGTGGATCAGCCCGACCCCCCGGGGCTGATACACGACGACCTCCGCCTCCTCCCCCCCCTGGCGGGGGTCGTCCCT-
6C_McosT/1-76 1 TGTGGATCAGCCCGACCCCCCGGGGCTGATACACGACGACCTCCGCCTCCTCCCCCCCCTGGCGGGGGTCGTCCCT-
6C_MmucLZSF01/1-75 1 TGTGCATCAGCCCGACCCCCCGGGGCTGATCCACGACGACCTCCGCCTCC-CCCCCCCCTGGCGGGGGTCGTCCCT-
6C_MphoT/1-76 1 TGTGCATCAGCCCGACCCCCCGGGGCTGATCCACGACGACCTCCGCCTCCTCCCCCCCCTGGCGGGGGTCGTCCCT-
6C_MaubT/1-75 1 TGTGCATCAGCCCGACCCCCCGGGGCTGATCCACGACGACCTCCGCCTCC-TCCCCCCCTGGCGGGGGTCGTCCCT-
6C_MmucT/1-75 1 TGTGCATCAGCCCGACCCCCCGGGGCTGATCCACGACGACCTCCGCCTCC-TCCCCCCCTGGCGGGGGTCGTCCCT-
6C_Msp410/1-75 1 TGTGCATCAGCCCGACCCCCCGGGGCTGATCCACGACGACCTCCGCCTCC-TCCCCCCCTGGCGGGGGTCGTCCCT-
6C_Msp360/1-75 1 TGTGCATCAGCCCGACCCCCCGGGGCTGATCCACGACGACCTCCGCCTCC-TCCCCCCCTGGCGGGGGTCGTCCCT-
6C_MmucLZLC01/1-75 1 TGTGCATCAGCCCGACCCCCCGGGGCTGATCCACGACGACCTCCGCCTCC-TCCCCCCCTGGCGGGGGTCGTCCCT-
6C_Msp280/1-75 1 -GTGCATCAGCCCGACCCCCCGGGGCTGATCCACGACGACCTCCGCCTCC-TCCCCCCCTGGCGGGGGTCGTCCCTT
6C_Mllat/1-75 1 -GTGCATCAGCCCGACCCCCCGGGGCTGATCCACGACGACCTCCGCCTCC-TCCCCCCCTGGCGGGGGTCGTCCCTT
6C_MmucCSURP2099/1-75 1 -GTGCATCAGCCCGACCCCCCGGGGCTGATCCACGACGACCTCCGCCTCC-TCCCCCCCTGGCGGGGGTCGTCCCTT
6C_MspURHB0044/1-75 1 TGTGCATCAGCCCGACCCCCCGGGGCTGATACACGACGACCTCCGCCTCC-TCCCCCCCTGGCGGGGGTCGTCCCT-
6C_Msmeg/1-75 1 GGTGTATCAGCCCGACCCCCCGGGGCTGATACGCGACGACCTCCGCCTCC-TCCCCCCCTGGCGGGGGTCGTCTTC-
6C_MtbH37Rv/1-76 1 GGTGTATCAGTCCGACCCCCCGGGGCTGATGCACGACGACCCTCGCCTCC-TCCCCCCCTGGCGGGGGTCGTCCCTT
SCO_ncRNA26_6C/1-75 1 CCCGGTCCGACTCCTCCCCCCCCGAGTCGGCCGTGGAGACGACCCCGCT-CTCCCCCCCGGCGGGGGTCGTCGCA-
Consensus
TGTGCATCAGCCCGACCCCCCGGGGCTGATCCACGACGACCTCCGCCTCCTTCCCCCCCTGGCGGGGGTCGTCCCTT
