# Supplementary material for: Comparative genomics of Mycobacterium mucogenicum and Mycobacterium neoaurum clade members emphasizing tRNA and non-coding RNA
Source: BMC Evol Biol. 2019 Jun 18;19:124. doi: 10.1186/s12862-019-1447-7 (PMC6582537; doi:10.1186/s12862-019-1447-7)
Supplement: Supplementary file 4 — Introduction. Table and Figure legends. Table S7. Rfam non-coding RNA annotations. Figure S8a, b. ncRNA genes. Figure S9a-i. RNase P RNA (rnpB), tmRNA and 4.5S RNA. Figure S10. Northern blot analysis of selected tRNAs and ncRNAs with size markers. Figure S11a, b. Analysis of GOLLD RNA. Figure S12a-d. Comparison of Ms1 RNA and 6C RNA genes in mycobacteria. Figure S13a, b. Comparison of a intron Group II gene cluster. (ZIP 7530 kb) [file 12862_2019_1447_MOESM4_ESM.zip › 12862_2019_1447_MOESM4_ESM/Fig S9i.zip!/Fig S9b.pdf]

Fig S9b

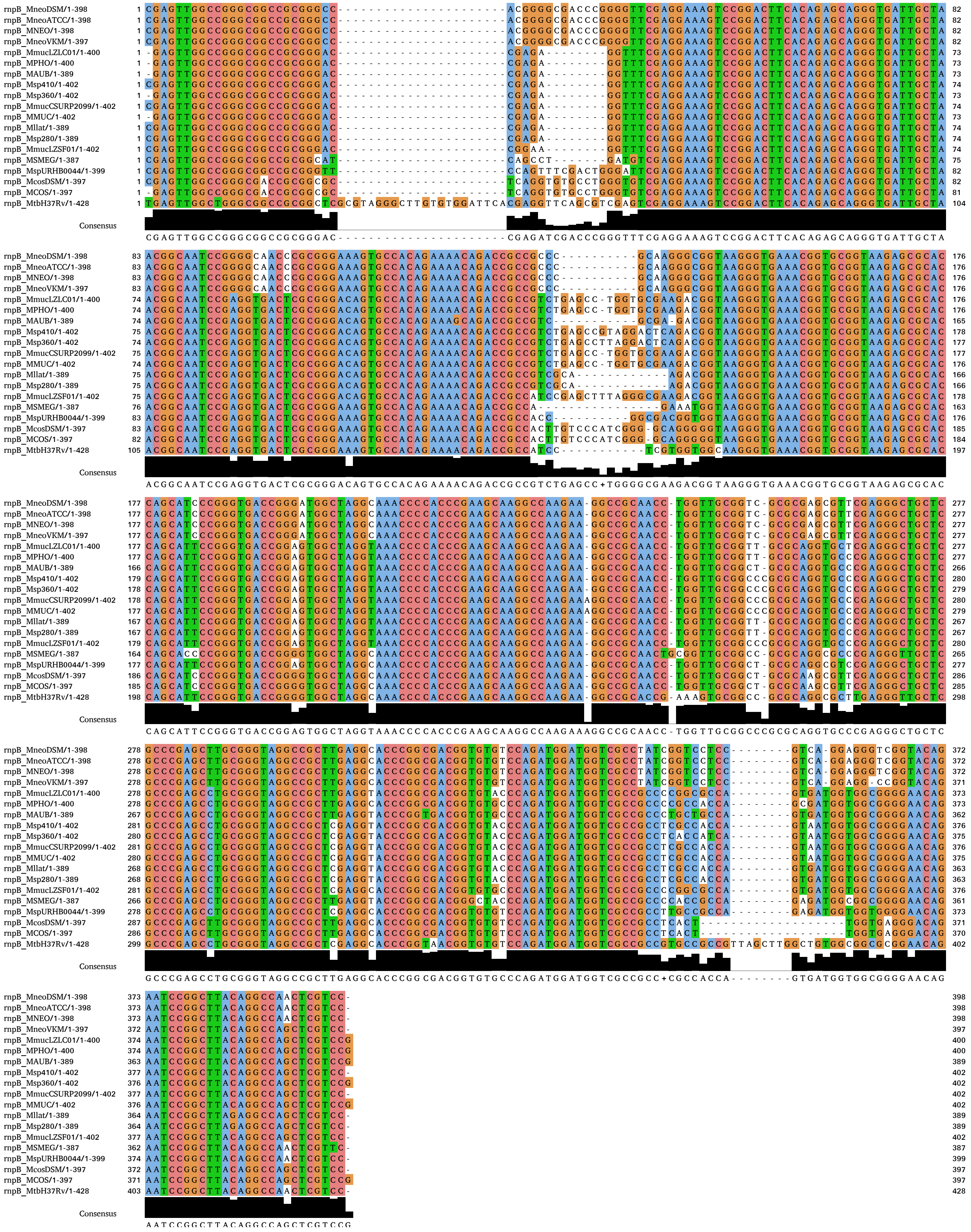
rnpB_MneoDSM/1-398
rnpB_MneoATCC/1-398
rnpB_MNEO/1-398
rnpB_MneoVKM/1-397
rnpB_MmucLZLC01/1-400
rnpB_MPHO/1-400
rnpB_MAUB/1-389
rnpB_Msp410/1-402
rnpB_Msp360/1-402
rnpB_MmucCSURP2099/1-402
rnpB_MMUC/1-402
rnpB_Mllat/1-389
rnpB_Msp280/1-389
rnpB_MmucLZSF01/1-402
rnpB_MSMEG/1-387
rnpB_MspURHB0044/1-399
rnpB_McosDSM/1-397
rnpB_MCOS/1-397
rnpB_MtbH37Rv/1-428
Consensus
