# Supplementary material for: Comparative genomics of Mycobacterium mucogenicum and Mycobacterium neoaurum clade members emphasizing tRNA and non-coding RNA
Source: BMC Evol Biol. 2019 Jun 18;19:124. doi: 10.1186/s12862-019-1447-7 (PMC6582537; doi:10.1186/s12862-019-1447-7)

Fig S9d

tmRNA alignment

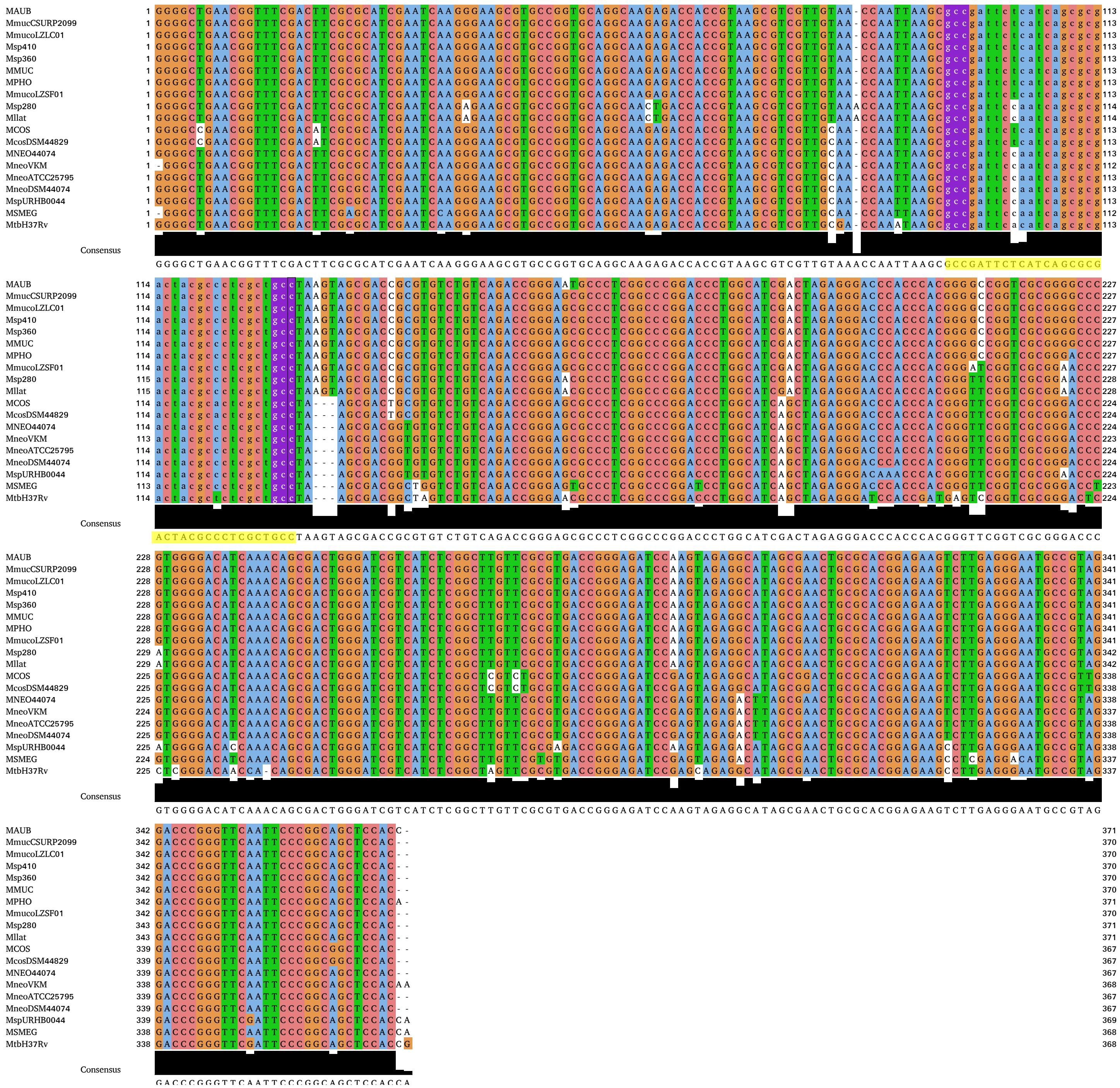

Supplement: Supplementary file 4 — Introduction. Table and Figure legends. Table S7. Rfam non-coding RNA annotations. Figure S8a, b. ncRNA genes. Figure S9a-i. RNase P RNA (rnpB), tmRNA and 4.5S RNA. Figure S10. Northern blot analysis of selected tRNAs and ncRNAs with size markers. Figure S11a, b. Analysis of GOLLD RNA. Figure S12a-d. Comparison of Ms1 RNA and 6C RNA genes in mycobacteria. Figure S13a, b. Comparison of a intron Group II gene cluster. (ZIP 7530 kb) [file 12862_2019_1447_MOESM4_ESM.zip › 12862_2019_1447_MOESM4_ESM/Fig S9i.zip!/Fig S9d.pdf]
