# Supplementary material for: Comparative genomics of Mycobacterium mucogenicum and Mycobacterium neoaurum clade members emphasizing tRNA and non-coding RNA
Source: BMC Evol Biol. 2019 Jun 18;19:124. doi: 10.1186/s12862-019-1447-7 (PMC6582537; doi:10.1186/s12862-019-1447-7)
Supplement: Supplementary file 4 — Introduction. Table and Figure legends. Table S7. Rfam non-coding RNA annotations. Figure S8a, b. ncRNA genes. Figure S9a-i. RNase P RNA (rnpB), tmRNA and 4.5S RNA. Figure S10. Northern blot analysis of selected tRNAs and ncRNAs with size markers. Figure S11a, b. Analysis of GOLLD RNA. Figure S12a-d. Comparison of Ms1 RNA and 6C RNA genes in mycobacteria. Figure S13a, b. Comparison of a intron Group II gene cluster. (ZIP 7530 kb) [file 12862_2019_1447_MOESM4_ESM.zip › 12862_2019_1447_MOESM4_ESM/Fig S9i.zip!/Fig S9e.pdf]

Fig S9e

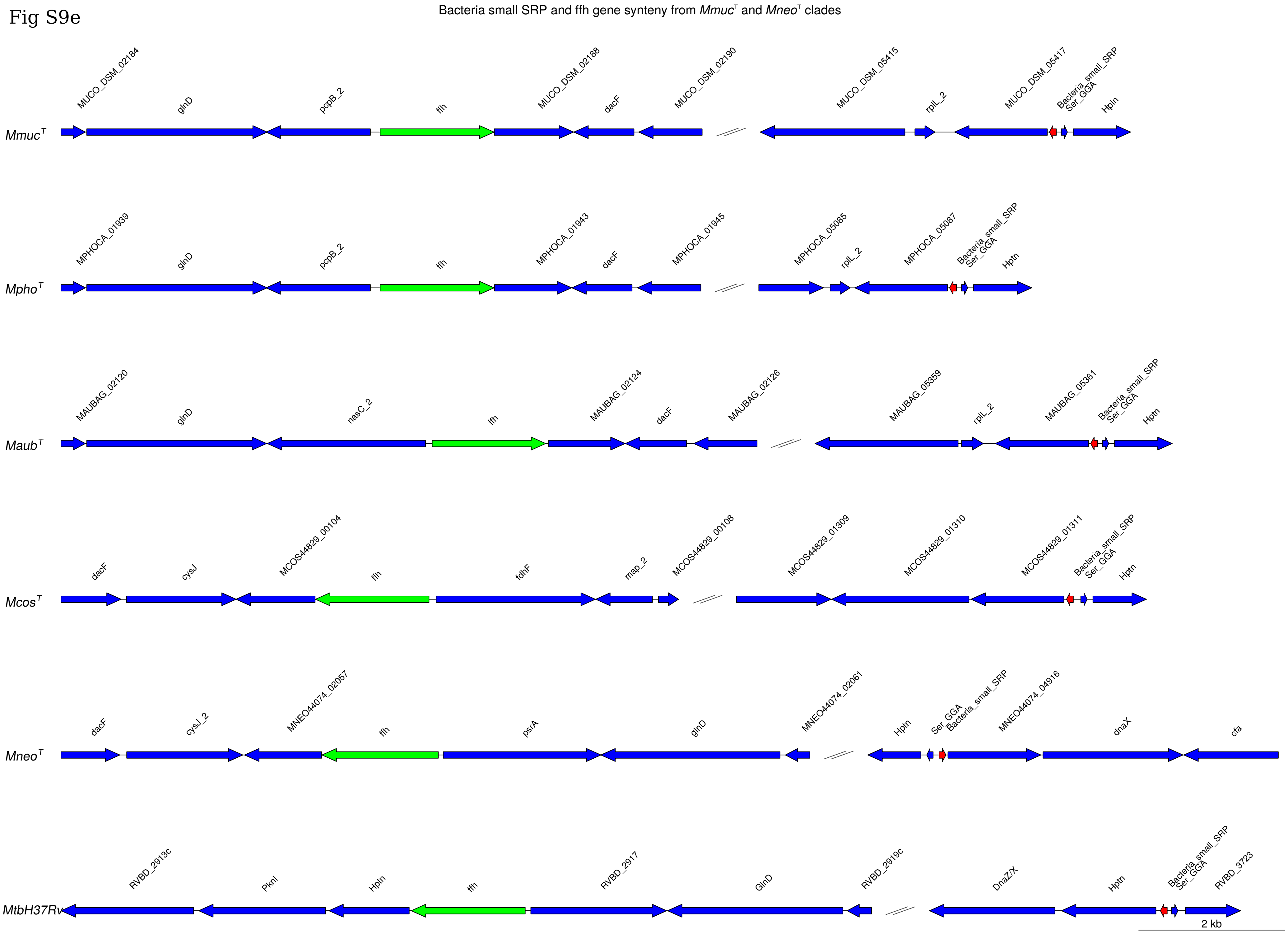
Bacteria small SRP and ffh gene synteny from Mmuc^T and Mneo^T clades
Mmuc^T
MUCO_DSM_02184
glnD
pcpB_2
ffh
MUCO_DSM_02188
dacF
MUCO_DSM_02190
MUCO_DSM_05415
rplL_2
MUCO_DSM_05417
Bacteria_small_SRP
Ser_GGA
Hptn
Mpho^T
MPHOCA_01939
glnD
pcpB_2
ffh
MPHOCA_01943
dacF
MPHOCA_01945
MPHOCA_05085
rplL_2
MPHOCA_05087
Bacteria_small_SRP
Ser_GGA
Hptn
Maub^T
MAUBAG_02120
glnD
nasC_2
ffh
MAUBAG_02124
dacF
MAUBAG_02126
MAUBAG_05359
rplL_2
MAUBAG_05361
Bacteria_small_SRP
Ser_GGA
Hptn
Mcos^T
dacF
cysJ
MCOS44829_00104
ffh
fdhF
map_2
MCOS44829_00108
MCOS44829_01309
MCOS44829_01310
MCOS44829_01311
Bacteria_small_SRP
Ser_GGA
Hptn
Mneo^T
dacF
cysJ_2
MNEO44074_02057
ffh
psrA
glnD
MNEO44074_02061
Hptn
Ser_GGA
Bacteria_small_SRP
MNEO44074_04916
dnaX
cfa
MtbH37Rv
RVBD_2913c
PknI
Hptn
ffh
RVBD_2917
GlnD
RVBD_2919c
DnaZ/X
Hptn
Bacteria_small_SRP
Ser_GGA
RVBD_3723
2 kb
