# Supplementary material for: Comparative genomics of Mycobacterium mucogenicum and Mycobacterium neoaurum clade members emphasizing tRNA and non-coding RNA
Source: BMC Evol Biol. 2019 Jun 18;19:124. doi: 10.1186/s12862-019-1447-7 (PMC6582537; doi:10.1186/s12862-019-1447-7)
Supplement: Supplementary file 4 — Introduction. Table and Figure legends. Table S7. Rfam non-coding RNA annotations. Figure S8a, b. ncRNA genes. Figure S9a-i. RNase P RNA (rnpB), tmRNA and 4.5S RNA. Figure S10. Northern blot analysis of selected tRNAs and ncRNAs with size markers. Figure S11a, b. Analysis of GOLLD RNA. Figure S12a-d. Comparison of Ms1 RNA and 6C RNA genes in mycobacteria. Figure S13a, b. Comparison of a intron Group II gene cluster. (ZIP 7530 kb) [file 12862_2019_1447_MOESM4_ESM.zip › 12862_2019_1447_MOESM4_ESM/Fig S9i.zip!/Fig S9f.pdf]

Fig S9f

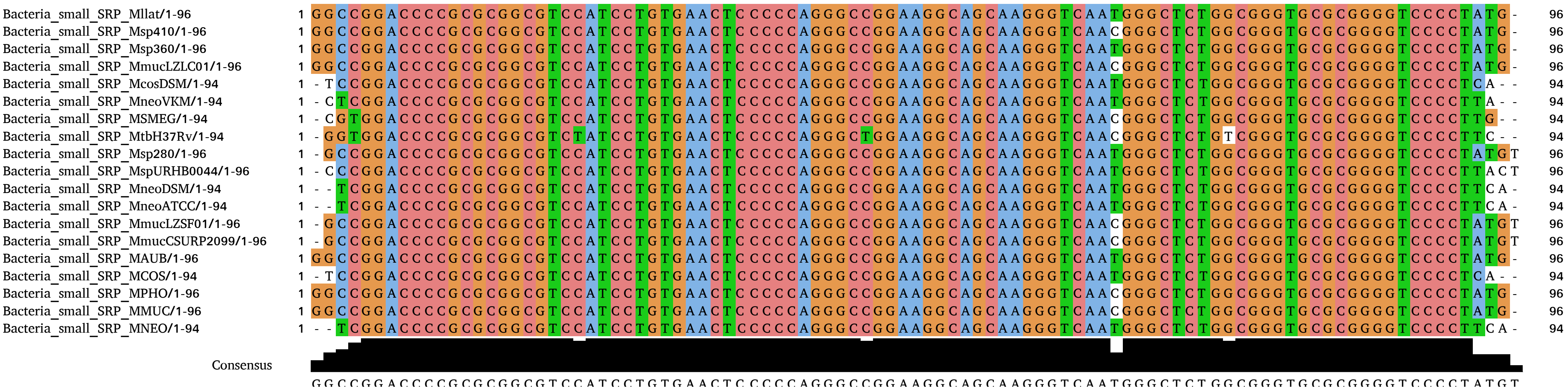
Bacteria_small_SRP_Mllat/1-96 1 GGCCGGACCCCGCGCGGCGTCCATCCTGTGAACTCCCCAGGGCCGGAAGGCAGCAAGGGTCAATGGGCTCTGGCGGGTGCGCGGGGTCCCCTATG- 96
Bacteria_small_SRP_Msp410/1-96 1 GGCCGGACCCCGCGCGGCGTCCATCCTGTGAACTCCCCAGGGCCGGAAGGCAGCAAGGGTCAACGGGCTCTGGCGGGTGCGCGGGGTCCCCTATG- 96
Bacteria_small_SRP_Msp360/1-96 1 GGCCGGACCCCGCGCGGCGTCCATCCTGTGAACTCCCCAGGGCCGGAAGGCAGCAAGGGTCAATGGGCTCTGGCGGGTGCGCGGGGTCCCCTATG- 96
Bacteria_small_SRP_MmucLZLC01/1-96 1 GGCCGGACCCCGCGCGGCGTCCATCCTGTGAACTCCCCAGGGCCGGAAGGCAGCAAGGGTCAACGGGCTCTGGCGGGTGCGCGGGGTCCCCTATG- 96
Bacteria_small_SRP_McosDSM/1-94 1 -TCCGGACCCCGCGCGGCGTCCATCCTGTGAACTCCCCAGGGCCGGAAGGCAGCAAGGGTCAATGGGCTCTGGCGGGTGCGCGGGGTCCCCTCA-- 94
Bacteria_small_SRP_MneoVKM/1-94 1 -CTCGGACCCCGCGCGGCGTCCATCCTGTGAACTCCCCAGGGCCGGAAGGCAGCAAGGGTCAATGGGCTCTGGCGGGTGCGCGGGGTCCCCTTA-- 94
Bacteria_small_SRP_MSMEG/1-94 1 -CGTGGACCCCGCGCGGCGTCCATCCTGTGAACTCCCCAGGGCCGGAAGGCAGCAAGGGTCAACGGGCTCTGGCGGGTGCGCGGGGTCCCCTTG-- 94
Bacteria_small_SRP_MtbH37Rv/1-94 1 -GGTGGACCCCGCGCGGCGTCTATCCTGTGAACTCCCCAGGGCTGGAAGGCAGCAAGGGTCAACGGGCTCTGTCGGGTGCGCGGGGTCCCCTTC-- 94
Bacteria_small_SRP_Msp280/1-96 1 -GCCGGACCCCGCGCGGCGTCCATCCTGTGAACTCCCCAGGGCCGGAAGGCAGCAAGGGTCAATGGGCTCTGGCGGGTGCGCGGGGTCCCCTATGT 96
Bacteria_small_SRP_MspURHB0044/1-96 1 -CCCGGACCCCGCGCGGCGTCCATCCTGTGAACTCCCCAGGGCCGGAAGGCAGCAAGGGTCAATGGGCTCTGGCGGGTGCGCGGGGTCCCCTTACT 96
Bacteria_small_SRP_MneoDSM/1-94 1 --TCGGACCCCGCGCGGCGTCCATCCTGTGAACTCCCCAGGGCCGGAAGGCAGCAAGGGTCAATGGGCTCTGGCGGGTGCGCGGGGTCCCCTTCA- 94
Bacteria_small_SRP_MneoATCC/1-94 1 --TCGGACCCCGCGCGGCGTCCATCCTGTGAACTCCCCAGGGCCGGAAGGCAGCAAGGGTCAATGGGCTCTGGCGGGTGCGCGGGGTCCCCTTCA- 94
Bacteria_small_SRP_MmucLZSF01/1-96 1 -GCCGGACCCCGCGCGGCGTCCATCCTGTGAACTCCCCAGGGCCGGAAGGCAGCAAGGGTCAACGGGCTCTGGCGGGTGCGCGGGGTCCCCTATGT 96
Bacteria_small_SRP_MmucCSURP2099/1-96 1 -GCCGGACCCCGCGCGGCGTCCATCCTGTGAACTCCCCAGGGCCGGAAGGCAGCAAGGGTCAACGGGCTCTGGCGGGTGCGCGGGGTCCCCTATGT 96
Bacteria_small_SRP_MAUB/1-96 1 GGCCGGACCCCGCGCGGCGTCCATCCTGTGAACTCCCCAGGGCCGGAAGGCAGCAAGGGTCAATGGGCTCTGGCGGGTGCGCGGGGTCCCCTATG- 96
Bacteria_small_SRP_MCOS/1-94 1 -TCCGGACCCCGCGCGGCGTCCATCCTGTGAACTCCCCAGGGCCGGAAGGCAGCAAGGGTCAATGGGCTCTGGCGGGTGCGCGGGGTCCCCTCA-- 94
Bacteria_small_SRP_MPHO/1-96 1 GGCCGGACCCCGCGCGGCGTCCATCCTGTGAACTCCCCAGGGCCGGAAGGCAGCAAGGGTCAACGGGCTCTGGCGGGTGCGCGGGGTCCCCTATG- 96
Bacteria_small_SRP_MMUC/1-96 1 GGCCGGACCCCGCGCGGCGTCCATCCTGTGAACTCCCCAGGGCCGGAAGGCAGCAAGGGTCAACGGGCTCTGGCGGGTGCGCGGGGTCCCCTATG- 96
Bacteria_small_SRP_MNEO/1-94 1 --TCGGACCCCGCGCGGCGTCCATCCTGTGAACTCCCCAGGGCCGGAAGGCAGCAAGGGTCAATGGGCTCTGGCGGGTGCGCGGGGTCCCCTTCA- 94
Consensus
GGCCGGACCCCGCGCGGCGTCCATCCTGTGAACTCCCCAGGGCCGGAAGGCAGCAAGGGTCAATGGGCTCTGGCGGGTGCGCGGGGTCCCCTATGT
