# Supplementary material for: Comparative genomics of Mycobacterium mucogenicum and Mycobacterium neoaurum clade members emphasizing tRNA and non-coding RNA
Source: BMC Evol Biol. 2019 Jun 18;19:124. doi: 10.1186/s12862-019-1447-7 (PMC6582537; doi:10.1186/s12862-019-1447-7)

Fig S14a

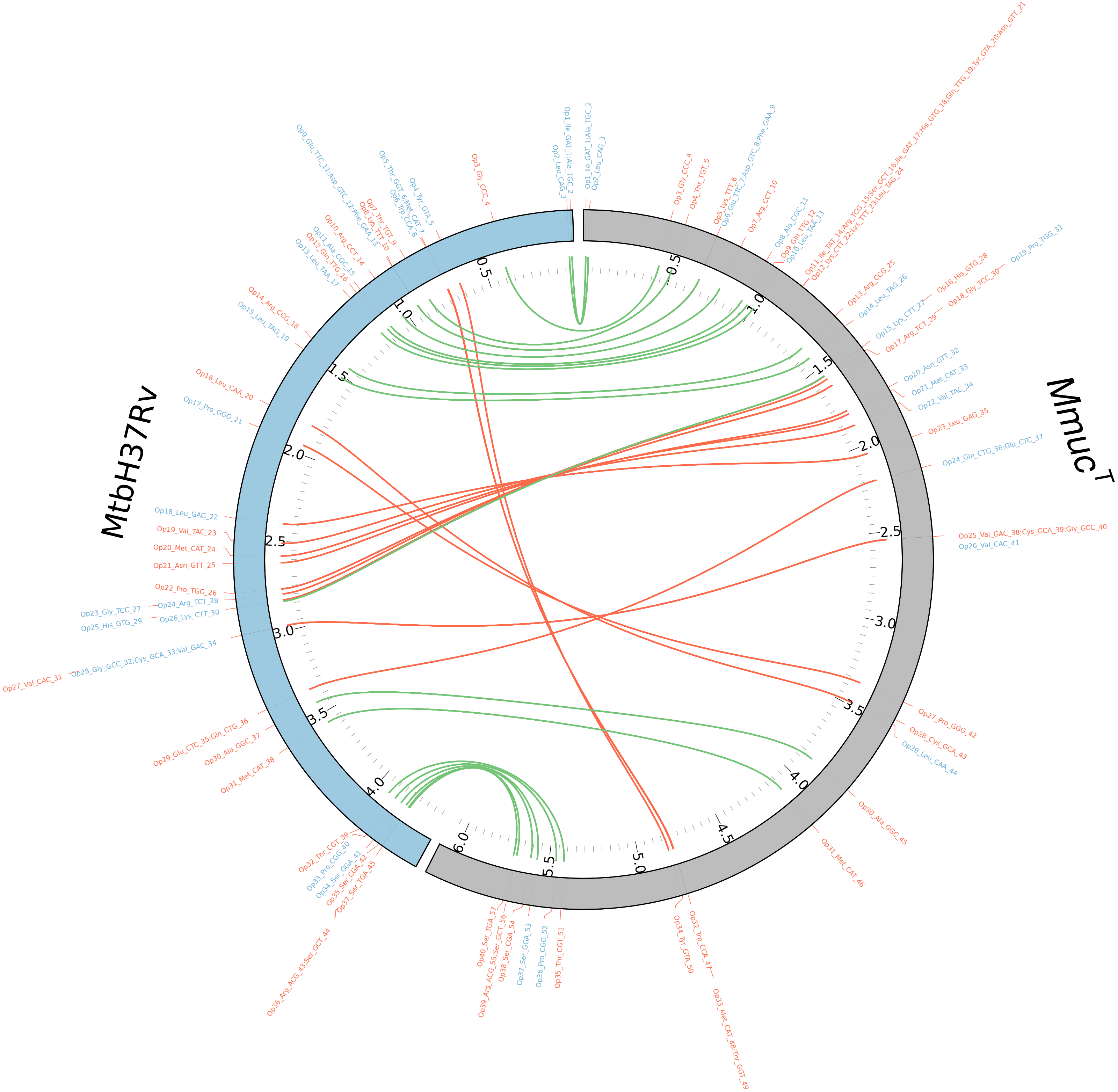

Supplement: Supplementary file 5 — Introduction. Figure legends. Figure S14a, b. Comparing positioning of tRNA genes in MmucT, MtbH37Rv and MsmegMC2-155. Figure S15a, b. Frequency of the identity of the nucleobase at position -1 in tRNA genes [35]. (ZIP 3300 kb) [file 12862_2019_1447_MOESM5_ESM.zip › 12862_2019_1447_MOESM5_ESM/Fig S14a.zip!/Fig S14a.pdf]
